# Construction and Validation of a Novel Cuproptosis-Related Seven-lncRNA Signature to Predict the Outcomes, Immunotherapeutic Responses, and Targeted Therapy in Patients with Clear Cell Renal Cell Carcinoma

**DOI:** 10.1155/2023/7219794

**Published:** 2023-01-25

**Authors:** Haoxun Zhang, Zikuan Ning, Bowen Wang, Yiwen Liu, Boju Tao, Guoling Zhang, Hua Liu, Chunyang Wang

**Affiliations:** First Affiliated Hospital of Harbin Medical University, Harbin, China

## Abstract

**Background:**

Cuproptosis was recently recognized as a novel form of cell death, linked closely to the occurrence and progression of cancer. We aimed to identify prognostic cuproptosis-related long noncoding RNAs (lncRNAs) and build a risk signature to predict the prognosis and treatment responses of clear cell renal cell carcinoma (ccRCC) in this work.

**Methods:**

LASSO–Cox regression was conducted to construct the signature based on prognostic cuproptosis-related lncRNAs (CR-lncRNAs). The signature's reliability and sensitivity were assessed by the Kaplan-Meier survival analysis and receiver operating characteristic analysis. External validation was performed via data from the International Cancer Genome Consortium database. On the basis of CR-lncRNAs, an lncRNA-microRNA-mRNA regulatory network was created, and functional enrichment analysis was used to investigate the underlying biological roles of these genes. In addition, the relationship between the risk signature and immunotherapy and targeted therapy responses was examined. Finally, the expression levels of seven candidate lncRNAs between tumor and normal cells were compared in vitro using quantitative real-time PCR.

**Results:**

A seven-CR-lncRNA risk signature was constructed, which showed a stronger potential for survival prediction than standard clinicopathological features in patients with kidney cancer. Functional enrichment analysis showed that the CR-lncRNA risk signature was enriched in ion transport-related molecular functions as well as various immune-related biological processes. Furthermore, we discovered that individuals in the high-risk group were more likely than those in the low-risk group to respond to immunotherapy and targeted therapies with medications like sunitinib and pazopanib. Finally, quantitative real-time PCR revealed that the expression levels of seven candidate lncRNAs differed significantly between RCC and healthy kidney cells.

**Conclusion:**

In summary, we generated a CR-lncRNA risk signature that may be utilized to predict outcomes in patients with ccRCC and responsiveness to immunotherapy and targeted treatment, potentially serving as a reference for clinical personalized medicine.

## 1. Introduction

Renal cell carcinoma (RCC) is a prevalent disease in adults that accounts for around 3% of all malignancies globally [1]. The most common histological subtype of RCC is clear cell renal cell carcinoma (ccRCC), which accounts for roughly 75% of all cases [2]. The selection of suitable treatment for patients with ccRCC based on the TNM classification of malignant tumor system and World Health Organization classification is critical [3]. However, with tumor heterogeneity, even in patients with identical clinical characteristics and treatment regimens, the prognosis of ccRCC may vary significantly, indicating that predicting risk stratification and prognosis according to current classification methods and clinicopathologic characteristics of patients is insufficient [4]. Therefore, it is crucial to identify novel risk signatures with stronger predictive capabilities to improve the prognosis of ccRCC.

Cuproptosis, a novel copper-dependent form of cell death proposed by Tsvetkov et al., is caused by the aggregation of mitochondrial lipoylated proteins and the destabilization of iron–sulfur cluster proteins triggered by the accumulation of intracellular copper [5, 6]. Several copper ionophores, such as elesclomol and disulfiram, have been pursued as cancer therapeutics to induce cuproptosis [7–9]. Recent studies revealed that the combination of copper and disulfiram copper selectively targeted and killed tumor cells, contributing to inhibiting tumor recurrence [10, 11]. Additionally, chemotherapy drugs combined with elesclomol showed a significant synergistic antitumor effect, which yielded nearly a doubling of median progression-free survival (PFS) and increased overall survival (OS) rates [12]. The researches above suggest that there are strong links between copper and cancer, and identifying the key regulators linked to cuproptosis is critical for expanding the treatment of ccRCC.

Long noncoding RNA (lncRNA) refers to a class of noncoding RNA whose length exceeds 200 nucleotides, accounting for almost 70% of the human transcriptome, which is essential in regulating the processes of physiology and pathology, especially those associated with cancer [13, 14]. lncRNAs are widely expressed and modulate the expression of messenger RNAs (mRNAs) that drive major cellular processes by functioning as competing endogenous RNAs [15]. Multiple studies have indicated that lncRNAs are linked closely to the OS of patients with RCC. The lncRNAs LSG1 and ENTPD3-AS1 suppress RCC via regulating epithelial splicing regulatory protein 2 ubiquitination and microRNA- (miRNA-) 155/hypoxia-inducible factor-1*α* signaling, respectively [16, 17]. The lncRNA GIHCG promotes RCC progression by modulating the miRNA-499a-5p/X-linked inhibitor of apoptosis axis [18]. However, the underlying mechanism of CR-lncRNAs in RCC is not well understood, and its role in predicting prognosis and therapeutic responses merits additional investigation.

In this work, we evaluated the prognostic significance of cuproptosis-related genes (CR-genes) in ccRCC and constructed a risk signature using CR-lncRNAs, with the goal of predicting the survival of patients with ccRCC and responsiveness to immunotherapy and targeted treatment. Furthermore, we validated the signature using the International Cancer Genome Consortium database and in vitro experiments and conducted a functional enrichment analysis to explore the potential mechanisms.

## 2. Materials and Methods

### 2.1. Data Acquisition

Gene expression data for ccRCC (*n* = 541) and paracancerous samples (*n* = 72) were downloaded from The Cancer Genome Atlas database (TCGA, http://cancergenome.nih.gov/). Considering the likelihood of noncancer mortality, we excluded patients with a follow-up period shorter than 30 days, leaving 515 ccRCC samples in the final cohort. Then, all ccRCC samples were randomly assigned to the testing and training sets in a 1 : 1 ratio for further analysis using the R software package “caret.” Meanwhile, transcriptional data and survival statistics from RCC (*n* = 91) were retrieved from the ICGC database (ICGC, http://icgc.org/) for external validation.

### 2.2. Analysis of CR-Genes in ccRCC

Searching published studies yielded a total of 19 CR-genes; all the genes involved in the cuproptosis mechanism mentioned in this article were included in our analysis, which were included in Supplementary Table S1. The “survival” package was then used to perform a Kaplan-Meier (K-M) survival analysis to further investigate the prognostic values of CR-genes in ccRCC. Subsequently, we downloaded the immunohistochemical staining images of selected prognosis-related genes from the Human Protein Atlas (HPA, http://www.proteinatlas.org/) in order to observe differences in expression at the protein level.

### 2.3. Identification of CR-lncRNAs

To distinguish between mRNAs and lncRNAs, gene transfer format annotation files were obtained from the Ensembl database (http://asia.ensembl.org/index.html). Then, correlation analysis was used to assess the coexpression relationships between lncRNAs and CR-genes using the R package “limma.” With |coefficient| > 0.4 and P<0.001 as our screening criteria, a total of 321 CR-lncRNAs were identified.

### 2.4. Construction and Estimation of the CR-lncRNA Prognosis Signature

Using univariate Cox regression analysis, a list of CR-lncRNAs was identified. Then, using the R package “glmnet,” LASSO regression was carried out on TCGA training set [19, 20]. Through multivariate Cox regression analysis, we identified seven lncRNAs that were significantly associated with prognosis, and a risk signature was generated subsequently. A risk score (RS) calculation algorithm was created according to the expression levels of seven CR-lncRNAs (Exp) and the corresponding regression coefficients (*β*) determined by the multivariate Cox regression:

(1) $$\begin{array}{c} \text{Risk score}=\overset{7}{\underset{i=7}{\sum }}\beta_{i}*{\text{Exp}}_{i}. \end{array}$$

Patients were classified by median RS (high-risk and low-risk groups). Time-dependent ROC curves (tROC) were generated to estimate the prediction sensitivity and specificity of the signature. In addition, the differences in OS between various clinicopathological characteristics classified by RS were compared in order to evaluate the diagnostic accuracy of the prognostic signature. Finally, a multivariate Cox regression analysis was conducted to determine whether the RS calculated by our signature could serve as a prognostic indicator for ccRCC patients.

### 2.5. Validation of Predictive Signature

TCGA testing and entire set and the ICGC data set served as internal and external validation, respectively. In the validation set, each patient was first awarded an RS based on our signature and then split into high- and low-risk subgroups using the training set's median RS. Subsequently, analyses such as K-M survival and tROC analysis were conducted to further validate the risk signature's predictive potential. In addition, using qRT-PCR, we validated the expression levels of seven potential lncRNAs and the impact of these lncRNAs on OS and PFS in ccRCC patients using K-M survival analysis.

### 2.6. Cell Culture

The Chinese Academy of Sciences Cell Resource Center provided us with the human RCC cell line 786-O, as well as normal control cells HK-2 (Shanghai, China). Procell Life Science and Technology Co., Ltd. provided the A498 and RC-2 cancer cells (Wuhan, China). The tumor cells were cultured in the Dulbecco's modified Eagle medium with 10% bovine serum albumin, 0.1 mg/mL streptomycin, and 100 U/mL penicillin (Gibco, Invitrogen, Carlsbad, CA, USA). The normal renal cells were cultured in RPMI-1640 medium with 10% bovine serum albumin, 100 U/mL penicillin, and 0.1 mg/mL streptomycin (Gibco, Invitrogen). All cells were cultured in a humidified incubator at 37°C with 5% CO_2_.

### 2.7. RNA Extraction and qRT-PCR Analysis

After two washes with cold phosphate buffered saline, total RNA was extracted from cells using the TRIzol RNA extraction reagent (Invitrogen, Waltham, MA, USA), as directed by the manufacturer. After that, a reverse transcription kit (Takara) was used to reverse the cDNA transcription. The expression of seven potential lncRNAs was assessed using SYBR Green qPCR. The qRT-PCR primer sequences are presented in Supplementary Table S2. GAPDH expression acted as an endogenous control. QuantStudio 3 (Thermo Fisher Scientific, Waltham, MA, USA) was used to perform the qRT-PCR, and the data were analyzed using the 2-*ΔΔ*CT method.

### 2.8. Establishment and Evaluation of Predictive Nomogram

To better predict survival at 1, 3, and 5 years, nomograms were generated using the “rms” R program. Using the R software package “survivalROC,” we compared prognostic nomograms with sex, age, American Joint Committee on Cancer (AJCC) stage, grade, and RS [21]. The same approach also validated our results by the testing set and the entire set.

### 2.9. Construction of Competing Endogenous RNA Regulatory Network and Functional Enrichment Analysis

Differentially expressed mRNAs (DEmRNAs) between subgroups were detected using the R package “limma” with thresholds of *p* < 0.05 and |Log2 FC| > 1. Using the “GSVA” R package, the enrichment scores of 16 immune cells and 10 immunological activities were assessed using a single-sample gene set enrichment analysis (ssGSEA). Using Cytoscape (V3.9.1), the coexpression network of CR-lncRNAs, miRNAs, and mRNAs was uncovered by drawing the competing endogenous (ceRNA) network. First, 5 of 27 prognostic-related lncRNAs were enrolled into the miRCode database to predict 163 target miRNAs. Then, 208 target mRNAs of 30 miRNAs were identified by integrating the results from the miRDB, miRTarBase, and TargetScan databases and the DEmRNAs. Finally, 208 target mRNAs from the ceRNA network were imported into Metascape to perform functional enrichment analysis.

### 2.10. Significance of the Signature in Immunotherapy and Targeted Therapy

Patients with ccRCC with somatic mutations were obtained from TCGA database. The tumor mutation burden (TMB), a biomarker for estimating the efficacy of immune therapy, is determined and compared between two distinct risk subgroups. Subsequently, all patients with ccRCC were divided into high- and low-TMB subgroups based on their median TMB value. K-M analysis was performed to determine the differences in survival between the four groups stratified by median RS and TMB values. To further investigate the relationship between RS and immunotherapy response, the expression levels of immune checkpoint molecules were compared across two distinct risk subgroups. In addition, we predicted the half-maximal inhibitory concentration (IC_50_) for four common targeted therapy drugs using the R package PRRophetic, which predicts drug sensitivity by building statistical models from tumor gene expression levels.

### 2.11. Statistical Analysis

All data analysis was performed using the Perl Data Language (http://www.perl.org/), R software (version 4.1.1, https://www.r-project.org/), and GraphPad version 8. The Wilcoxon rank-sum test was used for comparing differences between two groups, while the Kruskal–Wallis test was used to compare differences between two or more groups and *p* < 0.05 was considered to indicate a significant difference.

## 3. Results

**Figure 1:**
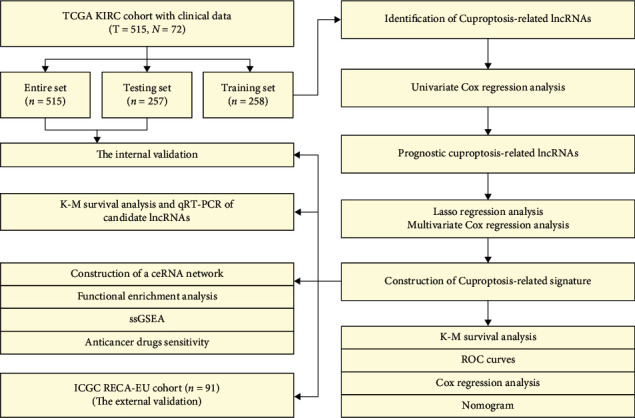
Flowchart of the study.

Figure 1 shows the flowchart of the current study. A total of 515 samples from TCGA database and 91 samples from the ICGC database were finally enrolled.  Table 1 summarizes the detailed clinicopathological characteristics of these samples.

### 3.1. Expression Profiles, IHC, and Survival Analysis of Cuproptosis-Related Genes in ccRCC

**Figure 2:**
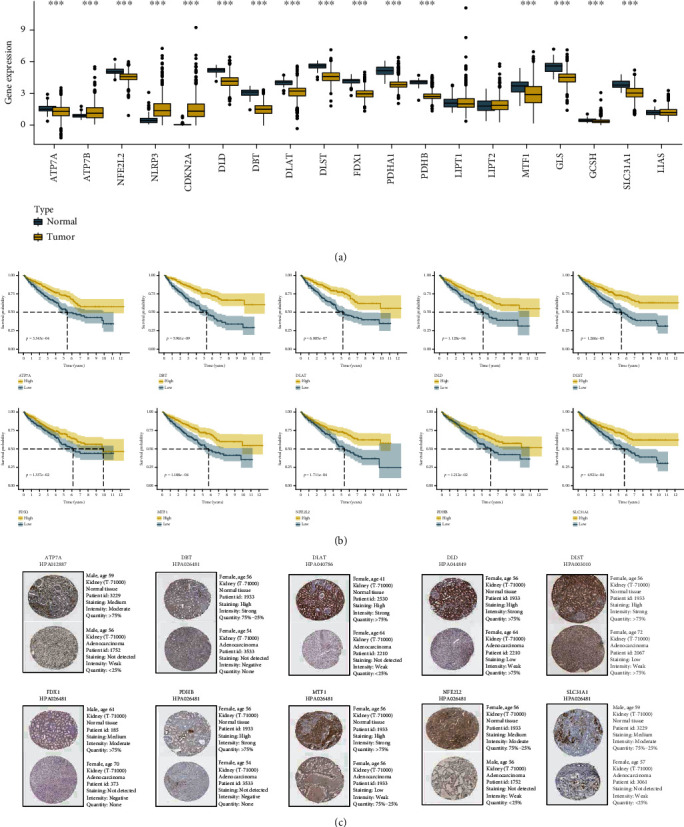
Identifying prognostic CR-genes and their expression levels in clear cell renal cell carcinoma: (a) expression comparison of 19 CR-genes in kidney tumor and normal tissues; (b) the association between 10 differentially expressed genes and clear cell renal cell carcinoma survival was investigated; (c) immunohistochemistry (IHC) images for 10 genes; ^∗^*p* < 0.05, ^∗∗^*p* < 0.01, and ^∗∗∗^*p* < 0.001.

We identified 19 CR-genes from published studies. The expression levels of these genes were compared between normal and tumor samples, and the results were shown in Figure 2(a); this showed that 13 genes, including ATP7A, NFE2L2, DLD, DBT, DLAT, DLST, FDX1, PDHA1, PDHB, MTF1, GLS, SLC31A1, and GCSH, were significantly downregulated in tumor tissues, while 3 genes, including ATP7B, NLRP3, and CDKN2A, were significantly upregulated (*p* < 0.001). The survival analysis of these differentially expressed genes was then performed to investigate their effects on OS and PFS in ccRCC patients. According to the K-M curves, low expression of 10 genes was associated with poor OS and PFS (Figure 2(b) and Figure S1). Supplementary Figure S2 depicted the K-M curves of six other genes with no statistically significant differences. The HPA database results showed that all ten prognostic CR-genes were overexpressed in normal tissues at the protein expression level (Figure 2(c)).

### 3.2. Construction of a Risk Signature Based on CR-lncRNAs

**Figure 3:**
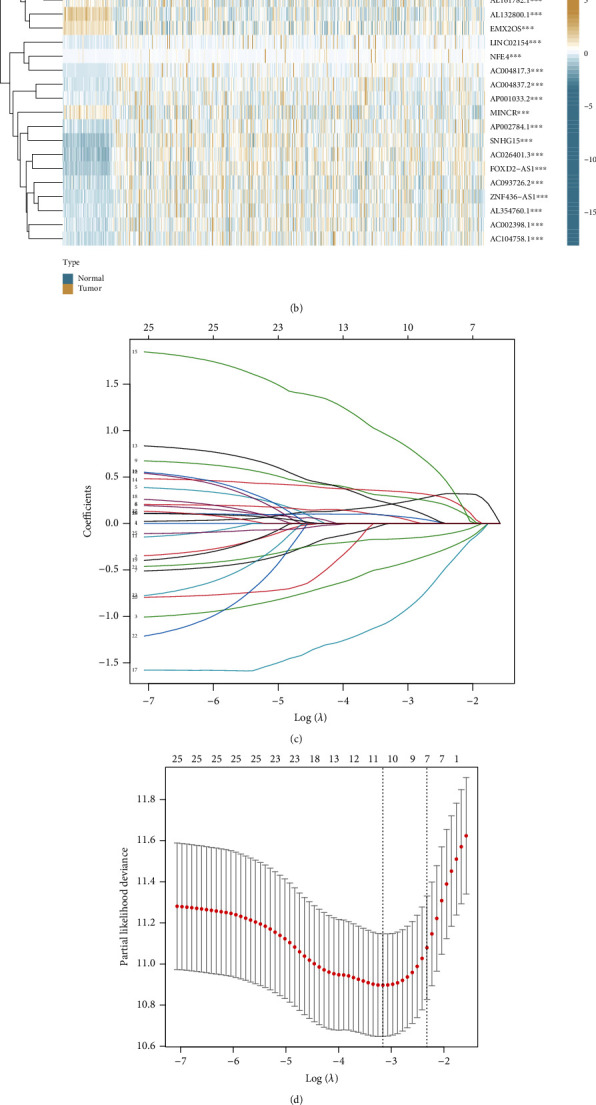
Construction of a novel seven-CR-lncRNA signature: (a) forest plot shows univariate Cox regression analysis of 27 CR-lncRNAs; (b) heatmap shows the expression profiles of 27 prognostic lncRNAs; (c) LASSO coefficient curves for 27 prognostic CR-lncRNAs; (d) options for the parameter (*λ*) by tenfold cross-validation; (e) multivariate Cox regression analysis of 7 lncRNAs screened using LASSO–Cox regression analysis; (f) cooccurrence and an exclusive association between candidate lncRNAs and CR-genes.

The matrix expression of 10 prognostic CR-genes and 16,876 lncRNAs was extracted. Subsequently, we conducted Pearson's correlation analysis and identified 321 CR-lncRNAs (|Pearson *R*| > 0.4 and *p* < 0.001). A Sankey diagram was used to depict the co-expression network of CR-lncRNAs (Figure S3). Next, 27 of the 321 CR-lncRNAs were screened that linked closely to the patient OS via a univariate Cox regression analysis (Figure 3(a)). A heatmap showed that the expression levels of 27 prognostic lncRNAs in tumor and normal tissues were significantly different (Figure 3(b)). LASSO–Cox regression analysis was implemented to simplify signatures, and 10 CR-lncRNAs were identified when partial likelihood deviance was at the minimum (Figures 3(c) and 3(d)). After multivariate Cox regression analysis, seven candidate lncRNAs significantly correlated with prognosis were recognized to construct a risk signature. Four lncRNAs, including MINCR, FOXD2-AS1, LINC02154, and AC004837.2, were risk factors (hazard ratio > 1), whereas the other three lncRNAs AL078581.2, SMARCA5-AS1, and LINC01671 were protective factors (hazard ratio < 1) (Figure 3(e)). The correlation between seven candidate lncRNAs and CR-genes is shown in Figure 3(f). Based on the expression levels of seven candidate lncRNAs and their Cox coefficients, the RS of patients with ccRCC were calculated as follows:

(2) $$\begin{array}{c} \text{RS}=\left(0.442*{\text{Exp}}_{\text{MINCR}}\right)+\left(-0.622*{\text{Exp}}_{\text{AL}078581.2}\right)+\left(0.496*{\text{Exp}}_{\text{FOXD}2-\text{AS}1}\right)+\left(0.438*{\text{Exp}}_{\text{LINC}02154}\right)+\left(1.255*{\text{Exp}}_{\text{AC}004837.2}\right)+\left(-1.481*{\text{Exp}}_{\text{SMARCA}5-\text{AS}1}\right)+\left(-0.245*{\text{Exp}}_{\text{LINC}01671}\right). \end{array}$$

### 3.3. Evaluating and Validating the Risk Signatures

**Figure 4:**
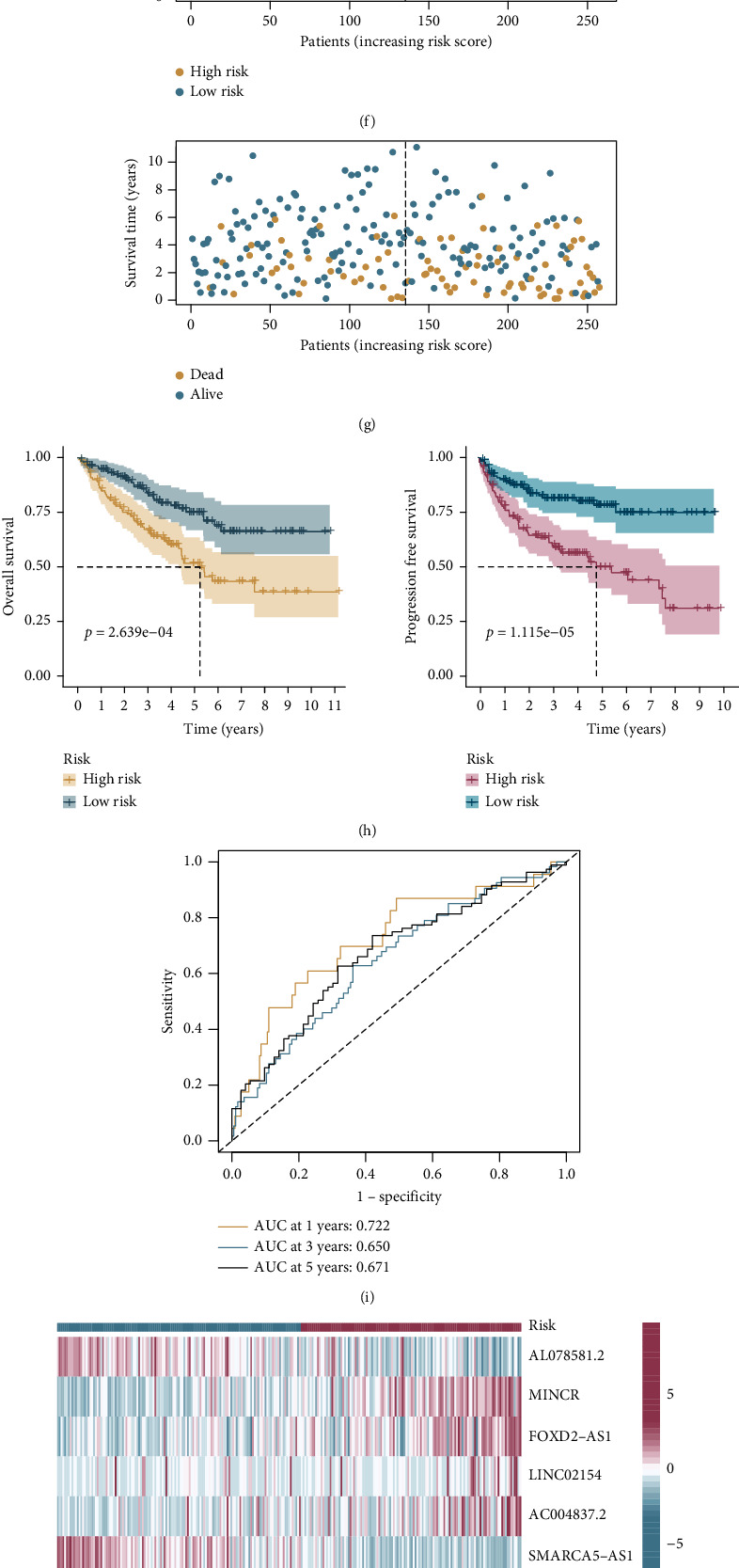
Estimation and internal validation of the risk signature of seven lncRNAs: (a) the risk score (RS) was significantly correlated with survival rate; (b) scatter plots of patient survival status; (c) the Kaplan-Meier curves exhibited that the RS was significantly associated with the overall survival (OS) and progression-free survival (PFS); (d) receiver operating characteristic (ROC) curve of the seven-lncRNA risk signature; (e) heatmap of seven candidate lncRNAs between two risk groups; (f–j) the risk curves, scatter plots, heatmaps, survival curves, and ROC curves of the internal validation sets; (k–o) the entire set.

The risk curves and scatter plots demonstrated that the quantity of deaths increased as RS increased (Figures 4(a) and 4(b)). The OS and PFS of high-risk patients were significantly shorter than in low-risk patients, according to the K-M curves (Figure 4(c)). ROC curves were used to evaluate the predictive value of the risk signature (Figure 4(d)). The area under the ROC curve (AUC) values of 1-, 3-, and 5-year OS for the training sets were 0.760, 0.785, and 0.838.  Figure 4(e) presents the expression heatmap of these seven candidate lncRNAs in two different risk groups. Risk factors including MINCR, FOXD2-AS1, LINC02154, and AC004837.2 were overexpressed in the high-risk group, whereas AL078581.2, SMARCA5-AS1, and LINC01671, as protective factors, were overexpressed in the low-risk group.

The accuracy of the risk signature was further validated by the internal validation (the testing) set and the entire set (Figures 4(f)–4(o)). Consistent with the results of training sets, in the internal validation set and entire data set, the probability of death was higher in patients with high RS. The AUC values of 1-, 3-, and 5-year OS were 0.722, 0.650, and 0.671 for the internal validation sets and 0.741, 0.713, and 0.754 for entire sets, respectively. To summarize, our seven-lncRNA risk signature performed well in predicting the prognosis for patients with ccRCC.

### 3.4. Subset Analysis of the Prognostic Value of the Risk Signature

**Figure 5:**
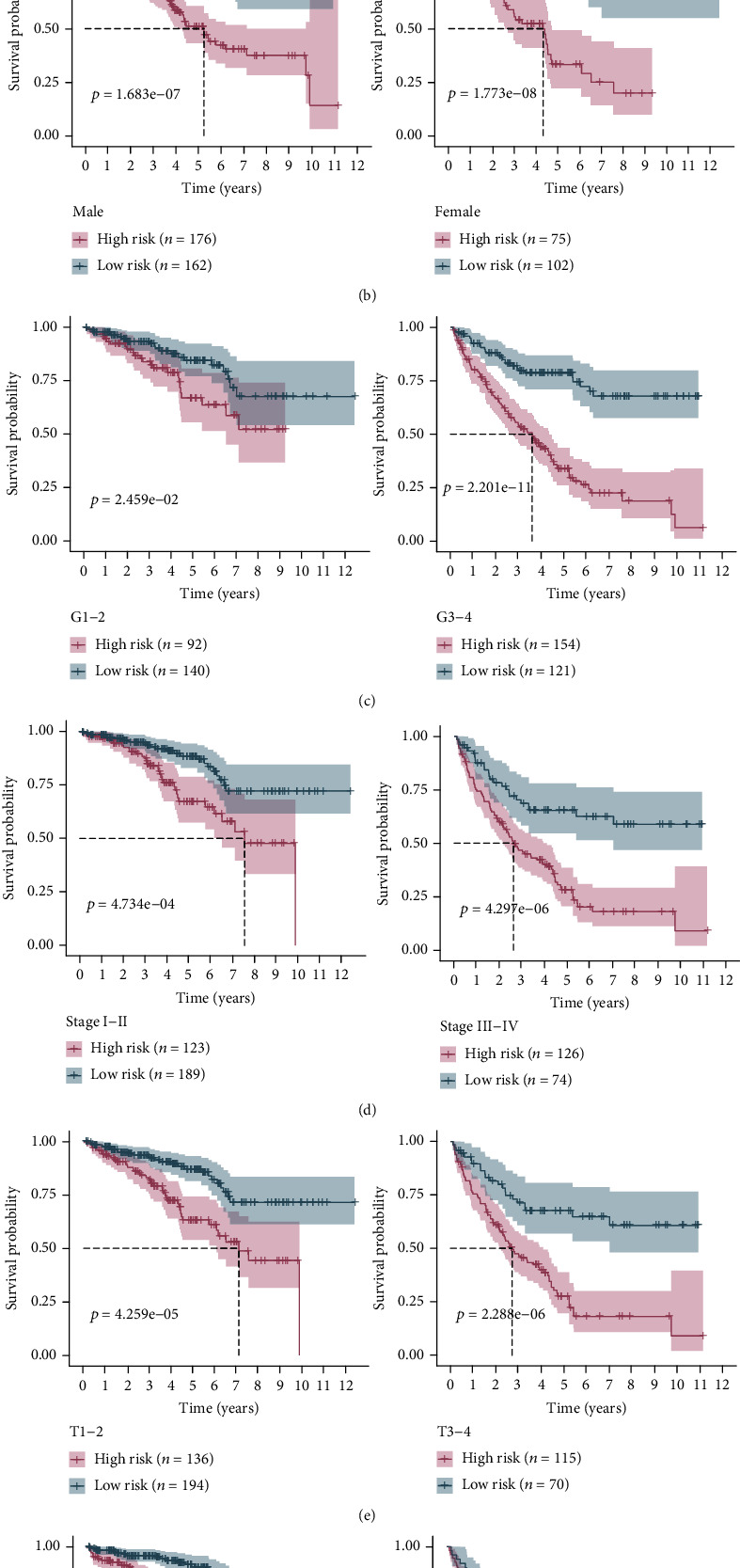
The Kaplan-Meier survival curves for the risk score (RS) level in different subgroups: (a) younger, older; (b) male, female; (c) lower grade, higher grade; (d) stages 1–2, stages 3–4; (e) T1–2, T3–4; (f) without or with recurrence.

For further investigating the relationship between the risk signature and the prognosis of patients with ccRCC, a stratification analysis was performed by examining a variety of clinicopathological factors, including gender, age, grade, AJCC stage, pathological T stage, and recurrence (Figure 5). Patients with high RS had a worse prognosis than patients with low RS, whether they were younger (≤60 years, *p* = 5.691*E* − 09) or older (>60 years, *p* = 1.392*E* − 05), male (*p* = 1.683*E* − 07) or female (*p* = 1.773*E* − 08), lower grade (grades 1–2, *p* = 2.459*E* − 02) or higher grade (grades 3–4, *p* = 2.201*E* − 11), stages 1–2 (*p* = 4.734*E* − 04) or stages 3–4 (*p* = 4.297*E* − 06), T1–2 (*p* = 4.259*E* − 05) or T3–4 (*p* = 2.288*E* − 06), and without recurrence (*p* = 7.539*E* − 05) or with recurrence (*p* = 6.926*E* − 04). In each stratification of gender, age, stage, T stage, and recurrence, the results showed that our risk profile remained a reliable tool for predicting ccRCC survival.

### 3.5. Construction and Validation of the Prognostic Predictive Nomogram

**Figure 6:**
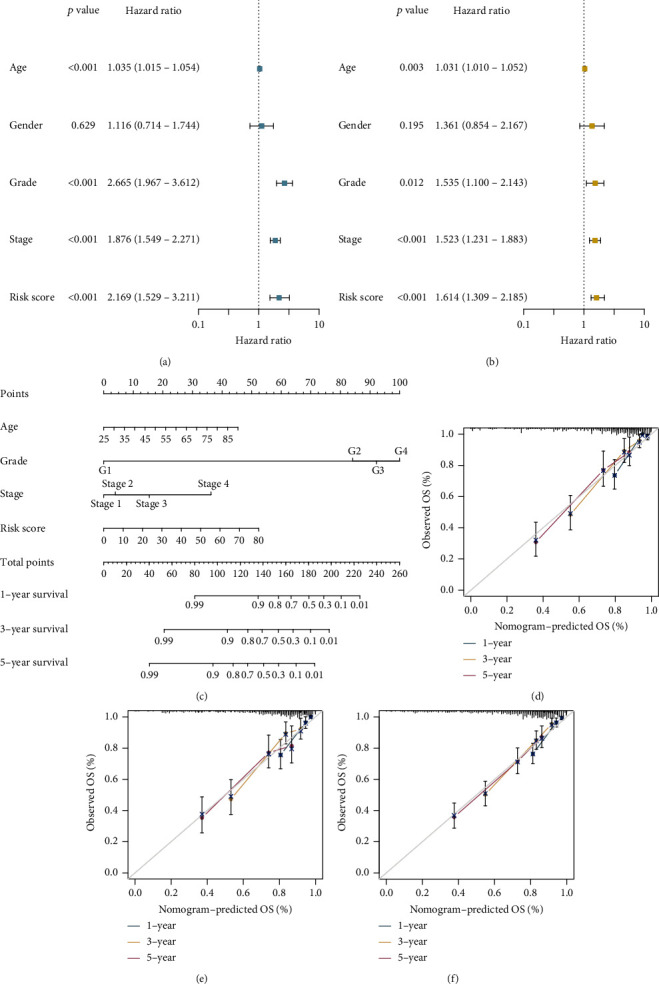
Development of a prediction nomogram using independent prognostic factors. (a, b) Univariate and multivariate Cox regression analyses are shown in forest plots. (c) Risk score, age, grade, and American Joint Committee on Cancer (AJCC) stage were used to create a nomogram. (d–f) The nomogram's calibration plot in the training set, internal validation set, and entire set.

**Figure 7:**
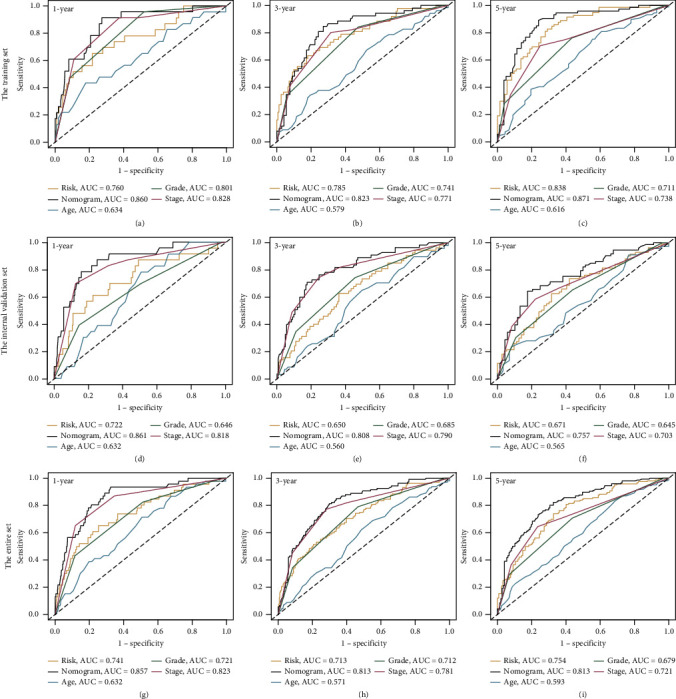
Comparison of risk score, age, grade, American Joint Committee on Cancer (AJCC) stage, and nomogram predictive performance: (a–c) receiver operating characteristic (ROC) curves in the training set for 1-, 3-, and 5-year overall survival prediction; (d–f) ROC curves in the internal validation set; (g–i) ROC curves in the entire set.

The Cox regression analysis was conducted to determine whether the seven-lncRNA risk signature could function as an independent prognostic predictor for ccRCC. Several available clinical parameters including age, gender, grade, AJCC stage, and our risk signature were included in the analysis. The results of the univariate and multivariate Cox regressions in both the training (Figures 6(a) and 6(b)) and validation sets (Figure S4) indicated that the seven-lncRNA risk signature, as well as other clinical factors such as age, grade, and AJCC stage, could be independent predictive criteria. Following that, a more reliable compound nomogram based on age, grade, AJCC stage, and RS was developed (Figure 6(c)). The calibration plots at 1, 3, and 5 years showed excellent agreement between the nomogram's anticipated survival probability and the actual survival outcome of patients (Figure 6(d)). Furthermore, when compared to other clinical factors such as RS, age, grade, and AJCC stage, nomogram points had the highest AUC values, implying that nomogram may be the best tool for predicting prognosis (Figures 7(a)–7(c)). The findings above were verified using the internal validation set and the entire set. At 1, 3, and 5 years, the calibration plots of two validation sets also exhibited excellent consistency between nomogram predictions and actual survival probabilities (Figures 6(e) and 6(f)). In addition, the ROC curves demonstrated that the nomogram points no matter in which set possessed better prediction performance than the RS and other clinical parameters (Figures 7(d)–7(i)).

### 3.6. External Validation of Risk Signature

**Figure 8:**
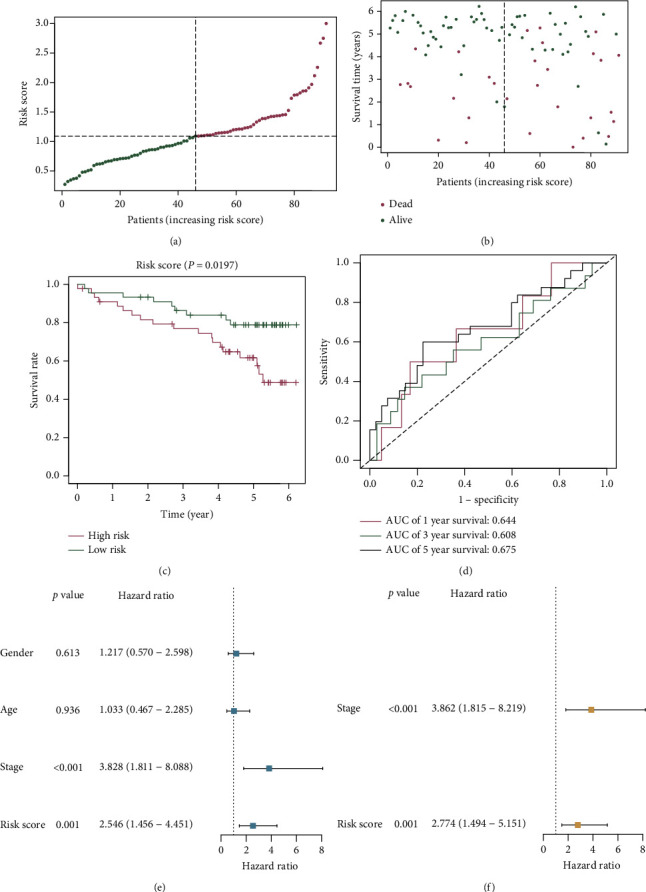
Validation of CR-lncRNA risk signature using the International Cancer Genome Consortium (ICGC) database: (a) risk curve; (b) survival status scatter plot; (c) Kaplan-Meier survival curves; (d) time-dependent receiver operating characteristic (tROC) analysis; (e, f) forest plots exhibiting the results of the univariate and multivariate Cox regression analyses.

Data from the ICGC database functioned as the external validation set (*n* = 91) to further verify the robustness of the seven-lncRNA risk signature. The RS of the ICGC patients was calculated using our risk signature, and all patients were then split into two subgroups based on the median RS from TCGA training set. Patients with higher RS had a significantly lower survival rate than those with lower RS (Figures 8(a)–8(c)). According to the tROC analysis, the risk signature's AUC values were 0.644 in the first year, 0.608 in the third year, and 0.675 in the fifth year (Figure 8(d)). Furthermore, the Cox regression analyses revealed that the RS could be used as an independent prognostic predictor for ICGC database patients (Figures 8(e) and 8(f)).

### 3.7. Verification of Seven Candidate CR-lncRNAs

**Figure 9:**
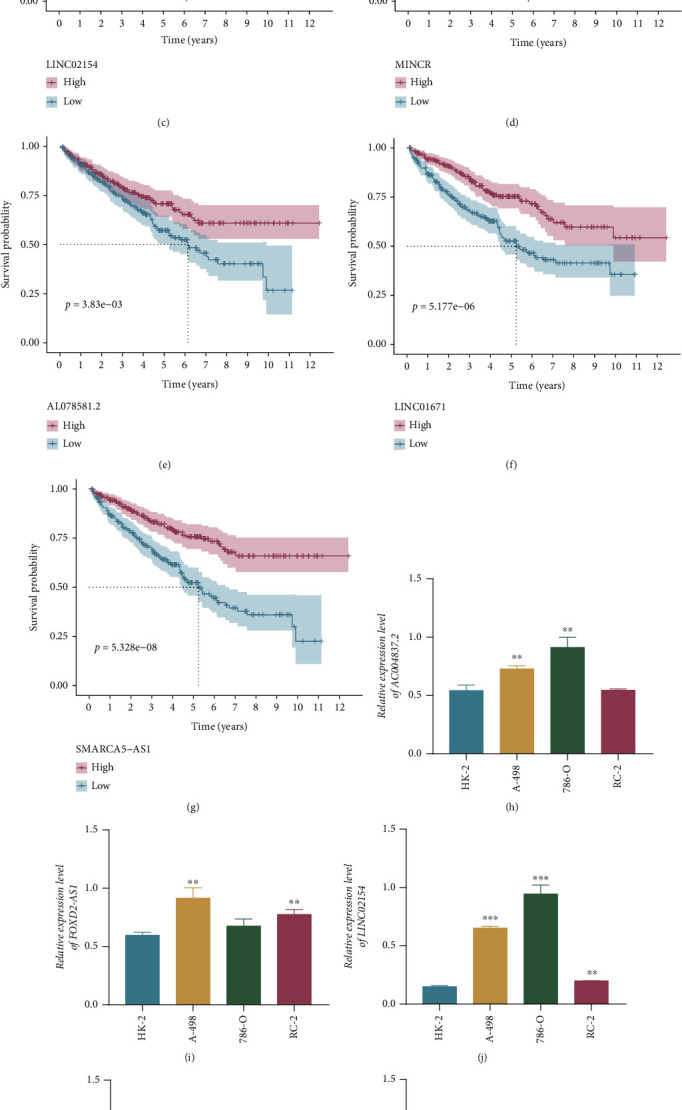
Survival analysis and expression levels of seven candidate CR-lncRNAs. (a–g) The Kaplan-Meier survival analysis of seven candidate CR-lncRNAs. (h–n) The expression of the seven candidate CR-lncRNAs in human kidney normal cell and three kidney cancer cell lines. ^∗^*p* < 0.05, ^∗∗^*p* < 0.01, and ^∗∗∗^*p* < 0.001.

The K-M survival curves certified that higher expression levels of four risk factors, including AC004837.2, FOXD2-AS1, LINC02154, and MINCR, were associated with lower overall survival rates and PFS (Figures 9(a)–9(d) and Figure S5). On the contrary, higher expression levels of AL078581.2, LINC01671, and SMARCA5-AS1, identified as three protective factors, corresponded with better OS and PFS in ccRCC (Figures 9(e)–9(g) and Figure S5). Additionally, the expression levels of seven CR-lncRNAs were determined using qRT-PCR in kidney normal and cancer cells. The results showed that the expression levels of AC004837.2, FOXD2-AS1, LINC02154, and MINCR were significantly increased in kidney cancer cells compared with normal cells, whereas AL078581.2, LINC01671, and SMARCA5-AS1 were downregulated in kidney cancer cells (Figures 9(h)–9(n)).

### 3.8. Competing Endogenous RNA Regulatory Network and Functional Enrichment Analysis

**Figure 10:**
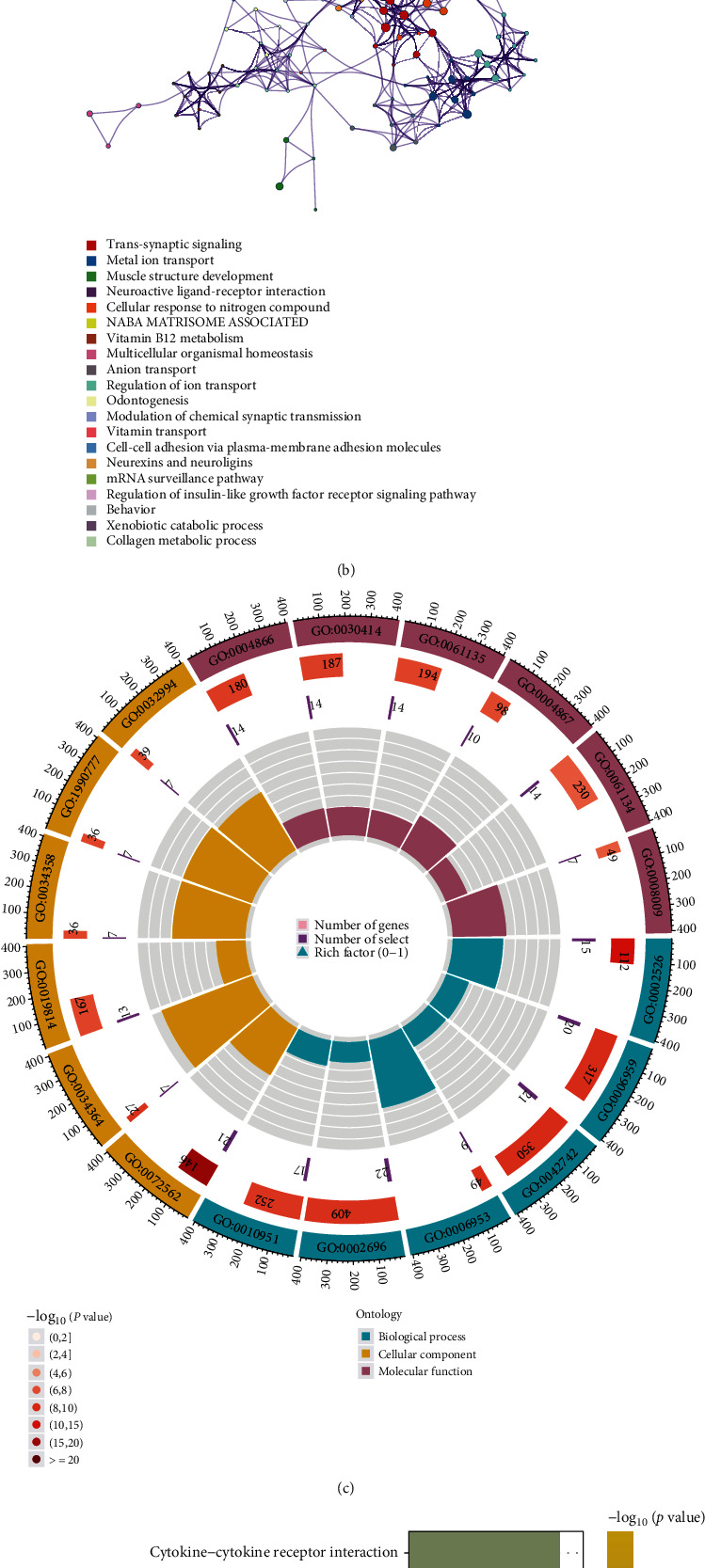
Functional enrichment analysis: (a) bar graph of enriched terms across the 208 target mRNAs; (b) network of enriched terms colored according to cluster ID; (c, d) Gene Ontology (GO) and Kyoto Encyclopedia of Genes and Genomes (KEGG) enrichment analyses of differentially expressed genes (DEGs) between high- and low-risk groups.

Subsequently, a ceRNA regulatory network was constructed to explore how the CR-lncRNAs regulate gene expression by functioning as miRNA sponges in ccRCC. Based on the 27 prognostic CR-lncRNAs above, 5 lncRNAs and 30 target miRNAs were extracted from the miRcode database. Then, a total of 208 mRNAs were identified by integrating the DEmRNAs and the results from the miRDB, miRTarBase, and TargetScan databases. Ultimately, a ceRNA network comprising 5 lncRNAs, 27 miRNAs, and 208 mRNAs was constructed (Figure S6 and Supplementary Table S3). The online tool Metascape was used to explore the biological functions of the 208 target mRNAs. The results showed that several ion transport-related molecular functions, including metal ion transport, anion transport, and regulation of ion transport, were enriched, which suggested that these lncRNAs may affect the process of cuproptosis (Figures 10(a) and 10(b) and Supplementary Table S4).

To elucidate the underlying pathways between the high- and low-risk subgroups, a total of 420 differentially expressed genes (DEGs) were identified and the biological functions of DEGs were explored using GO and KEGG enrichment analyses (Supplementary Table S5). The results of GO showed that the genes were enriched in positive regulation of leukocyte activation, blood microparticle, defense response to bacteria, humoral immune response, negative regulation of endopeptidase activity, and other factors (Figure 10(c) and Table 2). In the KEGG pathway analysis, cytokine–cytokine receptor interaction, viral protein interaction with cytokine and cytokine receptors, complement and coagulation cascades, and the nuclear factor kappa-light-chain-enhancer of activated B cells (NF-kappa B) and interleukin- (IL-) 17 signaling pathways were identified (Figure 10(d)).

### 3.9. Prediction of Cancer Immunotherapy Response Using the Risk Signature

**Figure 11:**
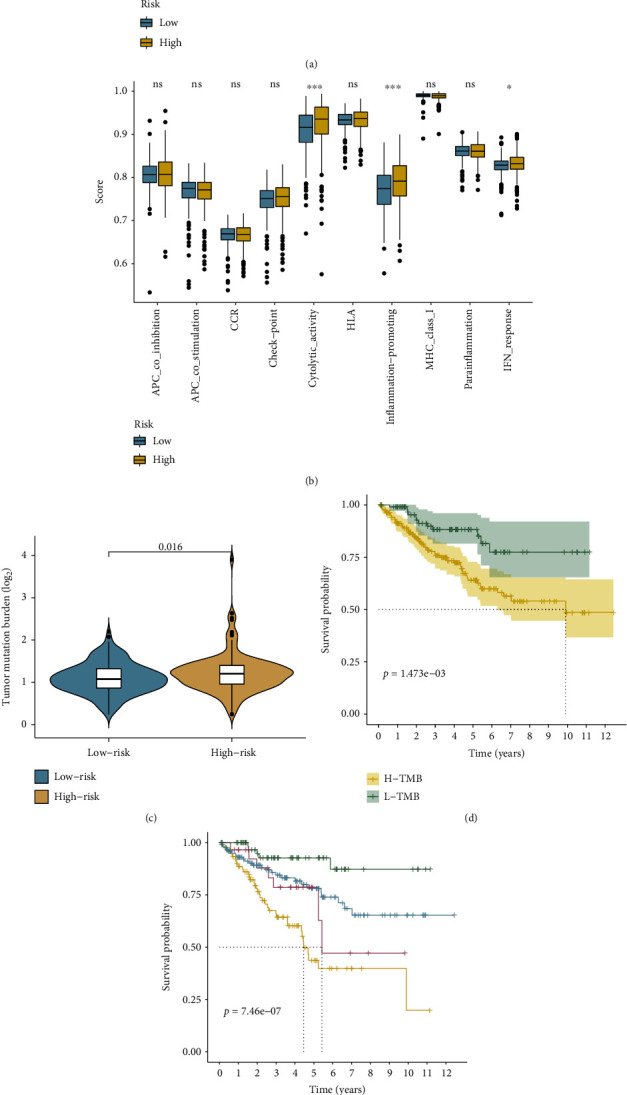
The role of risk signature in immunotherapy: (a) comparison of the single-sample gene set enrichment analysis (ssGSEA) scores between two subgroups of 16 immune cells and (b) 10 immune-related functions; (c) the tumor mutation burden (TMB) and CR-lncRNA risk signature are connected; (d) survival curves for groups with high and low TMB; (e) survival curves in four patient groups, stratified by signature and TMB; (f) differential expression of immune checkpoint molecules in two risk groups.

The enrichment scores of ssGSEA for 16 immune cell subpopulations and 10 immune-related activities or pathways were quantified to explore the link between the RS and immune status, which may contribute to determining whether the signature could serve for predicting immunotherapy responses. The results showed that B cells, CD8^+^ T cells, immature dendritic cells, natural killer cells, mast cells, neutrophils, T follicular helper cells, type 1 T helper cells, and tumor-infiltrating lymphocytes were significantly different between two subgroups (Figure 11(a)). Moreover, immune activities such as the interferon (IFN) response, cytolytic activity, and inflammation-promoting function were stronger in the high-risk group, implying that patients with immunity suppression in the high-risk group could benefit from immunotherapy (Figure 11(b)).

TMB values, serving as a biomarker for evaluating immune checkpoint inhibitor (ICI) efficiency, were calculated in the present study. The TMB values in the high-risk group were higher than in the low-risk group, as shown in Figure 11(c), suggesting that patients with high RS may be more likely to respond to ICI therapy. Furthermore, based on the TMB values and RS, the patients were divided into four subgroups. The survival probability of patients in the high-TMB groups was lower than patients in the low-TMB groups, and the prognosis of patients in the high-risk and high-TMB groups was the poorest of the four groups (Figures 11(d) and 11(e)).

Finally, to confirm the findings above, we investigate the relationship between the risk signature and the expression levels of immune checkpoint genes such as PD-1, CTLA-4, LAG3, TIGIT, and LMTK3. These findings suggest that the CR-lncRNA risk signature could be used as a biomarker to predict immunotherapy responses.

### 3.10. Correlation between the Risk Signature and Response to Targeted Therapy

**Figure 12:**
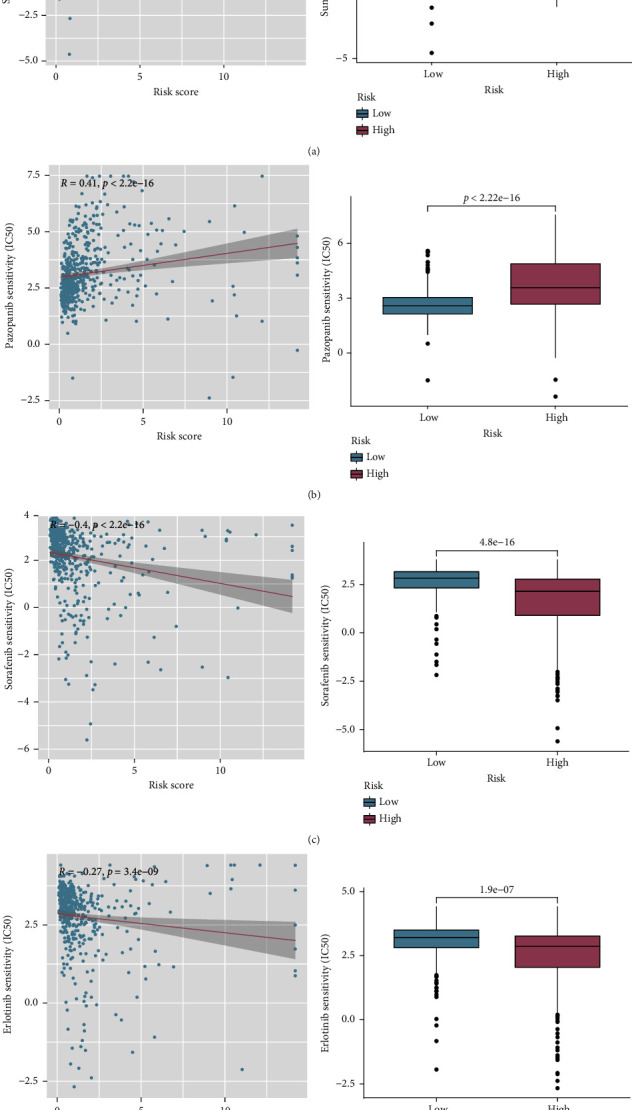
Prediction of sensitivity to common targeted therapeutic drugs: (a, b) scatter plots and boxplots demonstrating the IC_50_ of sunitinib and pazopanib; (c, d) scatter plots and boxplots demonstrating the IC_50_ of sorafenib and erlotinib.

We investigated the differences in response to four representative targeted therapy medicines, including sunitinib, sorafenib, pazopanib, and erlotinib, between two different risk subgroups. The estimated IC_50_ levels of these four targeted drugs in the two groups were compared and exhibited via scatter plots and boxplots. Sunitinib and pazopanib were discovered to be potential candidate medications for patients in the high-risk group (Figures 12(a) and 12(b)), whereas sorafenib and erlotinib may be more suitable for patients in the low-risk group (Figures 12(c) and 12(d)).

## 4. Discussion

Copper is a mineral nutrient that is essential for a host of cellular functions, such as mitochondrial respiration, antioxidant defense, and biosynthetic process [22]. In addition, many studies have shown that copper is also implicated in cancer cell growth, proliferation, and metastasis [23–28]. However, it is crucial that cellular copper is present in the right amount, and excess copper can cause cellular toxicity, leading to death [29]. Cuproptosis was first presented by Tsvetkov et al. to be different from other regulated cell death caused by oxidative stress; it is triggered by mitochondrial protein aggregation with the accumulation of intracellular copper [5]. Copper ionophores, such as disulfiram and elesclomol, have been proven to be effective in treating various cancers by inducing cuproptosis [7, 12, 30, 31]. In the present study, we found that a total of 10 key regulatory genes for cuproptosis correlated closely with the prognosis of patients with ccRCC, which showed that cuproptosis may have important implications for the occurrence and development of renal cell cancers.

Given the wide variation of prognostic outcomes of patients with ccRCC, developing a robust classifier model to stratify patients with varied risks and predict therapeutic drug responses is crucial. lncRNAs play a key role in various diseases, especially malignancies, the mutations and misregulation of which exhibit tumor-suppressive and -promoting (oncogenic) functions [31]. Considering the significant effect of cuproptosis and lncRNAs on prognosis of patients with cancer, we used LASSO regression and Cox regression analyses to construct a CR-lncRNA risk signature for patients with ccRCC, and we explored how signaling pathways are involved with the risk signature.

The OS and PFS survival curves demonstrated the power of the signature in predicting prognosis of patients with ccRCC. The risk signature could also be applied to various subsets, such as ages, gender, stage, grade, or tumor status, which may assist clinicians in making clinical decisions. The tROC analysis was performed, and the AUC of the risk signature was slightly smaller than that of the AJCC staging system, which was one of the most widely used classification systems for describing the extent of disease development in kidney cancer [32]. However, a predictive nomogram was composed based on the risk signature and showed superior specificity and sensitivity in predicting the patient's 1-, 3-, and 5-year survival probability in comparison with the AJCC stage. Moreover, the Cox regression analyses revealed that risk signatures could serve as independent prediction factors in ccRCC. Analyses were repeated with both the internal and external validation sets and yielded similar results, confirming the risk signature's predictive value.

Seven candidate lncRNAs linked closely to prognosis, including MINCR, FOXD2-AS1, LINC02154, AL078581.2, SMARCA5-AS1, AC004837.2, and LINC01671, were identified and employed to construct the risk signature. The MYC-induced lncRNA MINCR is a newly identified lncRNA linked to MYC expression in the Burkitt lymphoma [33]. The overexpression of MINCR could trigger cancer-related gene alterations, causing disruptions in the cell cycle and growth factor signaling [34]. MINCR functions as an oncogenic gene in various malignancies. For example, MINCR aggravates colon cancer and glioma via the miR-708-5p-mediated Wnt/*β*-catenin pathway and miR-876-5p/GSPT1 axis, respectively [35, 36]. Moreover, MINCR is also overexpressed in hepatocellular carcinoma and gall bladder cancer, and its overexpression promotes cell proliferation, migration, and invasion [37, 38]. The lncRNA FOXD2-AS1 is abnormally expressed in a number of cancers and affects tumor progression [39]. Ni et al. revealed that FoxD2-AS1 regulates the miR-185-5P/HMGA2 axis and the PI3K/AKT signaling pathway to promote glioma progression [40]. LINC02154 is also overexpressed in tumor tissues, and Yue et al. found that LINC02154 overexpression leads to proliferation and metastasis of hepatocellular carcinoma via modulating cellular activities [41, 42]. In the present study, we conducted qRT-PCR and K-M survival analyses to confirm the effects of these genes in patients with RCC. Consistent with the findings above, four risk lncRNAs (MINCR, FOXD2-AS1, LINC02154, and AC004837.2) were overexpressed in renal cancer cells and correlated with poor OS and PFS in ccRCC. Some researchers have selected LINC01671 into their prognostic models as a risk factor to predict survival in lung adenocarcinoma and found no difference in lncRNA expression between tumor and normal tissues [43]. However, in our risk signature, LINC01671 is a protective gene, and the relative expression level is lower in cancer cells than healthy cells, implying that further confirmation is needed. The function and in-depth molecular interactions of the remaining three lncRNAs have not been reported in tumors, which merits further exploration.

Emerging evidence indicates that lncRNAs can function as ceRNAs by binding to miRNAs, thereby regulating target mRNA expression levels. It has been proposed that the lncRNA-miRNA-mRNA-ceRNA regulatory network plays an essential role in multiple types of cancer [44]. We created a ceRNA regulatory network to illustrate the relationship between CR-lncRNAs, their binding miRNAs, and target mRNAs in this study. The underlying biological activities and pathways of target mRNAs were then investigated using functional enrichment analysis. As expected, the results exhibited that these target mRNAs enriched several ion transport-related molecular functions, indicating that these lncRNAs may affect the process of cuproptosis. Furthermore, we carried out GO and KEGG analyses using the DEGs between two different risk groups and unexpectedly found that several immune-related biological functions and pathways were enriched. Therefore, it is reasonable to assume that cuproptosis might be linked closely to tumor immunity.

With few effective treatments for RCC other than surgical resection, many novel therapies, such as targeted therapies and ICI agents, have been emerging or available and show significant efficacy. Recently, ICI-based combinations (ICI-targeted agents or ICI–ICI) have gained widespread acceptance as an effective therapy and have been authorized as a first-line treatment for advanced RCC [45–47]. Therefore, identifying patients who are candidates for ICI in clinical practice is critical. The findings of ssGSEA indicated that, in the high-risk group, various immune functions were activated, including the IFN response, inflammation-promoting functions, and cytolytic activity. IFN was produced by activated natural killer and T cells, which are critical in enhancing the efficacy of ICI [48]. The cytolytic activity score has been proven to function as a biomarker for antitumor immunity and prognosis in patients with cancer. Wang et al. revealed that the cytolytic activity score was an independent unfavorable prognostic factor in glioma and was significantly positively linked to immune checkpoint expression, which is in accordance with our findings [49].

The TMB may be used to predict patient responses to immunotherapy and as a marker of immune checkpoint inhibitor treatment failure in a variety of cancers [50]. Patients in the high-risk group had a greater TMB value than those in the low-risk group, suggesting that patients in the high-risk group are candidates for ICI treatment. Several popular immune checkpoint molecules, including PD-1, CTLA4, TIGIT, LAG3, and LMTK3, were also overexpressed in the high-risk groups, also supporting the argument that ICI treatment is suitable for patients with high-risk scores. Finally, we analyzed the sensitivity of four common targeted agents between two different risk groups, which may assist clinicians in making comprehensive and personalized treatment plans for patients with ccRCC.

Some limitations of this study should be acknowledged. First, all data used to construct and validate the signature were downloaded from public databases. More real-world data of RCC cohorts should be collected to verify the accuracy and clinical utility of the signature. Second, more in vitro or in vivo experiments are needed subsequently to find out the potential functions of the screened lncRNAs in cuproptosis.

## 5. Conclusions

In summary, a risk signature composed of seven prognostic CR-lncRNAs and a predictive nomogram based on the signature were constructed for the sake of predicting outcomes for patients with ccRCC. The predictive power of the nomogram was proven superior to that of the conventional AJCC staging. In addition, our risk signature could predict the responses to targeted therapy and immunotherapy, which is critical for relieving patient suffering, enhancing treatment effectiveness, and reducing healthcare costs.

## Figures and Tables

**Table 1:** Characteristics of all eligible ccRCC patients from TCGA and ICGC databases.

Covariates	Entire set	Testing set	Training set	ICGC cohort
	*n* = 515	*n* = 257	*n* = 258	*n* = 91
Age, years	60.6 ± 12.1	60.9 ± 12.2	59.7 ± 11.9	60.6 ± 10.1
≤ 65, *n* (%)	341 (66.2)	166 (65.0)	174 (67.4)	63 (69.2)
> 65, *n* (%)	174 (33.8)	90 (35.0)	84 (32.6)	28 (30.8)
Gender, *n* (%)				
Female	177 (34.4)	89 (34.6)	88 (34.1)	39 (42.9)
Male	338 (65.6)	168 (65.4)	170 (65.9)	52 (57.1)
Grade, *n* (%)				
G1	12 (2.3)	6 (2.3)	6 (2.3)	NA
G2	220 (42.7)	113 (44.0)	107 (41.5)	NA
G3	201 (39.0)	91 (35.4)	110 (42.6)	NA
G4	74 (14.4)	43 (16.7)	31 (12.0)	NA
Unknown	8 (1.6)	4 (1.6)	4 (1.6)	NA
Stage, *n* (%)				
Stage I	256 (49.7)	135 (52.5)	121 (47.0)	NA
Stage II	56 (10.9)	28 (10.9)	28 (10.9)	NA
Stage III	117 (22.7)	48 (18.7)	69 (26.7)	NA
Stage IV	83 (16.1)	44 (17.1)	39 (15.1)	NA
Unknown	3 (0.6)	2 (0.8)	1 (0.3)	NA
T, *n* (%)				
T1	262 (50.9)	138 (53.7)	124 (48.1)	54 (59.3)
T2	68 (13.2)	34 (13.2)	34 (13.2)	13 (14.3)
T3	174 (33.8)	81 (31.5)	93 (36.0)	22 (24.2)
T4	11 (2.1)	4 (1.6)	7 (2.7)	2 (2.2)
N, *n* (%)				
N0	230 (44.7)	117 (45.5)	113 (43.8)	79 (86.8)
N1	16 (3.1)	7 (2.7)	9 (3.5)	2 (2.2)
Unknown	269 (52.2)	133 (51.8)	136 (52.7)	10 (11.0)
M, *n* (%)				
M0	408 (79.2)	205 (79.8)	203 (78.7)	81 (89.0)
M1	79 (15.3)	42 (16.3)	37 (14.3)	9 (9.9)
Unknown	28 (5.5)	10 (3.9)	18 (7.0)	1 (1.1)

ccRCC: clear cell renal cell carcinoma.

**Table 2:** Results of Gene Ontology analysis.

Ontology	Description	*p* value
BP	Acute inflammatory response	3.12*E*-11
BP	Humoral immune response	1.38*E*-08
BP	Defense response to bacterium	1.42*E*-08
BP	Acute-phase response	1.73*E*-08
BP	Positive regulation of leukocyte activation	4.51*E*-08
BP	Negative regulation of endopeptidase activity	6.61*E*-08
CC	Blood microparticle	6.22*E*-16
CC	High-density lipoprotein particle	5.20*E*-08
CC	Immunoglobulin complex	4.37*E*-07
CC	Plasma lipoprotein particle	4.40*E*-07
CC	Lipoprotein particle	4.40*E*-07
CC	Protein-lipid complex	7.84*E*-07
MF	Endopeptidase inhibitor activity	1.47*E*-07
MF	Peptidase inhibitor activity	2.36*E*-07
MF	Endopeptidase regulator activity	3.70*E*-07
MF	Serine-type endopeptidase inhibitor activity	7.72*E*-07
MF	Peptidase regulator activity	2.82*E*-06
MF	Chemokine activity	3.77*E*-06

BP: biological process; CC: cellular component; MF: molecular function.

## Data Availability

The original contributions presented in the study are included in the article/Supplementary Material. Further inquiries can be directed to the corresponding author.

## Supplementary Materials

Supplementary 1 Table S1: a list of cuproptosis-related genes.

Supplementary 2 Table S2: the primer sequences of seven cuproptosis-related lncRNAs.

Supplementary 3 Figure S1: K-M curves exhibited the association between 10 differentially expressed genes and PFS of KIRC survival. (A) ATP7A; (B) DBT; (C) DLAT; (D) DLD; (E) DLST; (F) FDX1; (G) MTF1; (H) NFE2L2; (I) PDHB; (J) SLC31A1.

Supplementary 4 Figure S2: K-M curves of six genes. (A) ATP7B; (B) LIAS; (C) LIPT1; (D) LIPT2; (E) GCSH; (F) PDHA1.

Supplementary 5 Figure S3: a Sankey diagram showed the connection between the cuproptosis related lncRNAs and cuproptosis-related genes. Supplementary 5. Figure S4: forest plots exhibited the results of the Cox regression analyses of the 535 cuproptosis-related seven-lncRNA signature. (A, B) The univariate Cox regression analysis and multivariate Cox regression analysis in the testing set. (C, D) The univariate Cox regression analysis and multivariate Cox regression analysis in the entire set.

Supplementary 6 Figure S5: K-M curves exhibited the relationship between the expression levels of seven candidate lncRNAs and the PFS of KIRC patients. (A) LINC02154; (B) MINRC; (C) FOXD2-AS1; (D) AC004837.2; (E) SMARCA5-AS1; (F) LINC01671; (G) AL078581.2.

Supplementary 7 Figure S6: the ceRNA network of the five cuproptosis-related lncRNAs (orange) and their target miRNAs (light blue) and mRNAs (light green).

Supplementary 8 Table S3: the lncRNAs, miRNAs, and mRNAs used for construction for ceRNA network.

Supplementary 9 Table S4: biological functions of the 208 target mRNAs explored by the online tool Metascape.

Supplementary 10 Table S5: differentially expressed genes (DEGs) between the high- and low-risk subgroups.

Supplementary 11 R codes used in this research.
