# Supplementary material for: Construction and Validation of a Novel Cuproptosis-Related Seven-lncRNA Signature to Predict the Outcomes, Immunotherapeutic Responses, and Targeted Therapy in Patients with Clear Cell Renal Cell Carcinoma
Source: Dis Markers. 2023 Jan 25;2023:7219794. doi: 10.1155/2023/7219794 (PMC9893525; doi:10.1155/2023/7219794)
Supplement: Supplementary 1 — Table S1: a list of cuproptosis-related genes. [file 7219794.f1.docx]

| **Cuproptosis-related gene** | **Description** | [**Category**](https://www.genecards.org/Search/Keyword?queryString=NFE2L2&pageSize=25&startPage=0&sort=Category&sortDir=Ascending) |
| --- | --- | --- |
| NFE2L2 | NFE2 Like BZIP Transcription Factor 2 | Protein Coding |
| NLRP3 | NLR Family Pyrin Domain Containing 3 | Protein Coding |
| ATP7A | ATPase Copper Transporting Alpha | Protein Coding |
| ATP7B | ATPase Copper Transporting Beta | Protein Coding |
| SLC31A1 | Solute Carrier Family 31 Member 1 | Protein Coding |
| FDX1 | Ferredoxin 1 | Protein Coding |
| LIAS | Lipoic Acid Synthetase | Protein Coding |
| LIPT1 | Lipoyltransferase 1 | Protein Coding |
| LIPT2 | Lipoyl(Octanoyl) Transferase 2 | Protein Coding |
| DLD | Dihydrolipoamide Dehydrogenase | Protein Coding |
| DLAT | Dihydrolipoamide S-Acetyltransferase | Protein Coding |
| PDHA1 | Pyruvate Dehydrogenase E1 Subunit Alpha 1 | Protein Coding |
| PDHB | Pyruvate Dehydrogenase E1 Subunit Beta | Protein Coding |
| MTF1 | Metal Regulatory Transcription Factor 1 | Protein Coding |
| GLS | Glutaminase | Protein Coding |
| CDKN2A | Cyclin Dependent Kinase Inhibitor 2A | Protein Coding |
| DBT | Dihydrolipoamide Branched Chain Transacylase E2 | Protein Coding |
| GCSH | Glycine Cleavage System Protein H | Protein Coding |
| DLST | Dihydrolipoamide S-Succinyltransferase | Protein Coding |
