# Supplementary material for: Construction and Validation of a Novel Cuproptosis-Related Seven-lncRNA Signature to Predict the Outcomes, Immunotherapeutic Responses, and Targeted Therapy in Patients with Clear Cell Renal Cell Carcinoma
Source: Dis Markers. 2023 Jan 25;2023:7219794. doi: 10.1155/2023/7219794 (PMC9893525; doi:10.1155/2023/7219794)
Supplement: Supplementary 2 — Table S2: the primer sequences of seven cuproptosis-related lncRNAs. [file 7219794.f2.docx]

**Table S2.** The primer sequences of seven cuproptosis-related lncRNAs.

| **MINCR** | F primer (5′-3′) | CGCGTGTCTTCCGAACTCT |
| --- | --- | --- |
|  | R primer (5′-3′) | TCTGAGGTCTCTAGCGGGG |
| **FOXD2-AS1** | F primer (5′-3′) | GCCCAGAACAATTGGGAGGA |
|  | R primer (5′-3′) | AAGAGAGGGAGAGACGACCC |
| **LINC02154** | F primer (5′-3′) | TTCAGGCACAATCAGTGGGT |
|  | R primer (5′-3′) | GGAAGGACCCCTCCGAAAGA |
| **SMARCA5-AS1** | F primer (5′-3′) | CACTCCCAAACGTTCTGCTC |
|  | R primer (5′-3′) | TTGACCTCAGTGACCCGTCT |
| **LINC01671** | F primer (5′-3′) | TCCTGTGACAACAGGTACGG |
|  | R primer (5′-3′) | GCAGACCTGTGAGGTGTTCC |
| **AC004837.2** | F primer (5′-3′) | CCTCTGTTTCAAGTCTCTGGCT |
|  | R primer (5′-3′) | GCATTAGAAAGCAATACTGGGAGT |
| **AL078581.2** | F primer (5′-3′) | AAGACCAAGGGCACAGTCTAA |
|  | R primer (5′-3′) | ATGGCTGAGATGCAGGAGTAG |
