# Supplementary material for: Construction and Validation of a Novel Cuproptosis-Related Seven-lncRNA Signature to Predict the Outcomes, Immunotherapeutic Responses, and Targeted Therapy in Patients with Clear Cell Renal Cell Carcinoma
Source: Dis Markers. 2023 Jan 25;2023:7219794. doi: 10.1155/2023/7219794 (PMC9893525; doi:10.1155/2023/7219794)
Supplement: Supplementary 3 — Figure S1: K-M curves exhibited the association between 10 differentially expressed genes and PFS of KIRC survival. (A) ATP7A; (B) DBT; (C) DLAT; (D) DLD; (E) DLST; (F) FDX1; (G) MTF1; (H) NFE2L2; (I) PDHB; (J) SLC31A1. [file 7219794.f3.pdf]

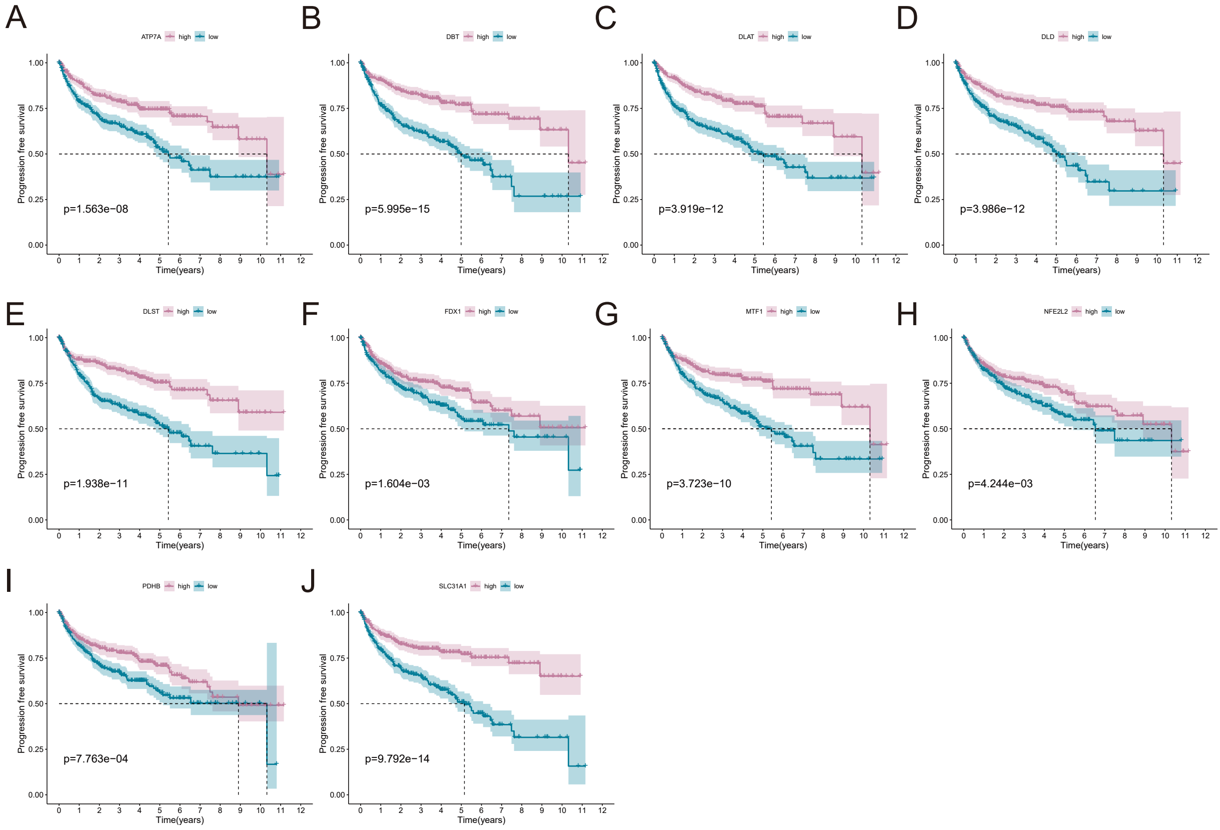

Fig. S1 K-M cuveces exhibited the association between 10 differentially expressed genes and PFS of KIRC survival.

A. ATP7A B. DBT C. DLAT D. DLD E. DLST F. FDX1 G. MTF1 H. NFE2L2 I. PDHB J. SLC31A1
