# Supplementary figures and images for: Construction and Validation of a Novel Cuproptosis-Related Seven-lncRNA Signature to Predict the Outcomes, Immunotherapeutic Responses, and Targeted Therapy in Patients with Clear Cell Renal Cell Carcinoma
Source: Dis Markers. 2023 Jan 25;2023:7219794. doi: 10.1155/2023/7219794 (PMC9893525; doi:10.1155/2023/7219794)

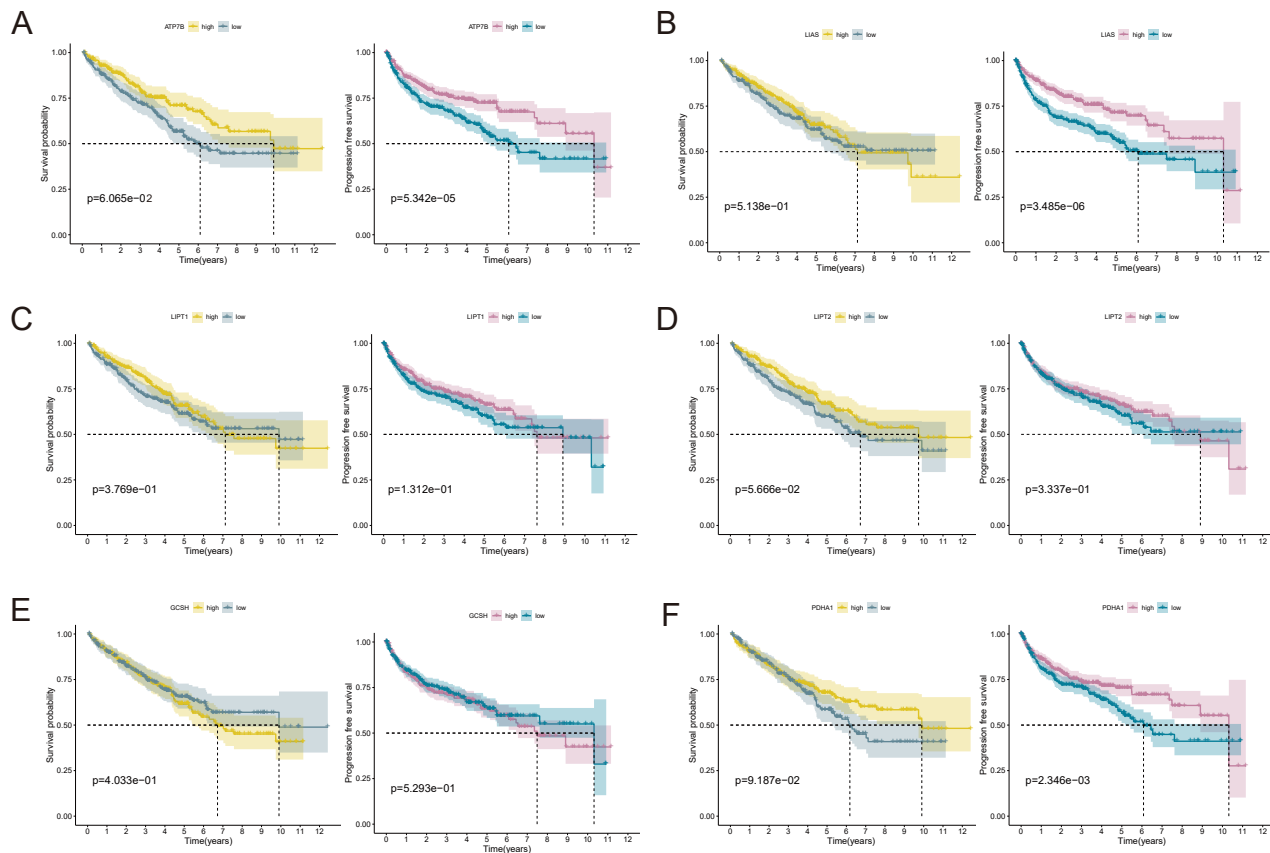

Fig. S2 The K-M curves of six genes.

A. ATP7B B. LIAS C. LIPT1 D. LIPT2 E. GCSH F. PDHA1

Supplement: Supplementary 4 — Figure S2: K-M curves of six genes. (A) ATP7B; (B) LIAS; (C) LIPT1; (D) LIPT2; (E) GCSH; (F) PDHA1. [file 7219794.f4.pdf]
