# Supplementary material for: Construction and Validation of a Novel Cuproptosis-Related Seven-lncRNA Signature to Predict the Outcomes, Immunotherapeutic Responses, and Targeted Therapy in Patients with Clear Cell Renal Cell Carcinoma
Source: Dis Markers. 2023 Jan 25;2023:7219794. doi: 10.1155/2023/7219794 (PMC9893525; doi:10.1155/2023/7219794)
Supplement: Supplementary 5 — Figure S3: a Sankey diagram showed the connection between the cuproptosis related lncRNAs and cuproptosis-related genes. Supplementary 5. Figure S4: forest plots exhibited the results of the Cox regression analyses of the 535 cuproptosis-related seven-lncRNA signature. (A, B) The univariate Cox regression analysis and multivariate Cox regression analysis in the testing set. (C, D) The univariate Cox regression analysis and multivariate Cox regression analysis in the entire set. [file 7219794.f5.pdf]

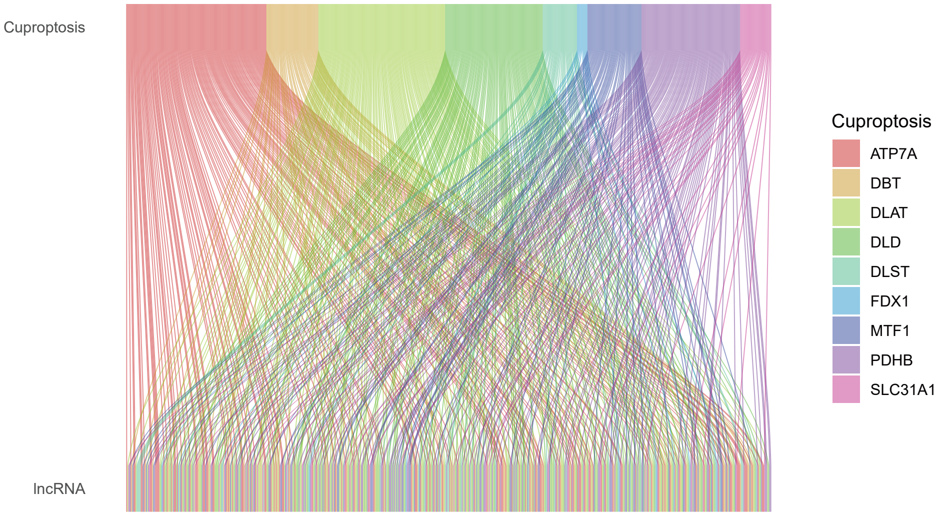

Fig. S3 A sankey diagram showed the connection between the cuproptosis-related lncRNAs and cuproptosis-related genes.

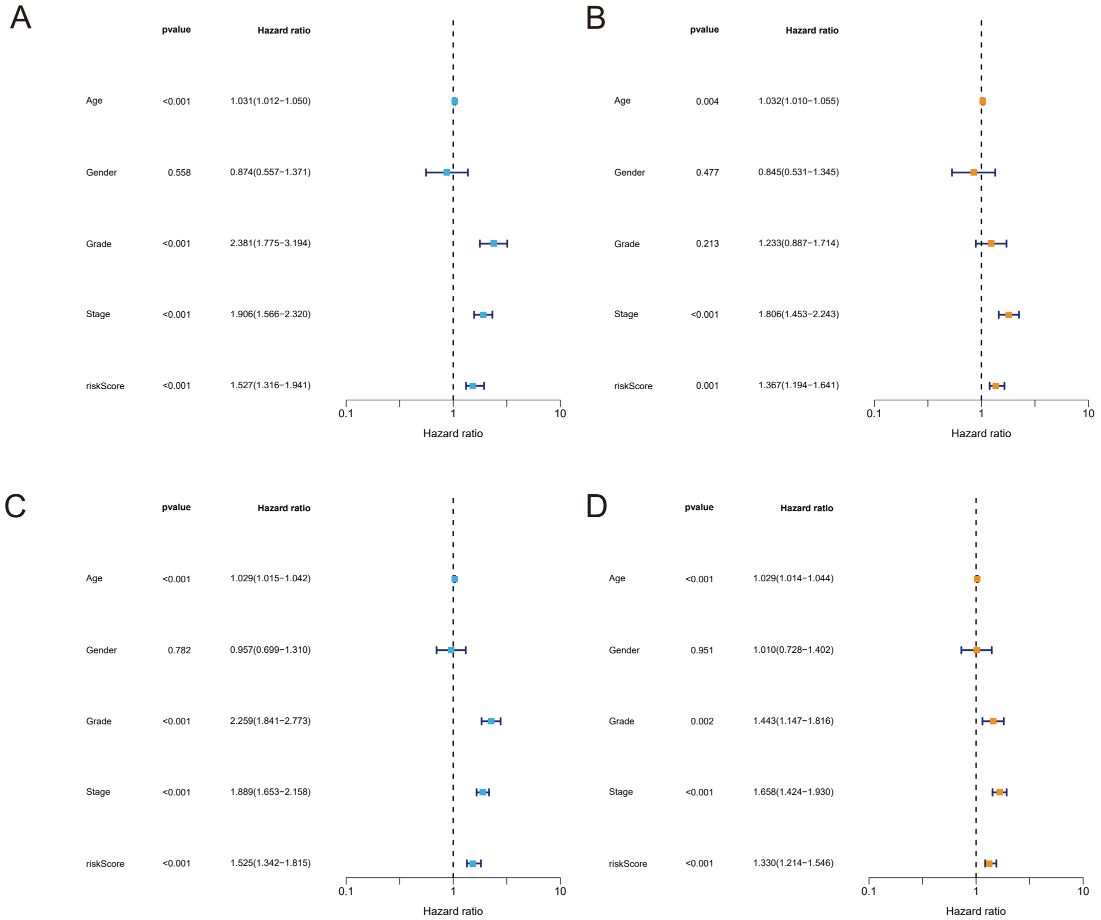

Fig.S4 Forest plots exhibited the results of the Cox regression analyses of the cuproptosis-related seven-lncRNA signature.
A, B The univariate Cox regression analysis and multivariate Cox regression analysis in the testing set. C, D The univariate Cox regression analysis and multivariate Cox regression analysis in the entire set.
