# Supplementary material for: Construction and Validation of a Novel Cuproptosis-Related Seven-lncRNA Signature to Predict the Outcomes, Immunotherapeutic Responses, and Targeted Therapy in Patients with Clear Cell Renal Cell Carcinoma
Source: Dis Markers. 2023 Jan 25;2023:7219794. doi: 10.1155/2023/7219794 (PMC9893525; doi:10.1155/2023/7219794)
Supplement: Supplementary 6 — Figure S5: K-M curves exhibited the relationship between the expression levels of seven candidate lncRNAs and the PFS of KIRC patients. (A) LINC02154; (B) MINRC; (C) FOXD2-AS1; (D) AC004837.2; (E) SMARCA5-AS1; (F) LINC01671; (G) AL078581.2. [file 7219794.f6.pdf]

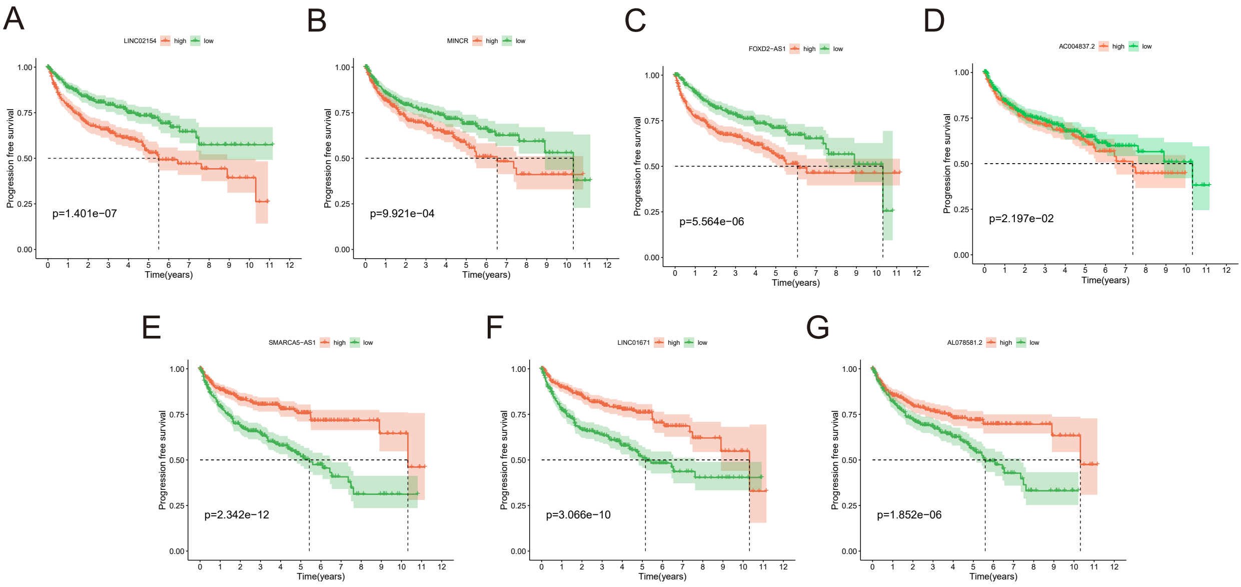

Fig. S5 The K-M curves exhibited the relationship between the epression levels of seven candidate lncRNAs and the PFS of KIRC patients.

A LINC02154 B MINRC C FOXD2-AS1 D AC004837.2 E SMARCA5-AS1 F LINC01671 G AL078581.2
