# Supplementary material for: Construction and Validation of a Novel Cuproptosis-Related Seven-lncRNA Signature to Predict the Outcomes, Immunotherapeutic Responses, and Targeted Therapy in Patients with Clear Cell Renal Cell Carcinoma
Source: Dis Markers. 2023 Jan 25;2023:7219794. doi: 10.1155/2023/7219794 (PMC9893525; doi:10.1155/2023/7219794)
Supplement: Supplementary 8 — Table S3: the lncRNAs, miRNAs, and mRNAs used for construction for ceRNA network. [file 7219794.f8.docx]

| Node1 | Node2 | Line |
| --- | --- | --- |
| WDFY3-AS2 | hsa-miR-507 | lncRNA |
| WDFY3-AS2 | hsa-miR-135a-5p | lncRNA |
| WDFY3-AS2 | hsa-miR-139-5p | lncRNA |
| WDFY3-AS2 | hsa-miR-142-3p | lncRNA |
| WDFY3-AS2 | hsa-miR-4458 | lncRNA |
| WDFY3-AS2 | hsa-miR-4500 | lncRNA |
| WDFY3-AS2 | hsa-miR-4735-3p | lncRNA |
| WDFY3-AS2 | hsa-miR-206 | lncRNA |
| WDFY3-AS2 | hsa-miR-613 | lncRNA |
| WDFY3-AS2 | hsa-miR-429 | lncRNA |
| WDFY3-AS2 | hsa-miR-590-5p | lncRNA |
| WDFY3-AS2 | hsa-miR-217 | lncRNA |
| WDFY3-AS2 | hsa-miR-23b-3p | lncRNA |
| WDFY3-AS2 | hsa-miR-24-3p | lncRNA |
| WDFY3-AS2 | hsa-miR-1297 | lncRNA |
| WDFY3-AS2 | hsa-miR-4465 | lncRNA |
| WDFY3-AS2 | hsa-miR-27a-3p | lncRNA |
| WDFY3-AS2 | hsa-miR-107 | lncRNA |
| WDFY3-AS2 | hsa-miR-338-3p | lncRNA |
| WDFY3-AS2 | hsa-miR-33a-3p | lncRNA |
| WDFY3-AS2 | hsa-miR-449c-5p | lncRNA |
| WDFY3-AS2 | hsa-miR-375 | lncRNA |
| WDFY3-AS2 | hsa-miR-425-5p | lncRNA |
| WDFY3-AS2 | hsa-miR-455-5p | lncRNA |
| WDFY3-AS2 | hsa-miR-129-5p | lncRNA |
| WDFY3-AS2 | hsa-miR-490-3p | lncRNA |
| EMX2OS | hsa-miR-4770 | lncRNA |
| EMX2OS | hsa-miR-184 | lncRNA |
| EMX2OS | hsa-miR-22-3p | lncRNA |
| EMX2OS | hsa-miR-24-3p | lncRNA |
| EMX2OS | hsa-miR-449c-5p | lncRNA |
| EMX2OS | hsa-miR-455-5p | lncRNA |
| SNHG15 | hsa-miR-4735-3p | lncRNA |
| SNHG15 | hsa-miR-338-3p | lncRNA |
| SNHG15 | hsa-miR-490-3p | lncRNA |
| SMARCA5-AS1 | hsa-miR-137 | lncRNA |
| SMARCA5-AS1 | hsa-miR-4770 | lncRNA |
| SMARCA5-AS1 | hsa-miR-4458 | lncRNA |
| SMARCA5-AS1 | hsa-miR-4500 | lncRNA |
| SMARCA5-AS1 | hsa-miR-24-3p | lncRNA |
| AC104758.1 | hsa-miR-490-3p | lncRNA |
| hsa-miR-4458 | SOCS1 | mRNA |
| hsa-miR-613 | PABPC1L2A | mRNA |
| hsa-miR-449c-5p | C4orf45 | mRNA |
| hsa-miR-135a-5p | YBX2 | mRNA |
| hsa-miR-449c-5p | TRIM74 | mRNA |
| hsa-miR-590-5p | CLIC5 | mRNA |
| hsa-miR-33a-3p | KLHL11 | mRNA |
| hsa-miR-33a-3p | SOX21 | mRNA |
| hsa-miR-429 | ZNF366 | mRNA |
| hsa-miR-24-3p | NOS1 | mRNA |
| hsa-miR-107 | SYT10 | mRNA |
| hsa-miR-24-3p | NOTUM | mRNA |
| hsa-miR-33a-3p | GOLGA8B | mRNA |
| hsa-miR-613 | PI15 | mRNA |
| hsa-miR-33a-3p | GPR78 | mRNA |
| hsa-miR-1297 | SLC5A8 | mRNA |
| hsa-miR-33a-3p | BRINP2 | mRNA |
| hsa-miR-206 | LIPI | mRNA |
| hsa-miR-33a-3p | PRG4 | mRNA |
| hsa-miR-338-3p | PLG | mRNA |
| hsa-miR-338-3p | PLXNB3 | mRNA |
| hsa-miR-129-5p | TNFSF14 | mRNA |
| hsa-miR-507 | NOS1 | mRNA |
| hsa-miR-137 | CHRM3 | mRNA |
| hsa-miR-1297 | TMPRSS11F | mRNA |
| hsa-miR-142-3p | RAPH1 | mRNA |
| hsa-miR-139-5p | PCDHGA9 | mRNA |
| hsa-miR-449c-5p | CNTN3 | mRNA |
| hsa-miR-129-5p | KLF17 | mRNA |
| hsa-miR-429 | CALN1 | mRNA |
| hsa-miR-613 | PAX3 | mRNA |
| hsa-miR-490-3p | TRIM72 | mRNA |
| hsa-miR-129-5p | DGKI | mRNA |
| hsa-miR-613 | ZBTB20 | mRNA |
| hsa-miR-490-3p | CCDC103 | mRNA |
| hsa-miR-449c-5p | FAM47E-STBD1 | mRNA |
| hsa-miR-206 | GMNC | mRNA |
| hsa-miR-129-5p | FKBP2 | mRNA |
| hsa-miR-33a-3p | LCE1A | mRNA |
| hsa-miR-33a-3p | RAC3 | mRNA |
| hsa-miR-425-5p | CXCL3 | mRNA |
| hsa-miR-24-3p | REEP2 | mRNA |
| hsa-miR-425-5p | KRTAP4-1 | mRNA |
| hsa-miR-27a-3p | MAPK8IP3 | mRNA |
| hsa-miR-507 | LHX8 | mRNA |
| hsa-miR-206 | COL19A1 | mRNA |
| hsa-miR-206 | SHPRH | mRNA |
| hsa-miR-4500 | CPA4 | mRNA |
| hsa-miR-4465 | CHAC1 | mRNA |
| hsa-miR-507 | CYP2C9 | mRNA |
| hsa-miR-429 | BTBD18 | mRNA |
| hsa-miR-429 | CASR | mRNA |
| hsa-miR-22-3p | PLCXD3 | mRNA |
| hsa-miR-507 | PARP15 | mRNA |
| hsa-miR-1297 | GMNC | mRNA |
| hsa-miR-1297 | NOS1 | mRNA |
| hsa-miR-490-3p | LTB4R | mRNA |
| hsa-miR-338-3p | ZBTB20 | mRNA |
| hsa-miR-107 | PABPC1L | mRNA |
| hsa-miR-206 | SPEG | mRNA |
| hsa-miR-23b-3p | TRIM63 | mRNA |
| hsa-miR-33a-3p | FREM2 | mRNA |
| hsa-miR-425-5p | IL11 | mRNA |
| hsa-miR-142-3p | PATE1 | mRNA |
| hsa-miR-613 | LIX1 | mRNA |
| hsa-miR-590-5p | CALN1 | mRNA |
| hsa-miR-449c-5p | STMN2 | mRNA |
| hsa-miR-4735-3p | F3 | mRNA |
| hsa-miR-33a-3p | FRMD7 | mRNA |
| hsa-miR-129-5p | SALL4 | mRNA |
| hsa-miR-429 | MXD3 | mRNA |
| hsa-miR-507 | AK5 | mRNA |
| hsa-miR-613 | PABPC1L2B | mRNA |
| hsa-miR-490-3p | MTRNR2L1 | mRNA |
| hsa-miR-4770 | AIRE | mRNA |
| hsa-miR-425-5p | FST | mRNA |
| hsa-miR-613 | MTRNR2L3 | mRNA |
| hsa-miR-24-3p | KCNIP1 | mRNA |
| hsa-miR-449c-5p | CALN1 | mRNA |
| hsa-miR-1297 | DMRT3 | mRNA |
| hsa-miR-490-3p | GALNTL6 | mRNA |
| hsa-miR-27a-3p | NIPAL4 | mRNA |
| hsa-miR-449c-5p | FBXO40 | mRNA |
| hsa-miR-129-5p | SAPCD1 | mRNA |
| hsa-miR-33a-3p | PI15 | mRNA |
| hsa-miR-613 | COL19A1 | mRNA |
| hsa-miR-429 | OCLN | mRNA |
| hsa-miR-24-3p | CDKN2A | mRNA |
| hsa-miR-24-3p | ADAMTS14 | mRNA |
| hsa-miR-22-3p | CIDEC | mRNA |
| hsa-miR-206 | FAM83A | mRNA |
| hsa-miR-507 | UNC13C | mRNA |
| hsa-miR-338-3p | PRKCG | mRNA |
| hsa-miR-24-3p | CRYGN | mRNA |
| hsa-miR-107 | LRP2 | mRNA |
| hsa-miR-590-5p | ZNF728 | mRNA |
| hsa-miR-429 | BASP1 | mRNA |
| hsa-miR-184 | FOSB | mRNA |
| hsa-miR-613 | SPEG | mRNA |
| hsa-miR-24-3p | GAD1 | mRNA |
| hsa-miR-107 | KCNIP1 | mRNA |
| hsa-miR-137 | CALN1 | mRNA |
| hsa-miR-1297 | SYT10 | mRNA |
| hsa-miR-490-3p | RTBDN | mRNA |
| hsa-miR-129-5p | PARP15 | mRNA |
| hsa-miR-429 | GABRB3 | mRNA |
| hsa-miR-507 | PRRT2 | mRNA |
| hsa-miR-206 | LIX1 | mRNA |
| hsa-miR-107 | PLEKHS1 | mRNA |
| hsa-miR-206 | PABPC1L2B | mRNA |
| hsa-miR-33a-3p | CYP1A2 | mRNA |
| hsa-miR-107 | ARHGAP5 | mRNA |
| hsa-miR-590-5p | ZNF460 | mRNA |
| hsa-miR-24-3p | KRTAP5-9 | mRNA |
| hsa-miR-449c-5p | OLFM4 | mRNA |
| hsa-miR-206 | MTRNR2L3 | mRNA |
| hsa-miR-24-3p | KIF18B | mRNA |
| hsa-miR-429 | SHOX2 | mRNA |
| hsa-miR-507 | FLRT3 | mRNA |
| hsa-miR-375 | KCNN4 | mRNA |
| hsa-miR-206 | FOSB | mRNA |
| hsa-miR-338-3p | GPRC5A | mRNA |
| hsa-miR-107 | KIF18B | mRNA |
| hsa-miR-23b-3p | FOSB | mRNA |
| hsa-miR-24-3p | KLHL11 | mRNA |
| hsa-miR-27a-3p | GRM5 | mRNA |
| hsa-miR-33a-3p | PABPC1L2A | mRNA |
| hsa-miR-129-5p | LUC7L | mRNA |
| hsa-miR-33a-3p | KCNV1 | mRNA |
| hsa-miR-129-5p | SYT16 | mRNA |
| hsa-miR-455-5p | PI15 | mRNA |
| hsa-miR-27a-3p | LHX8 | mRNA |
| hsa-miR-206 | PABPC1L2A | mRNA |
| hsa-miR-107 | KCNN4 | mRNA |
| hsa-miR-24-3p | PRRT2 | mRNA |
| hsa-miR-507 | ZBTB20 | mRNA |
| hsa-miR-429 | SOX2 | mRNA |
| hsa-miR-139-5p | GMNC | mRNA |
| hsa-miR-33a-3p | IGFN1 | mRNA |
| hsa-miR-507 | MCIDAS | mRNA |
| hsa-miR-184 | CLIC5 | mRNA |
| hsa-miR-490-3p | KRTAP24-1 | mRNA |
| hsa-miR-24-3p | RCN3 | mRNA |
| hsa-miR-33a-3p | NFKBIZ | mRNA |
| hsa-miR-135a-5p | PLCXD3 | mRNA |
| hsa-miR-33a-3p | ARHGAP5 | mRNA |
| hsa-miR-33a-3p | EDAR | mRNA |
| hsa-miR-33a-3p | PITX2 | mRNA |
| hsa-miR-1297 | CHAC1 | mRNA |
| hsa-miR-206 | PI15 | mRNA |
| hsa-miR-490-3p | ARHGAP33 | mRNA |
| hsa-miR-33a-3p | ITIH2 | mRNA |
| hsa-miR-129-5p | COL19A1 | mRNA |
| hsa-miR-27a-3p | MFSD2A | mRNA |
| hsa-miR-429 | BPY2C | mRNA |
| hsa-miR-455-5p | STRA6 | mRNA |
| hsa-miR-129-5p | ZBTB20 | mRNA |
| hsa-miR-27a-3p | UNC13C | mRNA |
| hsa-miR-139-5p | GOLGA8B | mRNA |
| hsa-miR-449c-5p | CXCL1 | mRNA |
| hsa-miR-129-5p | TRIM49C | mRNA |
| hsa-miR-33a-3p | LHX8 | mRNA |
| hsa-miR-507 | PI15 | mRNA |
| hsa-miR-613 | LIPI | mRNA |
| hsa-miR-507 | RAPH1 | mRNA |
| hsa-miR-507 | MLLT11 | mRNA |
| hsa-miR-27a-3p | DMRT3 | mRNA |
| hsa-miR-425-5p | PABPN1 | mRNA |
| hsa-miR-139-5p | TMEM132D | mRNA |
| hsa-miR-129-5p | ENTHD1 | mRNA |
| hsa-miR-206 | ZBTB20 | mRNA |
| hsa-miR-129-5p | PITX1 | mRNA |
| hsa-miR-27a-3p | EDAR | mRNA |
| hsa-miR-23b-3p | MAB21L2 | mRNA |
| hsa-miR-425-5p | LIX1 | mRNA |
| hsa-miR-206 | PAX3 | mRNA |
| hsa-miR-429 | TUBB3 | mRNA |
| hsa-miR-613 | GMNC | mRNA |
| hsa-miR-375 | SOX2 | mRNA |
| hsa-miR-429 | SMPX | mRNA |
| hsa-miR-507 | PABPC1L | mRNA |
| hsa-miR-27a-3p | GABRB3 | mRNA |
| hsa-miR-1297 | NOL4 | mRNA |
| hsa-miR-33a-3p | OLIG1 | mRNA |
| hsa-miR-1297 | CCNL2 | mRNA |
| hsa-miR-449c-5p | IGFBP1 | mRNA |
| hsa-miR-1297 | SLC12A1 | mRNA |
| hsa-miR-455-5p | BAAT | mRNA |
| hsa-miR-490-3p | LUC7L | mRNA |
| hsa-miR-24-3p | FST | mRNA |
| hsa-miR-590-5p | CCL20 | mRNA |
| hsa-miR-507 | AGAP9 | mRNA |
| hsa-miR-24-3p | ATP2A1 | mRNA |
| hsa-miR-27a-3p | ZBTB20 | mRNA |
| hsa-miR-33a-3p | SHPRH | mRNA |
| hsa-miR-33a-3p | LRRTM2 | mRNA |
| hsa-miR-27a-3p | ASIC1 | mRNA |
| hsa-miR-24-3p | GABRB3 | mRNA |
| hsa-miR-137 | CIDEC | mRNA |
| hsa-miR-425-5p | CLSTN2 | mRNA |
| hsa-miR-455-5p | ZPBP2 | mRNA |
| hsa-miR-425-5p | CENPT | mRNA |
| hsa-miR-4458 | CPA4 | mRNA |
| hsa-miR-1297 | CLVS2 | mRNA |
| hsa-miR-27a-3p | GLRA2 | mRNA |
| hsa-miR-139-5p | SLC25A21 | mRNA |
| hsa-miR-33a-3p | SLC4A10 | mRNA |
| hsa-miR-107 | IFIT1B | mRNA |
| hsa-miR-33a-3p | GOLGA8A | mRNA |
| hsa-miR-455-5p | F3 | mRNA |
| hsa-miR-1297 | CLIC5 | mRNA |
| hsa-miR-129-5p | ARHGAP5 | mRNA |
| hsa-miR-24-3p | FREM2 | mRNA |
| hsa-miR-507 | LRRC55 | mRNA |
| hsa-miR-507 | FCRL2 | mRNA |
| hsa-miR-129-5p | GRHL3 | mRNA |
| hsa-miR-33a-3p | ZBTB20 | mRNA |
| hsa-miR-1297 | ZBTB20 | mRNA |
| hsa-miR-27a-3p | STMN2 | mRNA |
| hsa-miR-217 | ZBTB20 | mRNA |
| hsa-miR-33a-3p | MAB21L2 | mRNA |
| hsa-miR-425-5p | SERPINE1 | mRNA |
| hsa-miR-33a-3p | PARP15 | mRNA |
| hsa-miR-107 | GOLGA8B | mRNA |
| hsa-miR-217 | SFTPB | mRNA |
| hsa-miR-613 | STMN2 | mRNA |
| hsa-miR-33a-3p | AQP9 | mRNA |
| hsa-miR-33a-3p | CUBN | mRNA |
| hsa-miR-33a-3p | EML5 | mRNA |
| hsa-miR-129-5p | FCRL2 | mRNA |
| hsa-miR-142-3p | ZNF460 | mRNA |
| hsa-miR-455-5p | LRP2 | mRNA |
| hsa-miR-23b-3p | NOL4 | mRNA |
| hsa-miR-27a-3p | F3 | mRNA |
| hsa-miR-429 | CCDC144A | mRNA |
| hsa-miR-23b-3p | GRM5 | mRNA |
| hsa-miR-1297 | HPX | mRNA |
| hsa-miR-27a-3p | FOSB | mRNA |
| hsa-miR-135a-5p | HCN2 | mRNA |
| hsa-miR-129-5p | KCNV1 | mRNA |
| hsa-miR-455-5p | GNRH1 | mRNA |
| hsa-miR-33a-3p | STX1A | mRNA |
| hsa-miR-27a-3p | KCNJ1 | mRNA |
| hsa-miR-184 | USH1G | mRNA |
| hsa-miR-129-5p | NOL4 | mRNA |
| hsa-miR-4500 | SOCS1 | mRNA |
| hsa-miR-27a-3p | MMP13 | mRNA |
| hsa-miR-24-3p | PLEKHS1 | mRNA |
| hsa-miR-24-3p | LTB4R | mRNA |
| hsa-miR-27a-3p | CA10 | mRNA |
| hsa-miR-142-3p | FLNC | mRNA |
| hsa-miR-107 | SALL4 | mRNA |
| hsa-miR-27a-3p | CLSTN2 | mRNA |
| hsa-miR-129-5p | GRM5 | mRNA |
| hsa-miR-33a-3p | AK5 | mRNA |
| hsa-miR-24-3p | LRRTM2 | mRNA |
| hsa-miR-107 | NOS1 | mRNA |
| hsa-miR-206 | STMN2 | mRNA |
| hsa-miR-507 | FST | mRNA |
| hsa-miR-507 | CDKN2A | mRNA |
| hsa-miR-27a-3p | HRK | mRNA |
| hsa-miR-490-3p | FLRT3 | mRNA |
| hsa-miR-449c-5p | DGKI | mRNA |
| hsa-miR-135a-5p | CLVS2 | mRNA |
| hsa-miR-22-3p | HTR3A | mRNA |
| hsa-miR-22-3p | TACR3 | mRNA |
| hsa-miR-135a-5p | CALN1 | mRNA |
| hsa-miR-142-3p | ZBTB20 | mRNA |
| hsa-miR-1297 | ANKRD63 | mRNA |
| hsa-miR-1297 | COL19A1 | mRNA |
| hsa-miR-24-3p | SOST | mRNA |
| hsa-miR-129-5p | LRRC55 | mRNA |
| hsa-miR-24-3p | RHPN1 | mRNA |
| hsa-miR-129-5p | CNGB1 | mRNA |
| hsa-miR-137 | NBEAL1 | mRNA |
| hsa-miR-429 | GPR173 | mRNA |
| hsa-miR-24-3p | KLF17 | mRNA |
| hsa-miR-107 | VAMP1 | mRNA |
| hsa-miR-1297 | PON1 | mRNA |
| hsa-miR-129-5p | GNRHR | mRNA |
| hsa-miR-590-5p | PITX2 | mRNA |
| hsa-miR-129-5p | TRIM72 | mRNA |
| hsa-miR-107 | GLRA2 | mRNA |
| hsa-miR-27a-3p | GFPT2 | mRNA |
| hsa-miR-455-5p | ZNF460 | mRNA |
| hsa-miR-107 | IGSF23 | mRNA |
| hsa-miR-129-5p | CLDN19 | mRNA |
| hsa-miR-22-3p | MAPK8IP3 | mRNA |
| hsa-miR-338-3p | PPP1R1A | mRNA |
| hsa-miR-107 | LUC7L | mRNA |
| hsa-miR-22-3p | GRM5 | mRNA |
| hsa-miR-27a-3p | FAM193B | mRNA |
| hsa-miR-206 | TMEM132D | mRNA |
| hsa-miR-33a-3p | HTR1E | mRNA |
| hsa-miR-429 | NOL12 | mRNA |
| hsa-miR-429 | CLVS2 | mRNA |
| hsa-miR-1297 | CILP | mRNA |
| hsa-miR-129-5p | NTNG2 | mRNA |
| hsa-miR-449c-5p | RIMBP2 | mRNA |
| hsa-miR-338-3p | HJURP | mRNA |
| hsa-miR-590-5p | KLK2 | mRNA |
| hsa-miR-449c-5p | IFIT1B | mRNA |
| hsa-miR-23b-3p | EGR1 | mRNA |
| hsa-miR-139-5p | LRP2 | mRNA |
| hsa-miR-206 | PLEKHS1 | mRNA |
| hsa-miR-449c-5p | ANKRD63 | mRNA |
| hsa-miR-449c-5p | CLIC5 | mRNA |
| hsa-miR-129-5p | COL1A1 | mRNA |
| hsa-miR-33a-3p | PASD1 | mRNA |
| hsa-miR-507 | LRP2 | mRNA |
| hsa-miR-107 | ASIC1 | mRNA |
| hsa-miR-338-3p | TMEM74B | mRNA |
| hsa-miR-23b-3p | PLCXD3 | mRNA |
| hsa-miR-129-5p | IGF2BP2 | mRNA |
| hsa-miR-129-5p | VAMP1 | mRNA |
| hsa-miR-338-3p | ADAMTS14 | mRNA |
| hsa-miR-338-3p | KCNV1 | mRNA |
| hsa-miR-24-3p | FRMD7 | mRNA |
| hsa-miR-107 | GPRC5A | mRNA |
| hsa-miR-129-5p | NPHS2 | mRNA |
| hsa-miR-490-3p | HAVCR1 | mRNA |
| hsa-miR-507 | TRIM63 | mRNA |
| hsa-miR-1297 | PLCXD3 | mRNA |
| hsa-miR-490-3p | TRIM63 | mRNA |
| hsa-miR-507 | SOX2 | mRNA |
| hsa-miR-429 | ZBTB20 | mRNA |
| hsa-miR-449c-5p | CELSR3 | mRNA |
| hsa-miR-338-3p | CHRNA4 | mRNA |
| hsa-miR-137 | SERPINA3 | mRNA |
| hsa-miR-33a-3p | RNF207 | mRNA |
| hsa-miR-129-5p | FST | mRNA |
| hsa-miR-33a-3p | SLC25A21 | mRNA |
| hsa-miR-338-3p | RAPH1 | mRNA |
| hsa-miR-1297 | GRHL3 | mRNA |
| hsa-miR-338-3p | PI15 | mRNA |
| hsa-miR-27a-3p | FLRT3 | mRNA |
| hsa-miR-338-3p | NOL4 | mRNA |
| hsa-miR-429 | ZNF692 | mRNA |
| hsa-miR-425-5p | GAD1 | mRNA |
| hsa-miR-107 | PRRT2 | mRNA |
| hsa-miR-429 | PPP2R2C | mRNA |
| hsa-miR-429 | PI15 | mRNA |
| hsa-miR-129-5p | PLCXD3 | mRNA |
| hsa-miR-22-3p | ZNF460 | mRNA |
| hsa-miR-33a-3p | BASP1 | mRNA |
| hsa-miR-338-3p | PTK6 | mRNA |
| hsa-miR-139-5p | ZBTB20 | mRNA |
| hsa-miR-375 | F3 | mRNA |
