# Supplementary material for: Construction and Validation of a Novel Cuproptosis-Related Seven-lncRNA Signature to Predict the Outcomes, Immunotherapeutic Responses, and Targeted Therapy in Patients with Clear Cell Renal Cell Carcinoma
Source: Dis Markers. 2023 Jan 25;2023:7219794. doi: 10.1155/2023/7219794 (PMC9893525; doi:10.1155/2023/7219794)
Supplement: Supplementary 9 — Table S4: biological functions of the 208 target mRNAs explored by the online tool Metascape. [file 7219794.f9.pdf]

MyList	Gene ID	Type	Tax ID	Homologen	Homologen	Gene Symb	Description	Biological P	Kinase Clas	Protein Fun	Subcellular	Drug (DrugI	Canonical P	Hallmark G	GO:009953	GO:003000	GO:006106	hsa04080 N	GO:190169	M5885 NAE	WP1533 Vi	GO:004887	GO:000682	GO:004326	GO:004247	GO:005080	GO:005118	GO:009874	R-HSA-679	hsa03015 n	GO:004356	GO:000761	GO:004217	GO:0032963 collagen metabolic process
FST	10468	symbol	H. sapiens	10468	H. sapiens	FST	follistatin	GO:0043616 keratinocy		Predicted ir	Vesicles (Ap	D-Myo-Inos	(M181)PID I	(M5909)HA	0	0	0	0	0	1	0	0	0	0	1	0	1	0	0	0	0	0	0	1
NOL12	79159	symbol	H. sapiens	79159	H. sapiens	NOL12	nucleolar protein 12			Predicted ir	Mitotic chromosome;Nucleoli (Approved); Addi				0	0	0	0	0	0	0	0	0	0	0	0	0	0	0	0	0	0	0	0
RAPH1	65059	symbol	H. sapiens	65059	H. sapiens	RAPH1	Ras associat	GO:0048675 axon exter		Predicted ir	Nuclear bodies;Plasma membrane (Approved);				0	0	0	0	0	0	0	0	0	0	0	0	0	0	0	0	0	0	0	0
HAVCR1	26762	symbol	H. sapiens	26762	H. sapiens	HAVCR1	hepatitis A \	GO:0033005 positive re		Predicted ir	Vesicles (Approved)				0	0	0	0	0	0	0	0	0	0	0	0	0	0	0	0	0	0	0	0
KCNJ1	3758	symbol	H. sapiens	3758	H. sapiens	KCNJ1	potassium i	GO:1990573 potassium		FDA approved drug targ		Bethanidine; Glimepiride; Minoxidil			1	1	0	0	0	0	0	0	0	1	0	0	0	0	0	0	0	0	0	0
SMPX	23676	symbol	H. sapiens	23676	H. sapiens	SMPX	small muscl	GO:0006941 striated m		Disease rela	Plasma membrane (Approved)			(M5956)HA	0	0	0	0	0	0	0	0	0	0	0	0	0	0	0	0	0	0	0	0
SOST	50964	symbol	H. sapiens	50964	H. sapiens	SOST	sclerostin	GO:0071374 cellular re:		Human disease related		Romosozumab			0	0	0	0	0	0	0	0	0	0	0	0	0	0	0	0	0	0	0	0
FREM2	341640	symbol	H. sapiens	341640	H. sapiens	FREM2	FRAS1 relat	GO:0042733 embryonic		Disease rela	Cytosol (Approved)		(M5880)NABA ECM AFF		0	0	0	0	0	1	0	0	0	0	0	0	0	0	0	0	0	0	0	0
CYP2C9	1559	symbol	H. sapiens	1559	H. sapiens	CYP2C9	cytochrome	GO:0016098 monoterp		ENZYME pr	Vesicles (Ur	Ferroheme C; Diacerein; Benzbrom			0	0	0	0	1	0	0	0	0	0	0	0	0	0	0	0	0	0	1	0
FKBP2	2286	symbol	H. sapiens	2286	H. sapiens	FKBP2	FKBP prolyl	GO:0000413 protein pe		ENZYME pr	Plasma membrane (Approved)			(M5924)HA	0	0	0	0	0	0	0	0	0	0	0	0	0	0	0	0	0	0	0	0
MAB21L2	10586	symbol	H. sapiens	10586	H. sapiens	MAB21L2	mab-21 like	GO:0010172 embryonic		Disease rela	Nucleoplasm (Enhanced)				0	0	0	0	0	0	0	0	0	0	0	0	0	0	0	0	0	0	0	0
CHRM3	1131	symbol	H. sapiens	1131	H. sapiens	CHRM3	cholinergic	GO:0046541 saliva secr		Disease rela	Plasma mer	Cevimeline; Tramadol; Succinylchol			1	0	0	1	1	0	1	0	0	1	0	0	0	0	1	0	0	0	0	0
AQP9	366	symbol	H. sapiens	366	H. sapiens	AQP9	aquaporin 9	GO:0015722 canalicular		Transporters:Transporter channels and pores				(M5948)HA	0	0	0	0	1	0	0	0	1	0	0	0	0	0	1	0	0	0	0	0
KLK2	3817	symbol	H. sapiens	3817	H. sapiens	KLK2	kallikrein re	GO:0031638 zymogen a		Peptidases:Serine-type peptidases;			(M151)PID /	(M5908)HA	0	0	0	0	0	0	1	0	0	0	0	0	0	0	0	0	0	0	0	1
CA10	56934	symbol	H. sapiens	56934	H. sapiens	CA10	carbonic an	GO:0006730 one-carbo		Predicted ir	Vesicles (Ap	Zonisamide			0	0	0	0	0	0	0	0	0	0	0	0	0	0	0	0	0	0	0	0
FBXO40	51725	symbol	H. sapiens	51725	H. sapiens	FBXO40	F-box prote	GO:0042692 muscle cel		Predicted intracellular proteins					0	0	1	0	0	0	0	0	0	0	0	0	0	0	0	0	0	0	0	0
IL11	3589	symbol	H. sapiens	3589	H. sapiens	IL11	interleukin	GO:0030219 megakaryo		Predicted intracellular proteins; Pre			(M5883)NA	(M5950)HA	0	0	0	0	0	1	0	0	0	0	0	0	0	0	0	0	0	0	1	0
CNGB1	1258	symbol	H. sapiens	1258	H. sapiens	CNGB1	cyclic nucle	GO:0099105 ion channe		Human dise	Cytosol (Approved); Additional: Vesicles				1	1	0	0	0	0	0	1	0	0	0	0	0	0	0	0	0	0	0	0
BPY2C	442868	symbol	H. sapiens	442868	H. sapiens	BPY2C	basic charge	GO:0007338 single ferti		Predicted intracellular proteins					0	0	0	0	0	0	0	0	0	0	0	0	0	0	0	0	0	0	0	0
HTR3A	3359	symbol	H. sapiens	3359	H. sapiens	HTR3A	5-hydroxytr	GO:0007210 serotonin		Transporters:Transport		Ziprasidone; Methadone; Olanzapir			1	1	0	0	1	0	0	0	0	0	0	0	0	0	0	0	0	0	0	0
IGFN1	91156	symbol	H. sapiens	91156	H. sapiens	IGFN1	immunoglo	GO:0010842 retina laye		Predicted ir	Midbody ring (Approved)				0	0	0	0	0	0	0	0	0	0	0	0	0	1	0	0	0	0	0	0
OLIG1	116448	symbol	H. sapiens	116448	H. sapiens	OLIG1	oligodendro	GO:0014003 oligodend		Transcription factors:Basic domains; Predicted intracellular					0	0	0	0	0	0	0	0	0	0	0	0	0	0	0	0	0	0	0	0
SYT16	83851	symbol	H. sapiens	83851	H. sapiens	SYT16	synaptotag	GO:0006887 exocytosis		Predicted ir	Cytosol (Approved)				0	0	0	0	0	0	0	0	0	0	0	0	0	0	1	0	0	0	0	0
HTR1E	3354	symbol	H. sapiens	3354	H. sapiens	HTR1E	5-hydroxytr	GO:0007198 adenylate		G-protein coupled rece		Ziprasidone; Methysergide; Zolmitr			1	0	0	1	1	0	0	0	0	0	0	0	0	0	0	0	0	0	0	0
PITX2	5308	symbol	H. sapiens	5308	H. sapiens	PITX2	paired like h	GO:0060127 prolactin s		Human dise	Nucleoplasm (Enhanced		(M1315)SIG	(M5923)HA	0	0	0	0	0	0	0	0	0	0	1	0	0	0	0	0	0	0	0	0
ASIC1	41	symbol	H. sapiens	41	H. sapiens	ASIC1	acid sensing	GO:0050915 sensory perception of			Golgi appar	Amiloride			1	1	0	0	0	0	0	0	0	0	0	1	0	0	0	0	0	1	0	0
GRM5	2915	symbol	H. sapiens	2915	H. sapiens	GRM5	glutamate r	GO:0007206 phospholi		G-protein coupled rece		Acamprosate; ADX10059; Rufinami			1	0	0	1	1	0	0	0	0	1	0	1	0	1	1	0	0	1	0	0
PABPC1L2B	645974	symbol	H. sapiens	645974	H. sapiens	PABPC1L2B	poly(A) binding protein cytoplasmic			Predicted intracellular proteins					0	0	0	0	0	0	0	0	0	0	0	0	0	0	0	1	0	0	0	0
NTNG2	84628	symbol	H. sapiens	84628	H. sapiens	NTNG2	netrin G2	GO:0098698 postsynap		Disease related genes; Predicted in			(M5887)NABA BASEME		0	0	0	0	0	0	0	0	0	0	0	1	0	1	0	0	0	0	0	0
CLDN19	149461	symbol	H. sapiens	149461	H. sapiens	CLDN19	claudin 19	GO:0150111 regulation		Disease related genes; Potential drug targets; T				(M5915)HA	1	0	0	0	0	0	1	0	0	0	0	0	0	1	0	0	0	0	0	0
MMP13	4322	symbol	H. sapiens	4322	H. sapiens	MMP13	matrix meta	GO:0003417 growth pla		Human disease related		Marimastat	(M174)PID I	(M5921)HA	0	0	0	0	0	1	1	0	0	0	0	0	0	0	0	0	0	0	0	1
OCLN	100506658	symbol	H. sapiens	100506658	H. sapiens	OCLN	occludin	GO:1902463 protein loc		Human dise	Cell Junctions;Plasma m		(M286)PID TGFBR PATH		0	0	0	0	0	0	1	1	0	1	0	0	0	1	0	0	0	0	0	0
FLNC	2318	symbol	H. sapiens	2318	H. sapiens	FLNC	filamin C	GO:0045214 sarcomere		Disease rela	Cytosol;Plasma membra		(M16801)SI	(M5915)HA	0	0	1	0	0	0	0	0	0	0	0	0	0	0	0	0	0	0	0	0
PASD1	139135	symbol	H. sapiens	139135	H. sapiens	PASD1	PAS domain	GO:0042754 negative r		Predicted ir	Nuclear speckles (Enhanced)				0	0	0	0	0	0	0	0	0	0	0	0	0	0	0	0	0	0	0	0
CCL20	6364	symbol	H. sapiens	6364	H. sapiens	CCL20	C-C motif ch	GO:0072679 thymocyte		Predicted secreted proteins			(M5883)NA	(M5890)HA	0	0	0	1	0	1	0	0	0	0	0	0	0	0	0	0	0	0	1	0
AK5	26289	symbol	H. sapiens	26289	H. sapiens	AK5	adenylate k	GO:0006173 dADP bios		ENZYME pr	Cytosol (Supported); Additional: Centriolar sate				0	0	0	0	0	0	0	0	0	0	0	0	0	0	0	0	0	0	0	0
GFPT2	9945	symbol	H. sapiens	9945	H. sapiens	GFPT2	glutamine-f	GO:0006048 UDP-N-ace		ENZYME pr	Vesicles (Approved)			(M5890)HA	0	0	0	0	0	0	0	0	0	0	0	0	0	0	0	0	0	0	0	0
CCDC144A	9720	symbol	H. sapiens	9720	H. sapiens	CCDC144A	coiled-coil domain containing 144A			Predicted ir	Nucleoplasm;Vesicles (Approved)				0	0	0	0	0	0	0	0	0	0	0	0	0	0	0	0	0	0	0	0
ARHGAP33	115703	symbol	H. sapiens	115703	H. sapiens	ARHGAP33	Rho GTPase	GO:0007264 small GTPa		Predicted ir	Plasma membrane (Approved); Additional: Acti				0	0	0	0	0	0	0	0	0	0	0	0	0	0	0	0	0	0	0	0
SLC25A21	89874	symbol	H. sapiens	89874	H. sapiens	SLC25A21	solute carri	GO:1990550 mitochond		Transporters:Electrochemical Potential-driven transporters					0	0	0	0	0	0	0	0	1	0	0	0	0	0	0	0	0	0	0	0
LRP2	4036	symbol	H. sapiens	4036	H. sapiens	LRP2	LDL recepto	GO:0070447 positive re		Human dise	Mitochondr	Urokinase; I	(M211)PID HEDGEHOG		0	1	1	0	0	0	1	0	1	0	0	0	1	1	0	0	0	0	1	0
LCE1A	353131	symbol	H. sapiens	353131	H. sapiens	LCE1A	late cornifie	GO:0031424 keratinizat		Predicted intracellular proteins					0	0	0	0	0	0	0	0	0	0	1	0	0	0	0	0	0	0	0	0
SOX2	6657	symbol	H. sapiens	6657	H. sapiens	SOX2	SRY-box tra	GO:0001714 endoderm		Transcriptic	Nucleoplasm (Enhanced)				0	0	0	0	0	0	0	0	0	0	0	0	0	0	0	0	0	0	0	0

KRTAP4-1	85285	symbol	H. sapiens	85285	H. sapiens	KRTAP4-1	keratin associated protein 4-1		Predicted intracellular proteins					0	0	0	0	0	0	0	0	0	0	0	0	0	0	0	0	0	0	0	0
CHRNA4	1137	symbol	H. sapiens	1137	H. sapiens	CHRNA4	cholinergic	GO:0035095 behavioral	FDA approved drug targ		Nicotine; Butalbital; Talbutal; Pento			1	1	0	1	0	0	0	0	0	1	0	0	0	0	1	0	0	1	0	0
NPHS2	7827	symbol	H. sapiens	7827	H. sapiens	NPHS2	NPHS2 ston	GO:0072244 metaneph	Disease related genes; Human disea			(M193)PID NEPHRIN NE		0	0	1	0	0	0	0	0	0	0	0	0	0	0	0	0	0	0	0	0
MTRNR2L3	100462983	symbol	H. sapiens	100462983	H. sapiens	MTRNR2L3	MT-RNR2 lil	GO:1900118 negative r	Predicted intracellular proteins; Predicted secreted protein					0	0	0	0	0	0	0	0	0	0	0	0	0	0	0	0	0	0	0	0
LRRTM2	26045	symbol	H. sapiens	26045	H. sapiens	LRRTM2	leucine rich	GO:0099151 regulation	Predicted intracellular proteins					1	0	0	0	0	0	0	0	0	0	0	1	0	1	1	0	0	0	0	0
CLVS2	134829	symbol	H. sapiens	134829	H. sapiens	CLVS2	clavesin 2	GO:0007040 lysosome o	Predicted intracellular proteins					0	0	0	0	0	0	0	0	0	0	0	0	0	0	0	0	0	0	0	0
SOX21	11166	symbol	H. sapiens	11166	H. sapiens	SOX21	SRY-box tra	GO:0001942 hair follicle	Transcriptic	Nucleoplasm (Approved)				0	0	0	0	0	0	0	0	0	0	1	0	0	0	0	0	0	0	0	0
C4orf45	152940	symbol	H. sapiens	152940	H. sapiens	C4orf45	chromosome 4 open reading frame		Predicted intracellular proteins					0	0	0	0	0	0	0	0	0	0	0	0	0	0	0	0	0	0	0	0
GALNTL6	442117	symbol	H. sapiens	442117	H. sapiens	GALNTL6	polypeptide	GO:0018243 protein O-	ENZYME proteins:Transferases; Predicted intracellular prot					0	0	0	0	0	0	0	0	0	0	0	0	0	0	0	0	0	0	0	0
HCN2	610	symbol	H. sapiens	610	H. sapiens	HCN2	hyperpolari	GO:0071321 cellular res	Voltage-gated ion chan		Cyclic GMP; Cyclic adenosine mono			1	1	0	0	1	0	0	0	0	1	0	0	0	0	0	0	0	0	0	0
AGAP9	642517	symbol	H. sapiens	642517	H. sapiens	AGAP9	ArfGAP with	GO:0050790 regulation	Predicted intracellular proteins					0	0	0	0	0	0	0	0	0	0	0	0	0	0	0	0	0	0	0	0
ARHGAP5	394	symbol	H. sapiens	394	H. sapiens	ARHGAP5	Rho GTPase	GO:0007266 Rho protei	Predicted ir	Cytosol;Endoplasmic re		(M68)PID R	(M5893)HA	0	0	0	0	0	0	0	0	0	0	0	0	0	0	0	0	0	0	0	0
GNRHR	2798	symbol	H. sapiens	2798	H. sapiens	GNRHR	gonadotrop	GO:0097210 response t	Transporters; Human di		Leuprolide; Goserelin; Cetrorelix; Al			0	0	0	1	1	0	0	0	0	0	0	0	0	0	0	0	0	0	0	0
IFIT1B	439996	symbol	H. sapiens	439996	H. sapiens	IFIT1B	interferon i	GO:0071360 cellular res	Predicted intracellular proteins					0	0	0	0	1	0	0	0	0	0	0	0	0	0	0	0	0	0	0	0
PON1	5444	symbol	H. sapiens	5444	H. sapiens	PON1	paraoxonas	GO:1902617 response t	ENZYME proteins:Hydro		Cefazolin; Zinc; Copper; Zinc acetate			0	0	0	0	0	0	0	0	0	1	0	0	0	0	0	0	0	0	1	0
LIX1	167410	symbol	H. sapiens	167410	H. sapiens	LIX1	limb and CN	GO:0097352 autophago	Predicted ir	Cytosol (Approved); Additional: Cytokinetic bric				0	0	0	0	0	0	0	0	0	0	0	0	0	0	0	0	0	0	0	0
EGR1	1958	symbol	H. sapiens	1958	H. sapiens	EGR1	early growt	GO:0072303 positive re	Transcriptic	Nucleoplasm (Enhanced		(M11)PID PI	(M5890)HA	0	0	1	0	1	1	0	1	0	0	0	0	0	0	0	0	0	1	1	1
PRRT2	112476	symbol	H. sapiens	112476	H. sapiens	PRRT2	proline rich	GO:1905512 regulation	Human dise	Plasma membrane (Supported)				1	0	0	0	0	0	0	0	0	0	0	1	0	0	1	0	0	0	0	0
NOS1	4842	symbol	H. sapiens	4842	H. sapiens	NOS1	nitric oxide	GO:0098923 retrograde	Enzymes; El	Plasma mer	Citrulline; Donepezil; Ke		(M5951)HA	1	0	1	0	0	0	1	0	0	1	0	1	0	0	0	0	0	0	1	0
BAAT	570	symbol	H. sapiens	570	H. sapiens	BAAT	bile acid-Co	GO:0002152 bile acid c	Human dise	Vesicles (Ur	Glycine			0	0	0	0	0	0	0	0	0	0	0	0	0	0	0	0	0	0	1	0
CYP1A2	1544	symbol	H. sapiens	1544	H. sapiens	CYP1A2	cytochrome	GO:0009403 toxin biosy	ENZYME proteins:Oxido		Isoniazid; alpha-Naphth		(M5934)HA	0	0	0	0	0	0	0	0	0	0	0	0	0	0	0	0	0	0	1	0
GPRC5A	9052	symbol	H. sapiens	9052	H. sapiens	GPRC5A	G protein-c	GO:0007175 negative r	G-protein c	Plasma mer	Tretinoin			0	0	0	1	0	0	0	0	0	0	0	0	0	0	0	0	0	0	0	0
SFTPB	6439	symbol	H. sapiens	6439	H. sapiens	SFTPB	surfactant p	GO:0007585 respiratory	Disease related genes; Human disea			(M5880)NABA ECM AFF		0	0	0	0	0	1	0	0	0	0	0	0	0	0	0	0	0	0	0	0
CILP	8483	symbol	H. sapiens	8483	H. sapiens	CILP	cartilage int	GO:0043569 negative r	Disease related genes; Predicted se			(M3008)NABA ECM GLY		0	0	0	0	0	0	0	0	0	0	0	0	0	0	0	0	1	0	0	0
HPX	3263	symbol	H. sapiens	3263	H. sapiens	HPX	hemopexin	GO:0060332 positive re	Predicted secreted prot		Zinc chlorid	(M5880)NABA ECM AFF		0	1	0	0	0	1	0	0	0	0	0	0	0	0	0	0	0	0	1	0
FAM193B	54540	symbol	H. sapiens	54540	H. sapiens	FAM193B	family with sequence similarity 193		Predicted ir	Nucleoli;Nucleoplasm (Enhanced)				0	0	0	0	0	0	0	0	0	0	0	0	0	0	0	0	0	0	0	0
PITX1	5307	symbol	H. sapiens	5307	H. sapiens	PITX1	paired like h	GO:0014707 branchiom	Disease rela	Nucleoli (Approved)				0	0	1	0	0	0	0	0	0	0	0	0	0	0	0	0	0	0	0	1
CXCL1	2919	symbol	H. sapiens	2919	H. sapiens	CXCL1	C-X-C motif	GO:0061844 antimicrob	Predicted secreted proteins; Cancer			(M196)PID I	(M5897)HA	0	0	0	1	0	1	0	0	0	0	0	0	0	0	0	0	0	0	0	1
FAM83A	84985	symbol	H. sapiens	84985	H. sapiens	FAM83A	family with	GO:0007173 epidermal	Predicted ir	Nucleoplasm (Approved); Additional: Cytosol				0	0	0	0	0	1	0	0	0	0	0	0	0	0	0	0	0	0	0	0
CDKN2A	1029	symbol	H. sapiens	1029	H. sapiens	CDKN2A	cyclin depe	GO:2000111 positive re	Human dise	Nucleoli (Supported)		(M3686)SA	(M5925)HA	0	0	0	0	0	0	1	0	0	0	0	0	0	0	0	0	0	0	0	0
AIRE	326	symbol	H. sapiens	326	H. sapiens	AIRE	autoimmun	GO:0002458 peripheral	Disease related genes; Transcription factors:alpha-Helices e					0	0	0	0	0	0	0	0	0	0	0	0	0	0	0	0	0	0	0	0
PLEKHS1	79949	symbol	H. sapiens	79949	H. sapiens	PLEKHS1	pleckstrin homology domain contai		Predicted ir	Centrosome (Approved); Additional: Vesicles				0	0	0	0	0	0	0	0	0	0	0	0	0	0	0	0	0	0	0	0
RCN3	57333	symbol	H. sapiens	57333	H. sapiens	RCN3	reticulocalb	GO:0032964 collagen b	Predicted ir	Endoplasmic reticulum (Supported); Additional				0	0	0	0	0	0	0	1	0	0	0	0	0	0	1	0	0	0	0	1
MAPK8IP3	23162	symbol	H. sapiens	23162	H. sapiens	MAPK8IP3	mitogen-act	GO:0099640 axo-dendr	Disease rela	Vesicles (Approved)		(M17200)SA B CELL REC		0	0	0	0	0	0	0	0	0	0	0	0	0	0	0	0	0	0	0	0
NBEAL1	65065	symbol	H. sapiens	65065	H. sapiens	NBEAL1	neurobeach	GO:0008104 protein loc	Predicted ir	Nucleoplasm (Approved); Additional: Cytosol				0	0	0	0	0	0	0	0	0	0	0	0	0	0	0	0	0	0	0	0
IGF2BP2	10644	symbol	H. sapiens	10644	H. sapiens	IGF2BP2	insulin like g	GO:0070934 CRD-media	Predicted ir	Cytosol (Enhanced)				0	0	0	0	0	0	0	0	0	0	0	0	0	0	0	0	0	0	0	0
CNTN3	5067	symbol	H. sapiens	5067	H. sapiens	CNTN3	contactin 3	GO:0007155 cell adhesi	Predicted intracellular proteins					0	0	0	0	0	0	0	0	0	0	0	0	0	0	0	0	0	0	0	0
CLSTN2	64084	symbol	H. sapiens	64084	H. sapiens	CLSTN2	calsyntenin	GO:0051965 positive regulation of s		Vesicles (Uncertain); Additional: Cytokinetic bri				0	0	0	0	0	0	0	0	0	0	0	1	0	1	0	0	0	0	0	0
SHPRH	257218	symbol	H. sapiens	257218	H. sapiens	SHPRH	SNF2 histon	GO:0006334 nucleosom	ENZYME pro	Nuclear membrane;Nucleoplasm (Approved); A				0	0	0	0	0	0	0	0	0	0	0	0	0	0	0	0	0	0	0	0
KCNV1	27012	symbol	H. sapiens	27012	H. sapiens	KCNV1	potassium v	GO:0071805 potassium	Voltage-gat	Cytokinetic bridge;Plasma membrane (Supporte				1	1	0	0	0	0	0	0	0	1	0	0	0	0	0	0	0	0	0	0
GOLGA8A	23015	symbol	H. sapiens	23015	H. sapiens	GOLGA8A	golgin A8 fa	GO:0051225 spindle ass	Predicted ir	Golgi apparatus (Approved); Additional: Cytoso				0	0	0	0	0	0	0	0	0	0	0	0	0	0	0	0	0	0	0	0
PRKCG	5582	symbol	H. sapiens	5582	H. sapiens	PRKCG	protein kina	GO:003242 AGC Ser/Th	Human disease related		Fostamatini	(M252)PID I	(M5950)HA	1	0	0	1	1	1	1	0	0	1	0	1	0	0	0	0	0	1	0	0
FAM47E-ST	100631383	symbol	H. sapiens	100631383	H. sapiens	FAM47E-ST	FAM47E-STBD1 readthrough		Predicted ir	Cytosol;Plasma membrane (Uncertain)				0	0	0	0	0	0	0	0	0	0	0	0	0	0	0	0	0	0	0	0
FRMD7	90167	symbol	H. sapiens	90167	H. sapiens	FRMD7	FERM doma	GO:0010592 positive re	Human dise	Cytosol;Plasma membrane (Supported); Additic				0	0	0	0	0	0	0	0	0	0	0	0	0	0	0	0	0	0	0	0

GABRB3	2562	symbol	H. sapiens	2562	H. sapiens	GABRB3	gamma-ami	GO:0071420 cellular re	Disease related genes; I		Lindane; Piperazine; Ivermectin; Pr			1	1	0	1	1	0	0	0	1	0	0	0	0	1	0	0	0	0	0	0
RHPN1	114822	symbol	H. sapiens	114822	H. sapiens	RHPN1	rhophilin R	GO:0051497 negative r	Predicted ir	Nucleoplasm (Approved)				0	0	0	0	0	0	0	0	0	0	0	0	0	0	0	0	0	0	0	0
CENPT	80152	symbol	H. sapiens	80152	H. sapiens	CENPT	centromere	GO:0051382 kinetocho	Disease rel	Nuclear bodies (Supported); Additional: Nucleo				0	0	0	0	0	0	0	0	0	0	0	0	0	0	0	0	0	0	0	0
RIMBP2	23504	symbol	H. sapiens	23504	H. sapiens	RIMBP2	RIMS bindir	GO:0007274 neuromus	Predicted ir	Nucleoplasm (Approved); Additional: Cytosol;G				1	0	0	0	0	0	0	0	0	0	0	0	0	0	0	0	0	0	0	0
COL19A1	1310	symbol	H. sapiens	1310	H. sapiens	COL19A1	collagen ty	GO:0007519 skeletal m	Predicted intracellular proteins; Pre			(M3005)NABA COLLAG		0	0	1	0	0	0	0	0	0	0	0	0	0	0	0	0	0	0	0	1
PLG	5340	symbol	H. sapiens	5340	H. sapiens	PLG	plasminoge	GO:0051919 positive re	FDA approved drug targ		Alteplase; L	(M165)PID	(M5919)HA	1	0	1	1	0	1	1	1	0	0	0	0	0	0	0	0	0	0	0	1
MFSD2A	84879	symbol	H. sapiens	84879	H. sapiens	MFSD2A	major facili	GO:0140329 lysophosp	Disease rel	Cytosol (Approved); Additional: Cytoplasmic bo				0	0	0	0	0	0	0	1	1	0	0	0	0	0	0	0	0	1	0	0
GMNC	647309	symbol	H. sapiens	647309	H. sapiens	GMNC	geminin coi	GO:0006270 DNA replic	Predicted intracellular proteins					0	0	0	0	0	0	0	0	0	0	0	0	0	0	0	0	0	0	0	0
IGFBP1	3484	symbol	H. sapiens	3484	H. sapiens	IGFBP1	insulin like	GO:0043567 regulation	Predicted s	Golgi apparatus (Appro		(M106)PID I	(M5922)HA	0	0	0	0	1	0	0	0	0	0	0	0	0	0	0	0	1	0	1	0
PPP2R2C	5522	symbol	H. sapiens	5522	H. sapiens	PPP2R2C	protein pho	GO:0070262 peptidyl-s	Predicted ir	Cytosol (Approved)			(M5915)HA	0	0	0	0	0	0	0	0	0	0	0	0	0	0	0	1	0	0	0	0
KLHL11	55175	symbol	H. sapiens	55175	H. sapiens	KLHL11	kelch like family member 11		Predicted ir	Nucleoplasm (Approved); Additional: Nuclear m				0	0	0	0	0	0	0	0	0	0	0	0	0	0	0	0	0	0	0	0
DMRT3	58524	symbol	H. sapiens	58524	H. sapiens	DMRT3	doublesex a	GO:0021521 ventral spi	Transcription factors:Zinc-coordinating DNA-binding domai					0	0	0	0	0	0	0	0	0	0	0	0	0	0	0	0	0	1	0	0
KRTAP24-1	643803	symbol	H. sapiens	643803	H. sapiens	KRTAP24-1	keratin associated protein 24-1		Predicted intracellular proteins					0	0	0	0	0	0	0	0	0	0	0	0	0	0	0	0	0	0	0	0
GNRH1	2796	symbol	H. sapiens	2796	H. sapiens	GNRH1	gonadotrop	GO:2000354 regulation	Disease related genes; Predicted se			(M213)PID AR NONGEN		0	0	0	1	0	0	0	0	0	0	0	0	0	0	0	0	0	0	0	0
ZBTB20	26137	symbol	H. sapiens	26137	H. sapiens	ZBTB20	zinc finger a	GO:0045821 positive re	Disease rel	Nuclear bodies;Nucleoplasm (Enhanced)				0	0	0	0	0	0	0	0	0	0	0	0	0	0	0	0	0	0	0	0
RNF207	388591	symbol	H. sapiens	388591	H. sapiens	RNF207	ring finger	GO:1903952 regulation	Predicted ir	Cytosol (Approved)				1	0	0	0	0	0	0	0	0	1	0	0	0	0	0	0	0	0	0	0
LUC7L	55692	symbol	H. sapiens	55692	H. sapiens	LUC7L	LUC7 like	GO:0045843 negative r	Predicted ir	Nucleoplasm (Approved); Additional: Mitochon				0	0	0	0	0	0	0	0	0	0	0	0	0	0	0	0	0	0	0	0
KRTAP5-9	3846	symbol	H. sapiens	3846	H. sapiens	KRTAP5-9	keratin ass	GO:0008544 epidermis	Predicted intracellular proteins					0	0	0	0	0	0	0	0	0	0	1	0	0	0	0	0	0	0	0	0
LTB4R	1241	symbol	H. sapiens	1241	H. sapiens	LTB4R	leukotriene	GO:0061737 leukotrien	G-protein coupled rece		Amelubant; Morniflumate; Leukotr			0	0	0	1	0	0	0	0	0	0	0	0	0	0	0	0	0	0	0	0
STRA6	64220	symbol	H. sapiens	64220	H. sapiens	STRA6	signaling re	GO:0061143 alveolar p	Disease related genes; Human disease related genes:Conge					0	1	1	0	0	0	0	0	0	0	0	0	1	0	0	0	0	1	0	0
KLF17	128209	symbol	H. sapiens	128209	H. sapiens	KLF17	Kruppel like	GO:0006357 regulation	Transcription factors:Zinc-coordinating DNA-binding domai					0	0	0	0	0	0	0	0	0	0	0	0	0	0	0	0	0	0	0	0
TMEM74B	55321	symbol	H. sapiens	55321	H. sapiens	TMEM74B	transmembrane protein 74B			Golgi apparatus;Nucleoplasm (Approved)				0	0	0	0	0	0	0	0	0	0	0	0	0	0	0	0	0	0	0	0
EDAR	10913	symbol	H. sapiens	10913	H. sapiens	EDAR	ectodysplas	GO:0060605 tube lume	Disease related genes; Human disease related				(M5956)HA	0	0	0	0	0	1	0	0	0	0	1	0	0	0	0	0	0	0	0	0
SERPINE1	5054	symbol	H. sapiens	5054	H. sapiens	SERPINE1	serpin famil	GO:0035490 regulation	Human dise	Cytosol (Ap	Alteplase; L	(M44)PID H	(M5896)HA	0	0	0	0	0	1	1	0	0	0	1	0	0	0	0	0	0	0	0	1
CUBN	8029	symbol	H. sapiens	8029	H. sapiens	CUBN	cubilin	GO:0015889 cobalamin	Disease related genes;		Hydroxocobalamin			0	0	0	0	0	0	1	1	0	0	0	0	1	0	0	0	0	0	1	0
BASP1	10409	symbol	H. sapiens	10409	H. sapiens	BASP1	brain abunc	GO:2001074 regulation	Predicted ir	Plasma membrane (Enhanced)			(M5930)HA	0	0	1	0	0	0	0	0	0	0	0	0	0	0	0	0	0	0	0	0
KIF18B	146909	symbol	H. sapiens	146909	H. sapiens	KIF18B	kinesin fam	GO:0007019 microtubu	Predicted ir	Microtubule ends;Nucleoplasm (Su			(M5925)HA	0	0	0	0	0	0	1	0	0	0	0	0	0	0	0	0	0	0	0	0
ZNF460	10794	symbol	H. sapiens	10794	H. sapiens	ZNF460	zinc finger	GO:0006357 regulation	Transcriptic	Nucleoplasm (Approved)				0	0	0	0	0	0	0	0	0	0	0	0	0	0	0	0	0	0	0	0
F3	2152	symbol	H. sapiens	2152	H. sapiens	F3	coagulation	GO:0002541 activation	Predicted s	Vesicles (Ap	Coagulation factor VIIa		(M5946)HA	0	0	0	0	0	0	1	0	0	0	0	0	0	0	0	0	0	0	0	0
CIDEC	63924	symbol	H. sapiens	63924	H. sapiens	CIDEC	cell death ir	GO:0034389 lipid dropl	Disease related genes; Predicted intracellular proteins; Hun					0	0	0	0	0	0	0	0	0	0	0	0	0	0	0	0	0	0	0	0
LHX8	431707	symbol	H. sapiens	431707	H. sapiens	LHX8	LIM homeo	GO:0021884 forebrain	Transcriptic	Nucleoli;Nucleoplasm (Approved); Additional:				0	0	0	0	0	0	0	0	0	0	1	0	1	0	0	0	0	1	0	0
PPP1R1A	5502	symbol	H. sapiens	5502	H. sapiens	PPP1R1A	protein pho	GO:0005977 glycogen	Predicted intracellular proteins					0	0	0	0	0	0	0	0	0	0	0	0	0	0	0	0	0	0	0	0
MTRNR2L1	100462977	symbol	H. sapiens	100462977	H. sapiens	MTRNR2L1	MT-RNR2 li	GO:1900118 negative r	Predicted intracellular proteins; Predicted secreted protein					0	0	0	0	0	0	0	0	0	0	0	0	0	0	0	0	0	0	0	0
CALN1	83698	symbol	H. sapiens	83698	H. sapiens	CALN1	calneuron 1		Predicted ir	Vesicles (Uncertain); Additional: Plasma membr				0	0	0	0	0	0	0	0	0	0	0	0	0	0	0	0	0	0	0	0
NIPAL4	348938	symbol	H. sapiens	348938	H. sapiens	NIPAL4	NIPA like d	GO:1903830 magnesiu	Disease related genes; Human disease related genes:Conge					0	1	0	0	0	0	0	0	0	0	0	0	0	0	0	0	0	0	0	0
STX1A	6804	symbol	H. sapiens	6804	H. sapiens	STX1A	syntaxin 1A	GO:0010701 positive re	Transporter	Vesicles (Approved); Additional: Nuclear memb				1	0	0	0	0	0	0	0	0	1	0	1	0	0	1	0	0	0	0	0
MLLT11	10962	symbol	H. sapiens	10962	H. sapiens	MLLT11	MLLT11 tra	GO:0051901 positive re	Disease rel	Cytosol;Nucleoplasm (Supported)			(M5924)HA	1	0	0	0	0	0	0	0	0	0	0	0	0	0	0	0	0	0	0	0
SYT10	341359	symbol	H. sapiens	341359	H. sapiens	SYT10	synaptotag	GO:0045956 positive regulation of		Nucleoplasm;Vesicles (Approved)				1	0	0	0	0	0	0	0	0	1	0	0	0	0	1	0	0	0	0	0
ZNF692	55657	symbol	H. sapiens	55657	H. sapiens	ZNF692	zinc finger	GO:0006111 regulation	Transcriptic	Nucleoli;Nucleoplasm (Enhanced)				0	0	0	0	0	0	0	0	0	0	0	0	0	0	0	0	0	0	0	0
ANKRD63	100131244	symbol	H. sapiens	100131244	H. sapiens	ANKRD63	ankyrin repeat domain 63		Predicted ir	Mitochondria (Approved)				0	0	0	0	0	0	0	0	0	0	0	0	0	0	0	0	0	0	0	0
CLIC5	53405	symbol	H. sapiens	53405	H. sapiens	CLIC5	chloride int	GO:0006749 glutathion	Human dise	Plasma membrane (Supported); Additional: Nu				0	0	0	0	0	0	0	0	1	1	0	0	0	0	0	0	0	0	0	0
TMEM132D	121256	symbol	H. sapiens	121256	H. sapiens	TMEM132D	transmembrane protein 132D							0	0	0	0	0	0	0	0	0	0	0	0	0	0	0	0	0	0	0	0
FCRL2	79368	symbol	H. sapiens	79368	H. sapiens	FCRL2	Fc receptor	GO:0007267 cell-cell si	CD markers					0	0	0	0	0	0	0	0	0	0	0	0	0	0	0	0	0	0	0	0
BTBD18	643376	symbol	H. sapiens	643376	H. sapiens	BTBD18	BTB domair	GO:1990511 piRNA bio	Predicted ir	Nucleoplasm;Plasma membrane (Approved); A				0	0	0	0	0	0	0	0	0	0	0	0	0	0	0	0	0	0	0	0

CCNL2	81669	symbol	H. sapiens	81669	H. sapiens	CCNL2	cyclin L2	GO:0046605 regulation	Predicted ir	Nucleoplasm (Supported)				0	0	0	0	0	0	0	0	0	0	0	0	0	0	0	0	0	0	0	0
IGSF23	147710	symbol	H. sapiens	147710	H. sapiens	IGSF23	immunoglobulin superfamily memb		Predicted ir	Nucleoplasm (Approved); Additional: Cell Junct				0	0	0	0	0	0	0	0	0	0	0	0	0	0	0	0	0	0	0	0
ZPBP2	124626	symbol	H. sapiens	124626	H. sapiens	ZPBP2	zona pelluci	GO:0002638 negative r	Predicted secreted proteins					0	0	0	0	0	0	0	0	0	0	0	0	0	0	0	0	0	0	0	0
GLRA2	2742	symbol	H. sapiens	2742	H. sapiens	GLRA2	glycine rece	GO:0060012 synaptic tr	Transporters:Transport		Glycine; Lindane; Picrotoxin; Ethanc			1	1	0	1	1	0	0	0	1	0	0	0	0	0	0	0	0	0	0	0
MXD3	83463	symbol	H. sapiens	83463	H. sapiens	MXD3	MAX dimeri	GO:0000122 negative r	Transcriptic	Nucleoli;Nucleoplasm (Approved)			(M5925)HA	0	0	0	0	0	0	0	0	0	0	0	0	0	0	0	0	0	0	0	0
PTK6	5753	symbol	H. sapiens	5753	H. sapiens	PTK6	protein tyrc	GO:000726(Tyr protein	Enzymes; El	Nucleoplasr	Vandetanib; Fostamatinib; Zanubru			0	0	0	0	0	0	0	0	0	0	0	0	0	0	0	0	0	0	0	0
TRIM63	84676	symbol	H. sapiens	84676	H. sapiens	TRIM63	tripartite m	GO:0014878 response t	ENZYME proteins:Transferases; Predicted intracellular prot					0	0	0	0	0	0	0	0	0	1	0	0	0	0	0	0	0	0	0	0
PABPN1	8106	symbol	H. sapiens	8106	H. sapiens	PABPN1	poly(A) binc	GO:1904247 positive re	Disease rela	Nucleoplasm (Approved); Additional: Nuclear s				0	0	0	0	0	0	0	0	0	0	0	0	0	0	0	1	0	0	0	0
PABPC1L2A	340529	symbol	H. sapiens	340529	H. sapiens	PABPC1L2A	poly(A) binding protein cytoplasmic		Predicted intracellular proteins					0	0	0	0	0	0	0	0	0	0	0	0	0	0	0	1	0	0	0	0
ADAMTS14	140766	symbol	H. sapiens	140766	H. sapiens	ADAMTS14	ADAM met	GO:0030574 collagen c	Predicted secreted proteins; Cance			(M3468)NABA ECM REC		0	0	0	0	0	1	1	0	0	0	0	0	0	0	0	0	0	0	0	1
CHAC1	79094	symbol	H. sapiens	79094	H. sapiens	CHAC1	ChaC glutat	GO:0006751 glutathion	Predicted ir	Mitochondria (Approved)			(M5922)HA	0	0	0	0	0	0	0	0	0	0	0	0	0	0	0	0	0	0	0	0
NOL4	8715	symbol	H. sapiens	8715	H. sapiens	NOL4	nucleolar protein 4		Predicted ir	Nucleoplasm (Supported)				0	0	0	0	0	0	0	0	0	0	0	0	0	0	0	0	0	0	0	0
TRIM72	493829	symbol	H. sapiens	493829	H. sapiens	TRIM72	tripartite m	GO:0043569 negative r	Predicted ir	Vesicles (Approved); Additional: Nucleoplasm				0	0	1	0	0	0	0	0	0	0	0	0	0	0	1	0	1	0	0	0
CXCL3	2921	symbol	H. sapiens	2921	H. sapiens	CXCL3	C-X-C motif	GO:0061844 antimicrob	Predicted secreted proteins			(M5883)NA	(M5897)HA	0	0	0	1	0	1	0	0	0	0	0	0	0	0	0	0	0	0	0	0
SOCS1	8651	symbol	H. sapiens	8651	H. sapiens	SOCS1	suppressor	GO:0043377 negative r	Cancer-rela	Nucleoplasm (Supporte		(M1718)SIG	(M5897)HA	0	0	0	0	1	0	0	0	0	0	0	0	0	0	0	0	0	0	0	0
CRYGN	155051	symbol	H. sapiens	155051	H. sapiens	CRYGN	crystallin ga	GO:0002088 lens devel	Predicted intracellular proteins					0	0	0	0	0	0	0	0	0	0	0	0	0	0	0	0	0	0	0	0
SLC12A1	6557	symbol	H. sapiens	6557	H. sapiens	SLC12A1	solute carri	GO:0055064 chloride io	Disease rela	Vesicles (Ap	Torasemide; Methyclothiazide; Chlc			0	1	0	0	0	0	0	0	1	0	0	0	0	0	0	0	0	0	0	0
SERPINA3	12	symbol	H. sapiens	12	H. sapiens	SERPINA3	serpin famil	GO:0030277 maintenar	Predicted intracellular p		Zinc; Zinc ac	(M3468)NA	(M5907)HA	0	0	0	0	0	1	1	1	0	0	0	0	0	0	0	0	0	0	0	0
KCNN4	3783	symbol	H. sapiens	3783	H. sapiens	KCNN4	potassium c	GO:0046541 saliva secr	FDA approv	Cytosol;Plas	Clotrimazole; Quinine; I		(M5953)HA	1	1	0	0	0	0	1	0	0	1	0	0	0	0	1	0	0	0	0	0
MCIDAS	345643	symbol	H. sapiens	345643	H. sapiens	MCIDAS	multiciliate	GO:1903251 multi-ciliat	Disease rela	Nuclear bodies (Supported)				0	0	0	0	0	0	0	0	0	0	0	0	0	0	0	0	0	0	0	0
GOLGA8B	440270	symbol	H. sapiens	440270	H. sapiens	GOLGA8B	golgin A8 fa	GO:0051225 spindle as	Predicted ir	Golgi apparatus (Approved); Additional: Cytoso				0	0	0	0	0	0	0	0	0	0	0	0	0	0	0	0	0	0	0	0
HJURP	55355	symbol	H. sapiens	55355	H. sapiens	HJURP	Holliday jun	GO:0034080 CENP-A co	Predicted ir	Nucleoli;Nucleoli rim;Nucleoplasm (Enhanced);				0	0	0	0	0	0	0	0	0	0	0	0	0	0	0	0	0	0	0	0
SALL4	57167	symbol	H. sapiens	57167	H. sapiens	SALL4	spalt like tra	GO:0001833 inner cell r	Human dise	Nucleoplasm (Enhance		(M223)PID BETA CATEN		0	0	0	0	0	0	0	0	0	0	0	0	0	0	0	0	0	0	0	0
PI15	51050	symbol	H. sapiens	51050	H. sapiens	PI15	peptidase ir	GO:0010466 negative r	Predicted se	Vesicles (Approved); Additional: Cytosol				0	0	0	0	0	0	0	0	0	0	0	0	0	0	0	0	0	0	0	0
TNFSF14	8740	symbol	H. sapiens	8740	H. sapiens	TNFSF14	TNF superfa	GO:0010820 positive re	Predicted ir	Vesicles (Approved)		(M5883)NABA SECRETE		0	0	0	0	0	1	0	0	0	0	0	0	0	0	0	0	0	0	0	0
KCNIP1	30820	symbol	H. sapiens	30820	H. sapiens	KCNIP1	potassium v	GO:1901379 regulation	Predicted ir	Vesicles (Approved)				0	1	0	0	0	0	0	0	0	1	0	0	0	0	0	0	0	0	0	0
CASR	846	symbol	H. sapiens	846	H. sapiens	CASR	calcium sen	GO:0032782 bile acid se	Human disease related		Spermine; N	(M184)PID ECADHERIN		0	1	0	1	1	0	0	0	1	1	0	0	0	0	1	0	0	1	0	1
NOTUM	147111	symbol	H. sapiens	147111	H. sapiens	NOTUM	notum, palr	GO:1990697 protein de	Predicted se	Endoplasmic reticulum (Approved)				0	0	0	0	0	0	0	0	0	0	0	0	0	0	0	0	0	0	0	0
PABPC1L	80336	symbol	H. sapiens	80336	H. sapiens	PABPC1L	poly(A) binding protein cytoplasmic		Predicted ir	Cytosol (Approved); Additional: Nuclear bodies				0	0	0	0	0	0	0	0	0	0	0	0	0	0	0	1	0	0	0	0
SLC4A10	57282	symbol	H. sapiens	57282	H. sapiens	SLC4A10	solute carri	GO:0021860 pyramidal	Transporters:Electrochemical Potential-driven transporters					0	1	0	0	0	0	0	0	1	0	0	1	0	0	0	0	0	1	0	0
ATP2A1	487	symbol	H. sapiens	487	H. sapiens	ATP2A1	ATPase sarc	GO:0106134 positive re	Predicted ir	Endoplasmi	Enflurane; Adenosine-5		(M5909)HA	0	1	0	0	0	0	1	0	0	1	0	0	0	0	0	0	0	0	0	0
OLFM4	10562	symbol	H. sapiens	10562	H. sapiens	OLFM4	olfactomed	GO:1900026 positive re	Predicted se	Nucleoplasm;Plasma membrane (Supported); A				0	0	0	0	0	0	1	0	0	0	0	0	0	0	0	0	0	0	0	0
ENTHD1	150350	symbol	H. sapiens	150350	H. sapiens	ENTHD1	ENTH doma	GO:0006897 endocytos	Predicted intracellular proteins					0	0	0	0	0	0	0	0	0	0	0	0	0	0	0	0	0	0	0	0
ZNF728	388523	symbol	H. sapiens	388523	H. sapiens	ZNF728	zinc finger p	GO:0006357 regulation	Predicted intracellular proteins					0	0	0	0	0	0	0	0	0	0	0	0	0	0	0	0	0	0	0	0
SPEG	10290	symbol	H. sapiens	10290	H. sapiens	SPEG	striated mu	GO:004269:CAMK Ser/T	Predicted ir	Vesicles (Approved)			(M5909)HA	0	0	1	0	0	0	0	0	0	0	0	0	0	0	0	0	0	0	0	0
PLCXD3	345557	symbol	H. sapiens	345557	H. sapiens	PLCXD3	phosphatid	GO:0016042 lipid catab	Predicted ir	Golgi apparatus (Approved)				0	0	0	0	0	0	0	0	0	0	0	0	0	0	0	0	0	0	0	0
TRIM49C	642612	symbol	H. sapiens	642612	H. sapiens	TRIM49C	tripartite m	GO:0016567 protein ub	Predicted intracellular proteins					0	0	0	0	0	0	0	0	0	0	0	0	0	0	0	0	0	0	0	0
HRK	8739	symbol	H. sapiens	8739	H. sapiens	HRK	harakiri, BC	GO:0090200 positive re	Predicted intracellular proteins			(M166)PID ATF2 PATHV		0	0	0	0	0	0	0	0	0	0	0	0	0	0	0	0	0	0	0	0
FOSB	2354	symbol	H. sapiens	2354	H. sapiens	FOSB	FosB proto-	GO:0051412 response t	Transcriptic	Nucleoplasm (Supporte		(M167)PID	(M5941)HA	0	0	0	0	0	1	0	0	0	0	0	0	0	0	0	0	0	0	0	0
USH1G	124590	symbol	H. sapiens	124590	H. sapiens	USH1G	USH1 prote	GO:0050957 equilibrioc	Disease related genes; Predicted intracellular proteins; Hun					0	0	0	0	0	0	0	1	0	0	0	0	0	0	0	0	0	0	0	0
EML5	161436	symbol	H. sapiens	161436	H. sapiens	EML5	EMAP like 5	GO:0008150 biological_	Predicted intracellular proteins					0	0	0	0	0	0	0	0	0	0	0	0	0	0	0	0	0	0	0	0
PRG4	10216	symbol	H. sapiens	10216	H. sapiens	PRG4	proteoglyca	GO:0006897 endocytos	Disease related genes; Predicted se			(M5882)NABA PROTEO		0	0	0	0	0	0	0	0	0	0	0	0	0	0	0	0	0	0	0	0
PARP15	165631	symbol	H. sapiens	165631	H. sapiens	PARP15	poly(ADP-ri	GO:0070212 protein po	Predicted ir	Mitochondr	N~2~,N~2~-DIMETHYL-N~1~-(6-OX			0	0	0	0	0	0	0	0	0	0	0	0	0	0	0	0	0	0	0	0
ITIH2	3698	symbol	H. sapiens	3698	H. sapiens	ITIH2	inter-alpha-	GO:0030212 hyalurona	Predicted se	Golgi appar	Zinc; Coppe	(M3468)NABA ECM REC		0	0	0	0	0	1	1	0	0	0	0	0	0	0	0	0	0	0	0	0

PAX3	5077	symbol	H. sapiens	5077	H. sapiens	PAX3	paired box :	GO:0007605 sensory pe	Human dise Nucleoplasm (Enhance((M279)PID I (M5956)HA	0	0	1	0	0	0	0	0	0	0	0	0	0	0	0	0	0	0	0	0
GPR78	27201	symbol	H. sapiens	27201	H. sapiens	GPR78	G protein-c	GO:0007189 adenylate	G-protein coupled receptors:GPCRs excl olfactory receptors	0	0	0	0	0	0	0	0	0	0	0	0	0	0	0	0	0	0	0	0
TACR3	6870	symbol	H. sapiens	6870	H. sapiens	TACR3	tachykinin r	GO:0070474 positive re	Disease related genes; (Osanetant; Talnetant; P (M5941)HA	0	0	0	1	0	0	0	0	0	1	0	0	0	0	0	0	0	0	0	0
COL1A1	1277	symbol	H. sapiens	1277	H. sapiens	COL1A1	collagen typ	GO:0071306 cellular res	Human dise Endoplasmi Collagenase (M274)PID I (M5942)HA	0	0	0	0	1	1	1	0	0	0	1	0	0	0	0	0	0	0	0	1
LIPI	149998	symbol	H. sapiens	149998	H. sapiens	LIPI	lipase I	GO:0019433 triglycerid	Disease rela Plasma membrane (Supported)	0	0	0	0	0	0	0	0	0	0	0	0	0	0	0	0	0	0	0	0
BRINP2	57795	symbol	H. sapiens	57795	H. sapiens	BRINP2	BMP/retino	GO:0071300 cellular res	Predicted secreted proteins; Transp (M5883)NABA SECRETE	0	0	0	0	0	1	0	0	0	0	0	0	0	0	0	0	0	0	0	0
LRRC55	219527	symbol	H. sapiens	219527	H. sapiens	LRRC55	leucine rich	GO:1903818 positive re	Transporters:Accessory Factors Involved in Transport	0	1	0	0	0	0	0	0	0	1	0	0	0	0	0	0	0	0	0	0
NFKBIZ	64332	symbol	H. sapiens	64332	H. sapiens	NFKBIZ	NFKB inhibi	GO:2000321 positive re	Transcriptic Nuclear speckles (Supported); Addi (M5947)HA	0	0	0	0	0	0	0	0	0	0	0	0	0	0	0	0	0	0	0	1
GPR173	54328	symbol	H. sapiens	54328	H. sapiens	GPR173	G protein-c	GO:0097210 response t	G-protein c Cytosol (Approved); Additional: Plasma membr	0	0	0	0	1	0	0	0	0	0	0	0	0	0	0	0	0	0	0	0
VAMP1	6843	symbol	H. sapiens	6843	H. sapiens	VAMP1	vesicle asso	GO:0035493 SNARE cor	Human disease related Botulinum Toxin Type B	0	0	0	0	0	0	0	0	0	0	0	0	0	0	0	0	0	0	0	0
GAD1	2571	symbol	H. sapiens	2571	H. sapiens	GAD1	glutamate c	GO:0018352 protein-py	Human dise Cytosol;Plas Pyridoxal phosphate; Gl (M5951)HA	1	0	0	0	0	0	0	0	0	0	0	1	0	0	0	0	0	1	0	0
DGKI	9162	symbol	H. sapiens	9162	H. sapiens	DGKI	diacylglycer	GO:0046834 lipid phos	ENZYME proteins:Transferases; Predicted intracellular prot	0	0	0	1	0	0	1	0	0	0	0	1	0	0	0	0	0	0	0	0
TRIM74	378108	symbol	H. sapiens	378108	H. sapiens	TRIM74	tripartite m	GO:0016567 protein ub	Predicted ir Cytosol (Approved)	0	0	0	0	0	0	0	0	0	0	0	0	0	0	0	0	0	0	0	0
RAC3	5881	symbol	H. sapiens	5881	H. sapiens	RAC3	Rac family s	GO:0021894 cerebral c	RAS pathway related proteins; Predicted intracellular prote	1	0	0	0	0	0	1	1	0	0	0	0	0	0	0	0	0	0	0	0
PLXNB3	5365	symbol	H. sapiens	5365	H. sapiens	PLXNB3	plexin B3	GO:0010593 negative r	Predicted intracellular proteins (M5880)NABA ECM AFF	0	0	0	0	0	1	0	0	0	0	0	0	0	1	0	0	0	0	0	0
TMPRSS11F	389208	symbol	H. sapiens	389208	H. sapiens	TMPRSS11F	transmemb	GO:0033561 regulation	Peptidases:Serine-type peptidases; Enzymes	0	0	0	0	0	0	1	1	0	0	0	0	0	0	0	0	0	0	0	0
CELSR3	1951	symbol	H. sapiens	1951	H. sapiens	CELSR3	cadherin EG	GO:0060071 Wnt signal	G-protein coupled receptors:GPCRs excl olfactory receptors	0	0	0	0	0	0	0	0	0	0	0	0	0	1	0	0	0	0	0	0
TUBB3	10381	symbol	H. sapiens	10381	H. sapiens	TUBB3	tubulin beta	GO:1990791 dorsal roo	Human dise Microtubule Epothilone D; Patupilone; Ixabepilo	1	0	0	0	0	0	1	0	0	0	0	0	0	0	0	0	0	0	0	0
PATE1	160065	symbol	H. sapiens	160065	H. sapiens	PATE1	prostate an	GO:0099601 regulation	Predicted secreted proteins	0	0	0	1	0	0	0	0	0	0	0	0	0	0	0	0	0	0	0	0
YBX2	51087	symbol	H. sapiens	51087	H. sapiens	YBX2	Y-box bindi	GO:0009386 translatior	Transcription factors:beta-Barrel DNA-binding ((M5951)HA	0	0	0	0	0	0	0	0	0	0	0	0	0	0	0	0	0	0	0	0
SAPCD1	401251	symbol	H. sapiens	401251	H. sapiens	SAPCD1	suppressor APC domain containing		Predicted ir Vesicles (Approved); Additional: Cell Junctions	0	0	0	0	0	0	0	0	0	0	0	0	0	0	0	0	0	0	0	0
UNC13C	440279	symbol	H. sapiens	440279	H. sapiens	UNC13C	unc-13 hom	GO:0031914 negative r	Predicted intracellular proteins	1	0	0	0	0	0	0	0	0	0	0	1	0	1	1	0	0	0	0	0
ZNF366	167465	symbol	H. sapiens	167465	H. sapiens	ZNF366	zinc finger p	GO:0033147 negative r	Transcriptic Nucleoplasm (Supported); Additional: Plasma m	0	0	0	0	0	0	0	0	0	0	0	0	0	0	0	0	0	0	0	0
GRHL3	57822	symbol	H. sapiens	57822	H. sapiens	GRHL3	grainyhead	GO:0090179 planar cell	Disease rela Nucleoplasm (Approved)	0	0	0	0	0	0	1	1	0	0	1	0	0	0	0	0	0	0	0	0
SHOX2	6474	symbol	H. sapiens	6474	H. sapiens	SHOX2	short statur	GO:0003172 sinoatrial	Transcription factors:Helix-turn-helix domains; (M5941)HA	0	0	1	0	0	0	0	0	0	1	0	0	0	1	0	0	0	0	0	1
FLRT3	23767	symbol	H. sapiens	23767	H. sapiens	FLRT3	fibronectin	GO:0003345 proepicar	Disease rela Plasma membrane (Supported); Additional: Cel	0	0	0	0	0	0	0	0	0	0	0	0	0	1	0	0	0	0	0	0
STMN2	11075	symbol	H. sapiens	11075	H. sapiens	STMN2	stathmin 2	GO:0031117 positive re	Predicted ir Vesicles (Approved); Additional: Endoplasmic re	0	0	0	0	0	0	0	0	0	0	0	0	0	0	0	0	0	0	0	0
CCDC103	388389	symbol	H. sapiens	388389	H. sapiens	CCDC103	coiled-coil c	GO:0071907 determina	Disease rela Cytosol (Approved)	0	0	0	0	0	0	0	0	0	0	0	0	0	0	0	0	0	0	0	0
SLC5A8	160728	symbol	H. sapiens	160728	H. sapiens	SLC5A8	solute carri	GO:0015730 propanoat	Transporters:Electrochemical Potential-driven transporters	0	1	0	0	0	0	0	0	1	0	0	0	0	0	0	0	0	0	1	0
RTBDN	83546	symbol	H. sapiens	83546	H. sapiens	RTBDN	retbindin	GO:0032218 riboflavin	Predicted se Endoplasmic reticulum (Approved)	0	0	0	0	0	0	0	0	1	0	0	0	1	0	0	0	0	0	0	0
CPA4	51200	symbol	H. sapiens	51200	H. sapiens	CPA4	carboxypep	GO:0016573 histone ac	Predicted se Nucleoplasm (Approved); Additional: Centrosor	0	0	0	0	0	0	0	0	0	0	0	0	0	0	0	0	0	0	0	0
REEP2	51308	symbol	H. sapiens	51308	H. sapiens	REEP2	receptor ac	GO:0032596 protein tra	Disease rela Endoplasmic reticulum (Approved)	0	0	0	0	0	0	0	0	0	0	0	0	0	0	0	0	0	0	0	0
PCDHGA9	56107	symbol	H. sapiens	56107	H. sapiens	PCDHGA9	protocadhe	GO:0007156 homophilic cell adhesi	Plasma membrane;Vesicles (Uncertain); Additic	0	0	0	0	0	0	0	0	0	0	0	0	0	1	0	0	0	0	0	0

GroupID	Category	Term	Description	LogP	Log(q-value	InTerm_InL	Genes	Symbols
1_Summary	GO Biologic	GO:009953	trans-synap	-8.26935	-3.921	17/415	1131,1137,	CHRM3,CHRNA4,GABRB3,GAD1,GLRA2,GRM5,HTR1E,HTR3A,NOS1,PLG,PRKCG,RAC3,STX1A,RIMBP2,PRRT2,SYT10,UNC13C,HCN2,KCNJ1,KCNN4,TUBB3,LRRTM2,KCNV1,ASIC1,CNGB1,MLLT11,CLDN19,RNF207
1_Member	GO Biologic	GO:009953	trans-synap	-8.26935	-3.921	17/415	1131,1137,	CHRM3,CHRNA4,GABRB3,GAD1,GLRA2,GRM5,HTR1E,HTR3A,NOS1,PLG,PRKCG,RAC3,STX1A,RIMBP2,PRRT2,SYT10,UNC13C
1_Member	GO Biologic	GO:009953	synaptic sig	-7.83693	-3.790	17/444	1131,1137,	CHRM3,CHRNA4,GABRB3,GAD1,GLRA2,GRM5,HTR1E,HTR3A,NOS1,PLG,PRKCG,RAC3,STX1A,RIMBP2,PRRT2,SYT10,UNC13C
1_Member	GO Biologic	GO:000726	chemical sy	-6.88708	-3.141	15/398	1131,1137,	CHRM3,CHRNA4,GABRB3,GAD1,GLRA2,GRM5,HTR1E,HTR3A,PRKCG,RAC3,STX1A,RIMBP2,PRRT2,SYT10,UNC13C
1_Member	GO Biologic	GO:009891	anterograde	-6.88708	-3.141	15/398	1131,1137,	CHRM3,CHRNA4,GABRB3,GAD1,GLRA2,GRM5,HTR1E,HTR3A,PRKCG,RAC3,STX1A,RIMBP2,PRRT2,SYT10,UNC13C
1_Member	Reactome G	R-HSA-1123	Neuronal Sy	-6.72151	-3.072	15/410	610,1137,2	HCN2,CHRNA4,GABRB3,GAD1,GLRA2,GRM5,HTR3A,KCNJ1,KCNN4,PRKCG,STX1A,TUBB3,LRRTM2,KCNV1,SYT10
1_Member	GO Biologic	GO:004239	regulation c	-5.04059	-1.647	13/426	41,610,113	ASIC1,HCN2,CHRNA4,CNGB1,GABRB3,GLRA2,GRM5,HTR3A,KCNN4,STX1A,MLLT11,CLDN19,RNF207
1_Member	Reactome G	R-HSA-1123	Transmissio	-3.22163	-0.517	8/270	1137,2562,	CHRNA4,GABRB3,GAD1,GLRA2,HTR3A,PRKCG,STX1A,TUBB3
1_Member	Reactome G	R-HSA-1123	Neurotrans	-2.5138	-0.183	6/205	1137,2562,	CHRNA4,GABRB3,GLRA2,HTR3A,PRKCG,TUBB3
2_Summary	GO Biologic	GO:003000	metal ion tr	-5.19552	-1.647	16/615	41,487,610,	ASIC1,ATP2A1,HCN2,CASR,CHRNA4,HPX,KCNJ1,KCNN4,LRP2,SLC12A1,KCNV1,KCNIP1,SLC4A10,SLC5A8,LRRC55,NIPAL4,GABRB3,GLRA2,HTR3A,CNGB1,STRA6
2_Member	GO Biologic	GO:003000	metal ion tr	-5.19552	-1.647	16/615	41,487,610,	ASIC1,ATP2A1,HCN2,CASR,CHRNA4,HPX,KCNJ1,KCNN4,LRP2,SLC12A1,KCNV1,KCNIP1,SLC4A10,SLC5A8,LRRC55,NIPAL4
2_Member	GO Biologic	GO:009866	inorganic io	-4.35049	-1.179	15/644	41,487,610,	ASIC1,ATP2A1,HCN2,CASR,GABRB3,GLRA2,HTR3A,KCNJ1,KCNN4,SLC12A1,KCNV1,KCNIP1,SLC4A10,LRRC55,NIPAL4
2_Member	GO Biologic	GO:007180	potassium i	-3.91188	-0.865	7/159	610,3758,3	HCN2,KCNJ1,KCNN4,SLC12A1,KCNV1,KCNIP1,LRRC55
2_Member	GO Biologic	GO:000681	potassium i	-3.74805	-0.780	7/169	610,3758,3	HCN2,KCNJ1,KCNN4,SLC12A1,KCNV1,KCNIP1,LRRC55
2_Member	GO Biologic	GO:009865	cation trans	-3.24101	-0.518	13/645	41,487,610,	ASIC1,ATP2A1,HCN2,CNGB1,HTR3A,KCNJ1,KCNN4,SLC12A1,KCNV1,KCNIP1,SLC4A10,LRRC55,NIPAL4
2_Member	GO Biologic	GO:009866	inorganic ca	-3.14882	-0.508	12/577	41,487,610,	ASIC1,ATP2A1,HCN2,HTR3A,KCNJ1,KCNN4,SLC12A1,KCNV1,KCNIP1,SLC4A10,LRRC55,NIPAL4
2_Member	GO Biologic	GO:199057	potassium i	-2.43251	-0.156	3/45	610,3758,6	HCN2,KCNJ1,SLC12A1
2_Member	Reactome G	R-HSA-1296	Potassium C	-2.24526	-0.079	4/103	610,3758,3	HCN2,KCNJ1,KCNN4,KCNV1
2_Member	GO Biologic	GO:000681	sodium ion	-2.07924	0.000	5/180	41,610,655	ASIC1,HCN2,SLC12A1,SLC4A10,SLC5A8
2_Member	GO Biologic	GO:009865	import into	-2.00276	0.000	5/188	610,3758,4	HCN2,KCNJ1,LRP2,SLC12A1,STRA6
3_Summary	GO Biologic	GO:006106	muscle stru	-5.12303	-1.647	14/484	1310,1958,	COL19A1,EGR1,FLNC,LRP2,NOS1,PAX3,PITX1,PLG,SHOX2,SPEG,BASP1,FBXO40,STRA6,TRIM72,NPHS2
3_Member	GO Biologic	GO:006106	muscle stru	-5.12303	-1.647	14/484	1310,1958,	COL19A1,EGR1,FLNC,LRP2,NOS1,PAX3,PITX1,PLG,SHOX2,SPEG,BASP1,FBXO40,STRA6,TRIM72
3_Member	GO Biologic	GO:000751	muscle orga	-4.40493	-1.203	10/293	1310,1958,	COL19A1,EGR1,LRP2,PAX3,PITX1,SHOX2,SPEG,BASP1,STRA6,TRIM72
3_Member	GO Biologic	GO:006053	skeletal mu	-2.61882	-0.205	5/134	1310,1958,	COL19A1,EGR1,PITX1,BASP1,STRA6
3_Member	GO Biologic	GO:003283	glomerulus	-2.18745	-0.043	3/55	1958,7827,	EGR1,NPHS2,BASP1
4_Summary	KEGG Pathv	hsa04080	Neuroactive	-5.05065	-1.647	12/362	1131,1137,	CHRM3,CHRNA4,LTB4R,GABRB3,GLRA2,GNRH1,GNRHR,GRM5,HTR1E,PLG,TACR3,PATE1,CASR,DGKI,GPRC5A,CXCL1,CXCL3,CCL20,PRKCG
4_Member	KEGG Pathv	hsa04080	Neuroactive	-5.05065	-1.647	12/362	1131,1137,	CHRM3,CHRNA4,LTB4R,GABRB3,GLRA2,GNRH1,GNRHR,GRM5,HTR1E,PLG,TACR3,PATE1
4_Member	Reactome G	R-HSA-4164	G alpha (q)	-3.87168	-0.846	8/216	846,1131,1	CASR,CHRM3,LTB4R,GNRH1,GNRHR,GRM5,TACR3,DGKI
4_Member	WikiPathwa	WP501	GPCRs, clas	-3.77257	-0.786	3/16	846,2915,9	CASR,GRM5,GPRC5A
4_Member	Reactome G	R-HSA-5007	GPCR ligand	-3.37064	-0.541	11/467	846,1131,1	CASR,CHRM3,LTB4R,GNRH1,GNRHR,GRM5,CXCL1,CXCL3,HTR1E,CCL20,TACR3
4_Member	Reactome G	R-HSA-3883	GPCR down	-3.31688	-0.527	13/633	846,1131,1	CASR,CHRM3,LTB4R,GNRH1,GNRHR,GRM5,CXCL1,CXCL3,HTR1E,PRKCG,CCL20,TACR3,DGKI
4_Member	Reactome G	R-HSA-3730	Class A/1 (R	-3.25165	-0.518	9/335	1131,1241,	CHRM3,LTB4R,GNRH1,GNRHR,CXCL1,CXCL3,HTR1E,CCL20,TACR3
4_Member	Reactome G	R-HSA-3727	Signaling by	-2.87357	-0.406	13/708	846,1131,1	CASR,CHRM3,LTB4R,GNRH1,GNRHR,GRM5,CXCL1,CXCL3,HTR1E,PRKCG,CCL20,TACR3,DGKI
4_Member	GO Biologic	GO:000719	adenylate c	-2.76283	-0.318	4/74	846,1131,2	CASR,CHRM3,GRM5,HTR1E
5_Summary	GO Biologic	GO:190169	cellular resp	-4.98068	-1.633	16/640	366,610,84	AQP9,HCN2,CASR,CHRM3,COL1A1,EGR1,GABRB3,GLRA2,GNRHR,GRM5,HTR1E,HTR3A,IGFBP1,SOCS1,GPR173,IFIT1B,CYP2C9,PRKCG
5_Member	GO Biologic	GO:190169	cellular resp	-4.98068	-1.633	16/640	366,610,84	AQP9,HCN2,CASR,CHRM3,COL1A1,EGR1,GABRB3,GLRA2,GNRHR,GRM5,HTR1E,HTR3A,IGFBP1,SOCS1,GPR173,IFIT1B
5_Member	GO Biologic	GO:007141	cellular resp	-4.87306	-1.566	15/580	366,610,84	AQP9,HCN2,CASR,CHRM3,COL1A1,EGR1,GABRB3,GLRA2,GNRHR,GRM5,HTR1E,HTR3A,IGFBP1,SOCS1,GPR173
5_Member	KEGG Pathv	hsa04726	Serotonergi	-2.91078	-0.432	5/115	1559,2562,	CYP2C9,GABRB3,HTR1E,HTR3A,PRKCG
5_Member	GO Biologic	GO:000721	serotonin re	-2.57879	-0.188	3/40	1131,3354,	CHRM3,HTR1E,HTR3A
5_Member	GO Biologic	GO:007140	cellular resp	-2.5562	-0.183	10/505	366,610,84	AQP9,HCN2,CASR,CHRM3,COL1A1,EGR1,GABRB3,HTR1E,HTR3A,IFIT1B
6_Summary	Canonical P	M5885	NABA MATI	-4.70427	-1.435	17/751	12,2919,29	SERPINA3,CXCL1,CXCL3,HPX,IL11,ITIH2,MMP13,SERPINE1,PLG,PLXNB3,CCL20,SFTPB,TNFSF14,FST,BRINP2,ADAMTS14,FREM2,FOSB,EGR1,FAM83A,COL1A1,PRKCG,EDAR
6_Member	Canonical P	M5885	NABA MATI	-4.70427	-1.435	17/751	12,2919,29	SERPINA3,CXCL1,CXCL3,HPX,IL11,ITIH2,MMP13,SERPINE1,PLG,PLXNB3,CCL20,SFTPB,TNFSF14,FST,BRINP2,ADAMTS14,FREM2
6_Member	KEGG Pathv	hsa04657	IL-17 signali	-3.3068	-0.527	5/94	2354,2919,	FOSB,CXCL1,CXCL3,MMP13,CCL20

6_Member	KEGG Pathv	hsa05323	Rheumatoic	-2.40239	-0.145	4/93	2919,2921,	CXCL1,CXCL3,IL11,CCL20
6_Member	WikiPathwa	WP5115	Network ma	-2.3547	-0.122	6/221	1958,2919,	EGR1,CXCL1,CXCL3,SERPINE1,CCL20,FAM83A
6_Member	KEGG Pathv	hsa04061	Viral protei	-2.29047	-0.091	4/100	2919,2921,	CXCL1,CXCL3,CCL20,TNFSF14
6_Member	KEGG Pathv	hsa05146	Amoebiasis	-2.26016	-0.082	4/102	1277,2919,	COL1A1,CXCL1,CXCL3,PRKCG
6_Member	KEGG Pathv	hsa04064	NF-kappa B	-2.23053	-0.070	4/104	2919,2921,	CXCL1,CXCL3,TNFSF14,EDAR
6_Member	Reactome G	R-HSA-3801	Chemokine	-2.10304	-0.013	3/59	2919,2921,	CXCL1,CXCL3,CCL20
6_Member	Canonical P	M5883	NABA SECR	-2.00111	0.000	7/343	2919,2921,	CXCL1,CXCL3,IL11,CCL20,TNFSF14,FST,BRINP2
7_Summary	WikiPathwa	WP1533	Vitamin B1	-4.48938	-1.255	5/53	12,4036,50	SERPINA3,LRP2,SERPINE1,PLG,CUBN,F3,OCLN,CDKN2A,COL1A1,KLK2,MMP13,NOS1,CLDN19,ATP2A1,PRKCG,DGKI,TUBB3,KIF18B,RAC3,OLFM4,CHRM3,KCNN4,GRHL3,TMPRSS11F,ITIH2,ADAMTS14
7_Member	WikiPathwa	WP1533	Vitamin B1	-4.48938	-1.255	5/53	12,4036,50	SERPINA3,LRP2,SERPINE1,PLG,CUBN
7_Member	WikiPathwa	WP272	Blood clotti	-3.28798	-0.520	3/23	2152,5054,	F3,SERPINE1,PLG
7_Member	GO Biologic	GO:003019	positive reg	-3.03186	-0.455	3/28	2152,5054,	F3,SERPINE1,PLG
7_Member	GO Biologic	GO:190004	positive reg	-3.03186	-0.455	3/28	2152,5054,	F3,SERPINE1,PLG
7_Member	GO Biologic	GO:009030	positive reg	-3.02321	-0.453	4/63	2152,5054,	F3,SERPINE1,PLG,OCLN
7_Member	GO Biologic	GO:005082	positive reg	-2.98663	-0.439	3/29	2152,5054,	F3,SERPINE1,PLG
7_Member	GO Biologic	GO:001081	negative re	-2.94742	-0.439	4/66	1029,1277,	CDKN2A,COL1A1,SERPINE1,PLG
7_Member	Reactome G	R-HSA-1592	Activation c	-2.82132	-0.360	3/33	3817,4322,	KLK2,MMP13,PLG
7_Member	WikiPathwa	WP176	Folate meta	-2.78464	-0.334	4/73	12,4842,50	SERPINA3,NOS1,SERPINE1,PLG
7_Member	GO Biologic	GO:190303	positive reg	-2.69933	-0.276	4/77	2152,5054,	F3,SERPINE1,PLG,OCLN
7_Member	GO Biologic	GO:006104	regulation c	-2.57719	-0.188	5/137	2152,5054,	F3,SERPINE1,PLG,CLDN19,OCLN
7_Member	Canonical P	M174	PID UPA UP	-2.51799	-0.183	3/42	4322,5054,	MMP13,SERPINE1,PLG
7_Member	Reactome G	R-HSA-1095	Hemostasis	-2.39238	-0.145	11/621	12,487,127	SERPINA3,ATP2A1,COL1A1,F3,NOS1,SERPINE1,PLG,PRKCG,DGKI,TUBB3,KIF18B
7_Member	GO Biologic	GO:001081	regulation c	-2.38341	-0.139	6/218	1029,1277,	CDKN2A,COL1A1,SERPINE1,PLG,RAC3,OLFM4
7_Member	GO Biologic	GO:005087	regulation c	-2.35941	-0.122	8/371	1131,2152,	CHRM3,F3,KCNN4,SERPINE1,PLG,DGKI,GRHL3,TMPRSS11F
7_Member	Canonical P	M3468	NABA ECM	-2.20088	-0.054	6/238	12,3698,43	SERPINA3,ITIH2,MMP13,SERPINE1,PLG,ADAMTS14
7_Member	GO Biologic	GO:190303	regulation c	-2.18102	-0.042	5/170	2152,5054,	F3,SERPINE1,PLG,CLDN19,OCLN
7_Member	WikiPathwa	WP558	Complemer	-2.10304	-0.013	3/59	2152,5054,	F3,SERPINE1,PLG
8_Summary	GO Biologic	GO:004887	multicellula	-4.32031	-1.176	11/363	12,1258,19	SERPINA3,CNGB1,EGR1,RAC3,CUBN,RCN3,GRHL3,MFSD2A,USH1G,TMPRSS11F,OCLN,PLG
8_Member	GO Biologic	GO:004887	multicellula	-4.32031	-1.176	11/363	12,1258,19	SERPINA3,CNGB1,EGR1,RAC3,CUBN,RCN3,GRHL3,MFSD2A,USH1G,TMPRSS11F,OCLN
8_Member	GO Biologic	GO:006024	anatomical	-3.8332	-0.828	9/279	12,1258,53	SERPINA3,CNGB1,PLG,RAC3,CUBN,RCN3,MFSD2A,USH1G,OCLN
8_Member	GO Biologic	GO:000189	tissue home	-3.62228	-0.751	8/235	12,1258,58	SERPINA3,CNGB1,RAC3,CUBN,RCN3,MFSD2A,USH1G,OCLN
9_Summary	GO Biologic	GO:000682	anion trans	-4.1386	-1.021	12/449	366,846,25	AQP9,CASR,GABRB3,GLRA2,LRP2,SLC12A1,CLIC5,SLC4A10,RTBDN,MFSD2A,SLC25A21,SLC5A8
9_Member	GO Biologic	GO:000682	anion trans	-4.1386	-1.021	12/449	366,846,25	AQP9,CASR,GABRB3,GLRA2,LRP2,SLC12A1,CLIC5,SLC4A10,RTBDN,MFSD2A,SLC25A21,SLC5A8
9_Member	GO Biologic	GO:000682	chloride tra	-4.08875	-0.996	6/103	846,2562,2	CASR,GABRB3,GLRA2,SLC12A1,CLIC5,SLC4A10
9_Member	GO Biologic	GO:009865	anion trans	-3.66	-0.751	8/232	846,2562,2	CASR,GABRB3,GLRA2,LRP2,SLC12A1,SLC4A10,SLC25A21,SLC5A8
9_Member	GO Biologic	GO:190247	chloride tra	-2.94742	-0.439	4/66	846,2562,2	CASR,GABRB3,GLRA2,SLC12A1
9_Member	GO Biologic	GO:001569	inorganic ar	-2.9357	-0.439	6/169	846,2562,2	CASR,GABRB3,GLRA2,SLC12A1,CLIC5,SLC4A10
9_Member	GO Biologic	GO:001571	organic anic	-2.79306	-0.337	8/315	366,846,40	AQP9,CASR,LRP2,SLC4A10,RTBDN,MFSD2A,SLC25A21,SLC5A8
9_Member	GO Biologic	GO:009866	inorganic ar	-2.48848	-0.174	4/88	846,2562,2	CASR,GABRB3,GLRA2,SLC12A1
10_Summa	GO Biologic	GO:004326	regulation c	-3.9751	-0.906	15/696	487,610,84	ATP2A1,HCN2,CASR,CHRM3,CHRNA4,GRM5,KCNJ1,KCNN4,NOS1,KCNV1,KCNIP1,CLIC5,LRRC55,SYT10,RNF207,OCLN,SHOX2,TACR3,PON1,PRKCG,STX1A,TRIM63
10_Membe	GO Biologic	GO:004326	regulation c	-3.9751	-0.906	15/696	487,610,84	ATP2A1,HCN2,CASR,CHRM3,CHRNA4,GRM5,KCNJ1,KCNN4,NOS1,KCNV1,KCNIP1,CLIC5,LRRC55,SYT10,RNF207
10_Membe	GO Biologic	GO:003476	regulation c	-3.672	-0.751	12/504	487,610,11	ATP2A1,HCN2,CHRM3,GRM5,KCNJ1,KCNN4,NOS1,KCNV1,KCNIP1,CLIC5,LRRC55,RNF207
10_Membe	GO Biologic	GO:003476	regulation c	-3.52263	-0.680	13/602	487,610,11	ATP2A1,HCN2,CHRM3,GRM5,KCNJ1,KCNN4,NOS1,KCNV1,KCNIP1,CLIC5,LRRC55,RNF207,OCLN
10_Membe	GO Biologic	GO:190352	regulation c	-3.34028	-0.527	8/259	487,610,84	ATP2A1,HCN2,CASR,CHRM3,NOS1,SHOX2,TACR3,RNF207
10_Membe	GO Biologic	GO:004326	regulation c	-2.96201	-0.439	5/112	3783,4842,	KCNN4,NOS1,KCNIP1,LRRC55,RNF207

10_Membe	GO Biologic	GO:003240	regulation c	-2.75033	-0.316	8/320	487,610,11	ATP2A1,HCN2,CHRM3,GRM5,NOS1,PON1,LRRC55,RNF207
10_Membe	GO Biologic	GO:003241	regulation c	-2.51123	-0.183	7/275	487,610,11	ATP2A1,HCN2,CHRM3,GRM5,NOS1,LRRC55,RNF207
10_Membe	GO Biologic	GO:000801	regulation c	-2.46245	-0.169	6/210	487,610,48	ATP2A1,HCN2,NOS1,SHOX2,TACR3,RNF207
10_Membe	GO Biologic	GO:190138	positive reg	-2.46029	-0.169	3/44	3783,21952	KCNN4,LRRC55,RNF207
10_Membe	GO Biologic	GO:002289	regulation c	-2.42628	-0.154	7/285	487,610,11	ATP2A1,HCN2,CHRM3,GRM5,NOS1,LRRC55,RNF207
10_Membe	GO Biologic	GO:004593	positive reg	-2.35311	-0.122	3/48	487,1131,6	ATP2A1,CHRM3,TACR3
10_Membe	GO Biologic	GO:190137	regulation c	-2.33729	-0.113	4/97	3783,30820	KCNN4,KCNIP1,LRRC55,RNF207
10_Membe	GO Biologic	GO:003476	positive reg	-2.28959	-0.091	6/228	487,3783,4	ATP2A1,KCNN4,NOS1,LRRC55,RNF207,OCLN
10_Membe	GO Biologic	GO:004326	positive reg	-2.27902	-0.086	3/51	3783,21952	KCNN4,LRRC55,RNF207
10_Membe	GO Biologic	GO:190406	positive reg	-2.24571	-0.079	5/164	487,3783,4	ATP2A1,KCNN4,NOS1,LRRC55,RNF207
10_Membe	KEGG Pathv	hsa04020	Calcium sig	-2.18371	-0.042	6/240	487,1131,2	ATP2A1,CHRM3,GRM5,NOS1,PRKCG,TACR3
10_Membe	GO Biologic	GO:000693	regulation c	-2.14972	-0.032	5/173	487,1131,4	ATP2A1,CHRM3,NOS1,TACR3,RNF207
10_Membe	GO Biologic	GO:004405	regulation c	-2.12593	-0.016	10/583	487,610,84	ATP2A1,HCN2,CASR,CHRM3,NOS1,SHOX2,STX1A,TACR3,TRIM63,RNF207
10_Membe	GO Biologic	GO:003476	positive reg	-2.09902	-0.011	5/178	487,3783,4	ATP2A1,KCNN4,NOS1,LRRC55,RNF207
10_Membe	GO Biologic	GO:009025	regulation c	-2.07645	0.000	6/253	487,1131,4	ATP2A1,CHRM3,NOS1,TACR3,TRIM63,RNF207
11_Summa	GO Biologic	GO:004247	odontogene	-3.70381	-0.754	6/121	1277,5054,	COL1A1,SERPINE1,PITX2,FST,EDAR,LHX8,KRTAP5-9,SOX21,GRHL3,LCE1A
11_Membe	GO Biologic	GO:004247	odontogene	-3.70381	-0.754	6/121	1277,5054,	COL1A1,SERPINE1,PITX2,FST,EDAR,LHX8
11_Membe	GO Biologic	GO:004247	odontogene	-2.63858	-0.220	4/80	5054,10468	SERPINE1,FST,EDAR,LHX8
11_Membe	GO Biologic	GO:000854	epidermis d	-2.28278	-0.087	7/303	3846,5308,	KRTAP5-9,PITX2,FST,EDAR,SOX21,GRHL3,LCE1A
11_Membe	GO Biologic	GO:004358	skin develo	-2.02934	0.000	6/259	1277,10468	COL1A1,FST,EDAR,SOX21,GRHL3,LCE1A
12_Summa	GO Biologic	GO:005080	modulation	-3.63097	-0.751	11/435	41,2915,55	ASIC1,GRM5,PRKCG,STX1A,DGKI,LRRTM2,SLC4A10,CLSTN2,NTNG2,PRRT2,UNC13C,GAD1,NOS1
12_Membe	GO Biologic	GO:005080	modulation	-3.63097	-0.751	11/435	41,2915,55	ASIC1,GRM5,PRKCG,STX1A,DGKI,LRRTM2,SLC4A10,CLSTN2,NTNG2,PRRT2,UNC13C
12_Membe	GO Biologic	GO:009917	regulation c	-3.62245	-0.751	11/436	41,2915,55	ASIC1,GRM5,PRKCG,STX1A,DGKI,LRRTM2,SLC4A10,CLSTN2,NTNG2,PRRT2,UNC13C
12_Membe	GO Biologic	GO:000150	regulation c	-3.16741	-0.508	7/211	41,2571,48	ASIC1,GAD1,NOS1,PRKCG,STX1A,PRRT2,UNC13C
12_Membe	GO Biologic	GO:004816	regulation c	-2.52425	-0.183	6/204	2915,5582,	GRM5,PRKCG,LRRTM2,SLC4A10,PRRT2,UNC13C
12_Membe	GO Biologic	GO:005158	regulation c	-2.30589	-0.091	4/99	41,4842,55	ASIC1,NOS1,PRKCG,STX1A
13_Summa	GO Biologic	GO:005118	vitamin tra	-3.58311	-0.726	4/45	4036,8029,	LRP2,CUBN,STRA6,RTBDN,FST,LHX8
13_Membe	GO Biologic	GO:005118	vitamin tra	-3.58311	-0.726	4/45	4036,8029,	LRP2,CUBN,STRA6,RTBDN
13_Membe	GO Biologic	GO:004666	female sex	-2.05297	0.000	4/117	4036,10468	LRP2,FST,STRA6,LHX8
14_Summa	GO Biologic	GO:009874	cell-cell adh	-3.31825	-0.527	8/261	1951,5365,	CELSR3,PLXNB3,FLRT3,PCDHGA9,CLSTN2,NTNG2,IGFN1,CLDN19,GRM5,LRP2,SHOX2,LRRTM2,GABRB3,UNC13C,OCLN
14_Membe	GO Biologic	GO:009874	cell-cell adh	-3.31825	-0.527	8/261	1951,5365,	CELSR3,PLXNB3,FLRT3,PCDHGA9,CLSTN2,NTNG2,IGFN1,CLDN19
14_Membe	GO Biologic	GO:005196	positive reg	-2.55522	-0.183	7/270	2915,4036,	GRM5,LRP2,PLXNB3,SHOX2,FLRT3,LRRTM2,CLSTN2
14_Membe	GO Biologic	GO:005080	synapse org	-2.39339	-0.145	7/289	2562,2915,	GABRB3,GRM5,FLRT3,LRRTM2,NTNG2,IGFN1,UNC13C
14_Membe	GO Biologic	GO:000741	synapse ass	-2.27522	-0.086	4/101	2562,23767	GABRB3,FLRT3,NTNG2,IGFN1
14_Membe	GO Biologic	GO:005196	regulation c	-2.24526	-0.079	4/103	23767,2604	FLRT3,LRRTM2,CLSTN2,NTNG2
14_Membe	GO Biologic	GO:190189	positive reg	-2.21596	-0.058	4/105	23767,2604	FLRT3,LRRTM2,CLSTN2,CLDN19
14_Membe	GO Biologic	GO:000715	homophilic	-2.21301	-0.058	5/167	1951,5365,	CELSR3,PLXNB3,PCDHGA9,CLSTN2,IGFN1
14_Membe	GO Biologic	GO:003433	cell junctior	-2.20529	-0.054	9/479	2562,2915,	GABRB3,GRM5,FLRT3,LRRTM2,NTNG2,IGFN1,CLDN19,UNC13C,OCLN
14_Membe	GO Biologic	GO:005196	positive reg	-2.02483	0.000	3/63	23767,2604	FLRT3,LRRTM2,CLSTN2
14_Membe	GO Biologic	GO:003432	cell junctior	-2.00632	0.000	6/262	2562,23767	GABRB3,FLRT3,NTNG2,IGFN1,CLDN19,OCLN
15_Summa	Reactome C	R-HSA-6794	Neurexins a	-3.24678	-0.518	4/55	2915,6804,	GRM5,STX1A,LRRTM2,SYT10,AQP9,CASR,CHRM3,KCNN4,RCN3,SYT16,PRRT2,UNC13C,TRIM72,CHRNA4
15_Membe	Reactome C	R-HSA-6794	Neurexins a	-3.24678	-0.518	4/55	2915,6804,	GRM5,STX1A,LRRTM2,SYT10
15_Membe	GO Biologic	GO:004690	secretion	-3.19784	-0.508	11/490	366,846,11	AQP9,CASR,CHRM3,KCNN4,STX1A,RCN3,SYT16,PRRT2,SYT10,UNC13C,TRIM72
15_Membe	Reactome C	R-HSA-6794	Protein-pro	-2.52449	-0.183	4/86	2915,6804,	GRM5,STX1A,LRRTM2,SYT10

15_Membe	GO Biologic	GO:001715	calcium-ion	-2.48877	-0.174	3/43	6804,34135	STX1A,SYT10,UNC13C
15_Membe	GO Biologic	GO:000688	exocytosis	-2.31718	-0.096	6/225	6804,83851	STX1A,SYT16,PRRT2,SYT10,UNC13C,TRIM72
15_Membe	GO Biologic	GO:001607	synaptic ve	-2.16571	-0.035	3/56	6804,11247	STX1A,PRRT2,UNC13C
15_Membe	GO Biologic	GO:005043	regulation c	-2.16571	-0.035	3/56	1137,6804,	CHRNA4,STX1A,SYT10
16_Summa	KEGG Pathv	hsa03015	mRNA surve	-3.24434	-0.518	5/97	5522,8106,	PPP2R2C,PABPN1,PABPC1L,PABPC1L2A,PABPC1L2B
16_Membe	KEGG Pathv	hsa03015	mRNA surve	-3.24434	-0.518	5/97	5522,8106,	PPP2R2C,PABPN1,PABPC1L,PABPC1L2A,PABPC1L2B
17_Summa	GO Biologic	GO:004356	regulation c	-3.23219	-0.518	3/24	3484,8483,	IGFBP1,CILP,TRIM72
17_Membe	GO Biologic	GO:004356	regulation c	-3.23219	-0.518	3/24	3484,8483,	IGFBP1,CILP,TRIM72
18_Summa	GO Biologic	GO:000761	behavior	-3.18168	-0.508	12/572	41,846,113	ASIC1,CASR,CHRNA4,EGR1,GAD1,GRM5,PRKCG,SLC4A10,DMRT3,STRA6,MFSD2A,LHX8
18_Membe	GO Biologic	GO:000761	behavior	-3.18168	-0.508	12/572	41,846,113	ASIC1,CASR,CHRNA4,EGR1,GAD1,GRM5,PRKCG,SLC4A10,DMRT3,STRA6,MFSD2A,LHX8
18_Membe	GO Biologic	GO:005089	cognition	-2.25242	-0.079	7/307	41,1137,29	ASIC1,CHRNA4,GRM5,PRKCG,STRA6,MFSD2A,LHX8
18_Membe	GO Biologic	GO:000762	locomotory	-2.03095	0.000	5/185	1958,2571,	EGR1,GAD1,GRM5,SLC4A10,DMRT3
19_Summa	GO Biologic	GO:004217	xenobiotic c	-3.17887	-0.508	3/25	1544,1559,	CYP1A2,CYP2C9,NOS1,BAAT,EGR1,IGFBP1,IL11,CCL20,SLC5A8,HPX,CUBN,PON1,LRP2
19_Membe	GO Biologic	GO:004217	xenobiotic c	-3.17887	-0.508	3/25	1544,1559,	CYP1A2,CYP2C9,NOS1
19_Membe	WikiPathwa	WP2882	Nuclear rec	-2.73348	-0.304	8/322	570,1544,1	BAAT,CYP1A2,CYP2C9,EGR1,IGFBP1,IL11,CCL20,SLC5A8
19_Membe	GO Biologic	GO:003301	tetrapyrrole	-2.10304	-0.013	3/59	1544,3263,	CYP1A2,HPX,CUBN
19_Membe	Reactome G	R-HSA-2142	Arachidonic	-2.10304	-0.013	3/59	1544,1559,	CYP1A2,CYP2C9,PON1
19_Membe	GO Biologic	GO:000820	steroid met	-2.0685	0.000	6/254	570,1544,1	BAAT,CYP1A2,CYP2C9,LRP2,PON1,CUBN
19_Membe	GO Biologic	GO:000820	cholesterol	-2.04029	0.000	4/118	1544,1559,	CYP1A2,CYP2C9,PON1,CUBN
20_Summa	GO Biologic	GO:003296	collagen me	-3.15894	-0.508	4/58	1277,4322,	COL1A1,MMP13,RCN3,ADAMTS14,CXCL1,FST,NFKBIZ,SHOX2,COL19A1,KLK2,PLG,CASR,EGR1,PITX1,SERPINE1
20_Membe	GO Biologic	GO:003296	collagen me	-3.15894	-0.508	4/58	1277,4322,	COL1A1,MMP13,RCN3,ADAMTS14
20_Membe	WikiPathwa	WP5055	Burn wound	-2.99706	-0.441	5/110	1277,2919,	COL1A1,CXCL1,MMP13,FST,NFKBIZ
20_Membe	GO Biologic	GO:006035	cartilage de	-2.94308	-0.439	3/30	1277,4322,	COL1A1,MMP13,SHOX2
20_Membe	Reactome G	R-HSA-1474	Degradation	-2.53664	-0.183	5/140	1277,1310,	COL1A1,COL19A1,KLK2,MMP13,PLG
20_Membe	Reactome G	R-HSA-1474	Collagen fo	-2.45338	-0.169	4/90	1277,1310,	COL1A1,COL19A1,MMP13,ADAMTS14
20_Membe	GO Biologic	GO:006144	connective	-2.45237	-0.169	6/211	846,1277,1	CASR,COL1A1,EGR1,MMP13,PITX1,SHOX2
20_Membe	Reactome G	R-HSA-1474	Extracellula	-2.29817	-0.091	7/301	1277,1310,	COL1A1,COL19A1,KLK2,MMP13,SERPINE1,PLG,ADAMTS14
20_Membe	GO Biologic	GO:006035	endochondr	-2.16571	-0.035	3/56	1277,4322,	COL1A1,MMP13,SHOX2
20_Membe	Reactome G	R-HSA-1442	Collagen de	-2.00615	0.000	3/64	1277,1310,	COL1A1,COL19A1,MMP13
