# Supplementary material for: Construction and Validation of a Novel Cuproptosis-Related Seven-lncRNA Signature to Predict the Outcomes, Immunotherapeutic Responses, and Targeted Therapy in Patients with Clear Cell Renal Cell Carcinoma
Source: Dis Markers. 2023 Jan 25;2023:7219794. doi: 10.1155/2023/7219794 (PMC9893525; doi:10.1155/2023/7219794)
Supplement: Supplementary 10 — Table S5: differentially expressed genes (DEGs) between the high- and low-risk subgroups. [file 7219794.f10.docx]

| gene | lowMean | highMean | logFC | pValue | fdr |
| --- | --- | --- | --- | --- | --- |
| FST | 1.002536 | 2.66644 | 1.411261 | 0.000631 | 0.000983 |
| AC019257.1 | 0.818471 | 1.676512 | 1.03446 | 1.04E-12 | 4.15E-12 |
| CCN5 | 0.572412 | 2.63233 | 2.201216 | 1.82E-09 | 5.19E-09 |
| GSDMB | 0.786434 | 2.152677 | 1.452734 | 2.59E-33 | 5.04E-31 |
| SLN | 1.035625 | 2.58744 | 1.321024 | 2.54E-06 | 5.19E-06 |
| AURKB | 0.962635 | 2.30069 | 1.257006 | 2.94E-16 | 1.80E-15 |
| HAVCR1 | 37.84143 | 17.64863 | -1.10041 | 6.34E-16 | 3.71E-15 |
| LINC01786 | 0.729547 | 1.502374 | 1.042171 | 1.45E-20 | 1.56E-19 |
| LINC01554 | 2.393433 | 6.386532 | 1.415951 | 0.012745 | 0.016974 |
| PGGHG | 13.00866 | 38.12691 | 1.551338 | 5.57E-23 | 9.13E-22 |
| TRBJ1-3 | 1.271708 | 2.65448 | 1.061662 | 1.94E-07 | 4.43E-07 |
| DNER | 0.860748 | 2.569267 | 1.577693 | 5.31E-05 | 9.36E-05 |
| FREM2 | 2.273631 | 1.083118 | -1.06981 | 2.35E-23 | 4.12E-22 |
| PTGES | 2.794283 | 7.413355 | 1.407649 | 3.27E-08 | 8.15E-08 |
| SYT9 | 2.661516 | 1.295081 | -1.03921 | 7.21E-13 | 2.93E-12 |
| AQP9 | 2.646655 | 5.547 | 1.067537 | 0.000437 | 0.000695 |
| F2 | 1.340067 | 4.181351 | 1.641664 | 0.000114 | 0.000194 |
| AP001052.1 | 1.083763 | 2.194108 | 1.017585 | 2.36E-14 | 1.14E-13 |
| TRBJ2-7 | 1.251723 | 2.708163 | 1.113399 | 3.87E-09 | 1.07E-08 |
| MTND2P28 | 185.1249 | 375.0726 | 1.018671 | 3.76E-16 | 2.27E-15 |
| SNHG12 | 2.122376 | 4.807296 | 1.179546 | 1.68E-28 | 8.80E-27 |
| AP006621.2 | 1.159776 | 2.76607 | 1.253991 | 8.87E-23 | 1.40E-21 |
| AMY2B | 0.849911 | 1.822033 | 1.100164 | 4.06E-17 | 2.79E-16 |
| AL021707.6 | 1.16349 | 2.990176 | 1.361771 | 3.02E-28 | 1.48E-26 |
| LINC02604 | 1.631745 | 4.078452 | 1.321606 | 2.22E-36 | 1.19E-33 |
| IGHG2 | 60.29582 | 160.491 | 1.412362 | 0.01333 | 0.017712 |
| AHSA2P | 3.071707 | 6.910437 | 1.169736 | 1.46E-26 | 4.73E-25 |
| ADAM8 | 1.701193 | 3.702718 | 1.122038 | 2.92E-20 | 3.02E-19 |
| HSD17B7P2 | 0.802828 | 1.686415 | 1.070797 | 8.62E-24 | 1.66E-22 |
| BCYRN1 | 2.397632 | 5.004258 | 1.061546 | 3.87E-07 | 8.57E-07 |
| AC105020.1 | 0.887112 | 1.818481 | 1.035546 | 3.97E-16 | 2.38E-15 |
| MTND1P23 | 43.13257 | 108.4343 | 1.329972 | 2.86E-12 | 1.09E-11 |
| MOCOS | 0.893164 | 1.915555 | 1.100766 | 1.08E-08 | 2.84E-08 |
| IGKC | 1652.193 | 4207.21 | 1.348482 | 0.001002 | 0.001526 |
| GHR | 4.317499 | 2.093927 | -1.04398 | 2.94E-22 | 4.20E-21 |
| AL359704.2 | 7.867107 | 3.854152 | -1.02942 | 1.18E-05 | 2.24E-05 |
| AL928654.2 | 1.51298 | 3.108988 | 1.039052 | 2.45E-20 | 2.57E-19 |
| ADH1C | 0.395343 | 2.798762 | 2.823612 | 0.003457 | 0.004933 |
| C1QL1 | 12.9703 | 36.58904 | 1.4962 | 1.56E-09 | 4.50E-09 |
| SNTG2-AS1 | 0.365756 | 2.559207 | 2.806744 | 1.13E-13 | 5.08E-13 |
| OLIG1 | 0.785229 | 1.671206 | 1.089704 | 6.31E-05 | 0.00011 |
| TNNT3 | 0.398326 | 2.179087 | 2.451704 | 4.13E-05 | 7.39E-05 |
| GADD45G | 3.87097 | 8.657126 | 1.161193 | 1.20E-12 | 4.77E-12 |
| CXCL13 | 2.891307 | 8.212196 | 1.506046 | 1.80E-09 | 5.15E-09 |
| AC011445.1 | 0.66966 | 1.541788 | 1.203103 | 1.17E-22 | 1.81E-21 |
| CXCL2 | 5.712858 | 12.35043 | 1.112277 | 1.57E-07 | 3.63E-07 |
| ATAD3B | 0.972099 | 2.206016 | 1.182268 | 1.41E-32 | 2.06E-30 |
| LINC02783 | 0.537734 | 1.665521 | 1.631008 | 4.87E-09 | 1.33E-08 |
| CPNE7 | 0.480007 | 2.239509 | 2.222054 | 8.12E-24 | 1.57E-22 |
| AC018638.5 | 5.690101 | 12.25418 | 1.106748 | 1.31E-35 | 5.52E-33 |
| GLI4 | 1.539417 | 3.184955 | 1.048889 | 5.21E-33 | 9.05E-31 |
| MIR3682 | 1.796439 | 3.767625 | 1.068516 | 1.15E-20 | 1.26E-19 |
| SLC18A3 | 0.411568 | 2.394236 | 2.540362 | 0.039844 | 0.049769 |
| IGLC3 | 121.2546 | 254.0651 | 1.067159 | 0.002083 | 0.003052 |
| MIR3685 | 3.026012 | 6.607503 | 1.126687 | 3.31E-22 | 4.68E-21 |
| OCLN | 1.687081 | 0.817515 | -1.04521 | 4.93E-16 | 2.93E-15 |
| LINC01436 | 0.779047 | 2.410404 | 1.629493 | 3.28E-09 | 9.13E-09 |
| CHTF18 | 0.953007 | 2.033814 | 1.093629 | 2.64E-30 | 2.03E-28 |
| AC018752.1 | 1.94792 | 0.916606 | -1.08756 | 6.80E-32 | 7.58E-30 |
| AC023669.1 | 2.540656 | 1.098908 | -1.20913 | 0.028614 | 0.036451 |
| FLNC | 1.390029 | 4.091946 | 1.557672 | 0.01189 | 0.015921 |
| FER1L4 | 1.381097 | 3.998018 | 1.53347 | 2.48E-26 | 7.72E-25 |
| CCL20 | 10.20864 | 23.10269 | 1.178271 | 0.002007 | 0.002945 |
| LINC01355 | 0.635378 | 1.539091 | 1.276391 | 4.05E-30 | 3.00E-28 |
| MS4A14 | 0.750808 | 1.594262 | 1.086373 | 1.74E-18 | 1.41E-17 |
| PLA2G2D | 0.680757 | 1.895868 | 1.477648 | 0.000197 | 0.000326 |
| GFPT2 | 1.829453 | 4.869996 | 1.412508 | 4.50E-10 | 1.37E-09 |
| AC139100.2 | 0.603815 | 1.491709 | 1.304787 | 1.20E-25 | 3.22E-24 |
| AL022322.1 | 0.944963 | 1.990776 | 1.075001 | 3.09E-18 | 2.43E-17 |
| KLK4 | 1.987146 | 3.996973 | 1.00821 | 3.29E-09 | 9.16E-09 |
| ARHGAP33 | 1.507646 | 3.179282 | 1.076403 | 5.23E-22 | 7.19E-21 |
| EME2 | 0.935011 | 2.310243 | 1.304989 | 5.08E-27 | 1.83E-25 |
| LRP2 | 50.74849 | 21.75384 | -1.22209 | 7.04E-25 | 1.64E-23 |
| C4BPA | 0.447376 | 1.736258 | 1.956421 | 7.25E-06 | 1.40E-05 |
| AC025171.4 | 0.640528 | 1.428765 | 1.157436 | 2.15E-25 | 5.49E-24 |
| AC084018.1 | 2.001361 | 4.092394 | 1.031964 | 9.12E-20 | 8.70E-19 |
| AC023669.2 | 2.797785 | 1.182693 | -1.24221 | 0.014031 | 0.018586 |
| PBX4 | 0.735672 | 1.546355 | 1.071736 | 2.30E-22 | 3.39E-21 |
| ANO9 | 2.289054 | 4.777947 | 1.061639 | 1.78E-09 | 5.08E-09 |
| NAPSA | 5.781985 | 2.762326 | -1.06568 | 6.45E-10 | 1.93E-09 |
| ANGPTL8 | 1.162847 | 5.56454 | 2.258601 | 6.83E-19 | 5.77E-18 |
| LMNTD2 | 1.389972 | 3.125563 | 1.16906 | 8.83E-26 | 2.44E-24 |
| AC084117.1 | 1.347565 | 3.36271 | 1.31927 | 6.44E-27 | 2.24E-25 |
| AC132872.3 | 1.16998 | 2.77078 | 1.243808 | 1.22E-24 | 2.73E-23 |
| TRBJ1-4 | 1.448414 | 3.333575 | 1.202597 | 3.94E-09 | 1.08E-08 |
| CFTR | 1.135021 | 2.521278 | 1.151436 | 7.64E-05 | 0.000132 |
| AC103810.3 | 1.667871 | 3.63993 | 1.125903 | 2.42E-18 | 1.93E-17 |
| C16orf74 | 1.989741 | 4.345049 | 1.126792 | 6.57E-06 | 1.28E-05 |
| CYSRT1 | 0.77932 | 2.304028 | 1.563871 | 3.11E-16 | 1.90E-15 |
| AC103702.2 | 1.261482 | 3.024152 | 1.261411 | 1.24E-09 | 3.60E-09 |
| PTTG1 | 2.749246 | 5.542698 | 1.011552 | 7.93E-14 | 3.63E-13 |
| AC110285.2 | 1.114055 | 2.780311 | 1.319426 | 3.40E-20 | 3.47E-19 |
| MIR155HG | 1.480419 | 3.960512 | 1.419682 | 3.52E-20 | 3.59E-19 |
| MYH8 | 1.524299 | 0.644065 | -1.24287 | 0.000175 | 0.000292 |
| PKP3 | 0.543748 | 2.15299 | 1.98533 | 3.67E-11 | 1.24E-10 |
| SNORD14A | 2.434165 | 5.315736 | 1.126843 | 4.38E-25 | 1.05E-23 |
| AP005233.2 | 2.229712 | 5.987967 | 1.425209 | 4.19E-09 | 1.15E-08 |
| IGHG1 | 464.8176 | 962.039 | 1.049431 | 0.010607 | 0.014295 |
| AGER | 2.097462 | 4.943919 | 1.237011 | 1.63E-26 | 5.25E-25 |
| HSPG2 | 48.30178 | 20.97197 | -1.20361 | 3.96E-27 | 1.46E-25 |
| AGAP9 | 1.090396 | 2.323489 | 1.09144 | 6.03E-21 | 6.94E-20 |
| MELTF | 0.786278 | 1.607262 | 1.031494 | 4.85E-10 | 1.47E-09 |
| APOH | 1.132326 | 18.45256 | 4.02646 | 0.001969 | 0.002891 |
| LIX1 | 2.38832 | 1.005414 | -1.24821 | 0.00435 | 0.006138 |
| EGR1 | 229.2505 | 107.4985 | -1.09261 | 5.69E-10 | 1.71E-09 |
| AL513320.1 | 0.506532 | 1.592983 | 1.653004 | 2.28E-29 | 1.41E-27 |
| MIR4489 | 0.544532 | 1.567551 | 1.525422 | 3.14E-25 | 7.77E-24 |
| IRF9 | 0.67162 | 1.625643 | 1.275293 | 3.79E-26 | 1.13E-24 |
| AC156455.1 | 1.364573 | 2.859175 | 1.06715 | 3.55E-14 | 1.69E-13 |
| IGLV3-10 | 21.66325 | 59.48368 | 1.457244 | 0.025873 | 0.03317 |
| APCDD1L | 0.628694 | 2.129639 | 1.760178 | 0.010992 | 0.014786 |
| AC004863.1 | 1.040454 | 2.317707 | 1.155486 | 4.70E-14 | 2.22E-13 |
| TRBJ1-6 | 2.614566 | 5.829836 | 1.156884 | 6.46E-10 | 1.93E-09 |
| C10orf99 | 11.47463 | 38.39384 | 1.742427 | 0.005036 | 0.007036 |
| GPRC5A | 2.211811 | 4.912913 | 1.15135 | 1.21E-06 | 2.54E-06 |
| SFTPB | 0.503933 | 2.170326 | 2.106608 | 4.61E-15 | 2.42E-14 |
| MSTO2P | 1.099833 | 2.274198 | 1.048073 | 7.39E-23 | 1.18E-21 |
| WFDC5 | 0.170792 | 2.167502 | 3.665718 | 7.56E-06 | 1.46E-05 |
| AC133644.1 | 0.928993 | 2.520067 | 1.439722 | 2.35E-09 | 6.63E-09 |
| AL035661.1 | 1.62319 | 3.301014 | 1.024078 | 7.85E-05 | 0.000136 |
| INPP5J | 1.36044 | 4.597637 | 1.75682 | 0.002587 | 0.003746 |
| FAM193B | 5.618701 | 13.01177 | 1.211509 | 9.81E-30 | 6.62E-28 |
| NSUN5P1 | 1.187969 | 3.367349 | 1.503116 | 4.22E-38 | 3.67E-35 |
| AC127502.2 | 1.569672 | 3.155133 | 1.007238 | 2.35E-25 | 5.95E-24 |
| IGKV6D-21 | 1.21897 | 2.848509 | 1.224544 | 0.00514 | 0.007172 |
| SAA2 | 1.639301 | 6.631033 | 2.016153 | 1.66E-08 | 4.29E-08 |
| CXCL1 | 5.146693 | 12.78727 | 1.312991 | 0.000792 | 0.00122 |
| HM13-IT1 | 0.749538 | 1.537104 | 1.036142 | 9.66E-24 | 1.85E-22 |
| AL031727.1 | 1.820656 | 4.097089 | 1.170141 | 9.55E-31 | 8.24E-29 |
| RNU4ATAC18P | 0.959658 | 2.367772 | 1.302937 | 1.20E-28 | 6.45E-27 |
| CCL19 | 5.122122 | 11.36951 | 1.150357 | 2.11E-05 | 3.89E-05 |
| MZB1 | 3.050534 | 7.801941 | 1.354771 | 7.38E-06 | 1.43E-05 |
| CLMP | 0.529898 | 2.067572 | 1.964151 | 7.10E-05 | 0.000123 |
| PLA2G2A | 0.450485 | 1.849379 | 2.03749 | 4.95E-07 | 1.08E-06 |
| NPY6R | 6.810849 | 3.401378 | -1.00172 | 6.56E-21 | 7.50E-20 |
| IGLC7 | 3.438983 | 10.4758 | 1.607006 | 0.001828 | 0.002696 |
| PARD3-AS1 | 0.77236 | 1.725888 | 1.159994 | 8.18E-20 | 7.87E-19 |
| MINCR | 0.928786 | 1.934399 | 1.058467 | 9.85E-33 | 1.53E-30 |
| PDCL3P4 | 1.249689 | 2.855822 | 1.192337 | 4.32E-18 | 3.35E-17 |
| PSTPIP1 | 1.311327 | 2.66157 | 1.02125 | 1.66E-16 | 1.05E-15 |
| IGHGP | 42.2957 | 160.4403 | 1.923454 | 0.000419 | 0.000668 |
| KLRA1P | 0.78385 | 1.584546 | 1.015419 | 5.41E-23 | 8.90E-22 |
| PAIP2B | 3.824172 | 1.895558 | -1.01252 | 3.46E-27 | 1.30E-25 |
| HOXB-AS1 | 0.904737 | 1.827178 | 1.014048 | 6.10E-24 | 1.21E-22 |
| ORM2 | 0.106796 | 6.393912 | 5.903763 | 1.86E-15 | 1.03E-14 |
| AC015722.2 | 3.737205 | 8.908299 | 1.25319 | 6.37E-05 | 0.000111 |
| AC010542.5 | 0.758062 | 1.651163 | 1.123094 | 3.07E-31 | 2.98E-29 |
| CRYGS | 0.858771 | 2.518723 | 1.552347 | 5.89E-35 | 2.07E-32 |
| VTN | 2.350723 | 4.994236 | 1.087159 | 0.000453 | 0.000719 |
| MYBL2 | 1.773389 | 3.885263 | 1.131503 | 5.77E-08 | 1.39E-07 |
| AL161669.2 | 1.111252 | 2.425418 | 1.126047 | 6.52E-06 | 1.27E-05 |
| SERPIND1 | 0.464227 | 2.511865 | 2.435858 | 0.002101 | 0.003074 |
| VIL1 | 5.218449 | 2.575409 | -1.01882 | 1.83E-12 | 7.10E-12 |
| IGF2BP2 | 0.881747 | 1.892281 | 1.101689 | 7.06E-07 | 1.52E-06 |
| G6PC | 3.162543 | 1.576264 | -1.00458 | 5.57E-14 | 2.60E-13 |
| AC011472.1 | 1.531876 | 3.065052 | 1.000612 | 2.23E-18 | 1.79E-17 |
| MAT1A | 0.668741 | 1.72837 | 1.369892 | 7.09E-06 | 1.37E-05 |
| CENATAC | 2.179159 | 4.75406 | 1.125389 | 4.48E-30 | 3.28E-28 |
| PILRB | 0.650961 | 1.696808 | 1.38218 | 9.20E-19 | 7.65E-18 |
| AL079338.1 | 1.079415 | 3.552667 | 1.718653 | 0.00325 | 0.004657 |
| AL355488.1 | 1.15636 | 2.645208 | 1.193791 | 2.27E-29 | 1.41E-27 |
| AL354836.1 | 2.196934 | 5.114251 | 1.219031 | 5.43E-22 | 7.45E-21 |
| P3H3 | 2.096401 | 4.718778 | 1.170499 | 0.000636 | 0.000991 |
| MTND4P12 | 36.20411 | 77.92823 | 1.105992 | 1.19E-05 | 2.25E-05 |
| AC116914.2 | 0.9155 | 2.089968 | 1.190849 | 2.99E-30 | 2.26E-28 |
| GOLGA8A | 2.130349 | 6.227935 | 1.547664 | 3.14E-23 | 5.38E-22 |
| TNFRSF25 | 1.447042 | 3.092955 | 1.095879 | 7.63E-21 | 8.64E-20 |
| AKR1B10 | 4.923821 | 10.88743 | 1.144814 | 8.73E-07 | 1.86E-06 |
| C1QTNF12 | 0.989107 | 2.083244 | 1.074633 | 4.01E-13 | 1.68E-12 |
| RNY3P16 | 0.856248 | 2.132962 | 1.316757 | 9.97E-19 | 8.26E-18 |
| MIR6819 | 0.661107 | 1.71646 | 1.376479 | 8.36E-20 | 8.00E-19 |
| AC005899.6 | 0.794616 | 1.633635 | 1.039756 | 1.07E-21 | 1.40E-20 |
| IL6 | 2.568322 | 5.55689 | 1.113452 | 0.001114 | 0.001687 |
| RHPN1 | 2.884613 | 6.121196 | 1.085436 | 5.62E-24 | 1.12E-22 |
| MIR4768 | 2.661659 | 10.44846 | 1.972892 | 9.32E-28 | 4.03E-26 |
| HOTAIRM1 | 0.618275 | 1.50723 | 1.285579 | 5.45E-27 | 1.95E-25 |
| AC009084.2 | 1.106353 | 2.900084 | 1.390283 | 8.54E-11 | 2.79E-10 |
| RNA5SP383 | 1.088777 | 2.757036 | 1.34041 | 5.75E-27 | 2.05E-25 |
| IGLV6-57 | 20.18616 | 56.37665 | 1.481731 | 0.029273 | 0.037203 |
| PLG | 4.448951 | 1.915082 | -1.21606 | 5.81E-07 | 1.26E-06 |
| MFSD2A | 0.800161 | 2.657838 | 1.731892 | 1.27E-12 | 5.01E-12 |
| SYTL1 | 0.729406 | 1.686499 | 1.209238 | 8.95E-23 | 1.41E-21 |
| IGKJ4 | 2.076748 | 5.878349 | 1.501085 | 0.001224 | 0.001843 |
| MISP | 1.204265 | 2.709419 | 1.169831 | 1.22E-05 | 2.31E-05 |
| HP | 6.909166 | 42.22286 | 2.611441 | 3.65E-05 | 6.56E-05 |
| IGLV9-49 | 8.633924 | 45.49105 | 2.397494 | 0.024549 | 0.031575 |
| IGFBP1 | 7.309168 | 38.49348 | 2.396835 | 6.49E-05 | 0.000113 |
| ITIH3 | 0.633019 | 2.610485 | 2.043997 | 2.67E-05 | 4.87E-05 |
| SYCE1L | 1.179949 | 2.578971 | 1.128071 | 2.42E-17 | 1.70E-16 |
| HSPA7 | 2.841711 | 7.184444 | 1.338117 | 5.44E-19 | 4.66E-18 |
| DOC2A | 3.295781 | 7.196275 | 1.12663 | 1.84E-08 | 4.72E-08 |
| GABBR1 | 1.843278 | 3.692794 | 1.002439 | 9.39E-21 | 1.05E-19 |
| GNRH1 | 0.786711 | 1.937332 | 1.300165 | 9.23E-30 | 6.26E-28 |
| APOC3 | 0.377193 | 22.25796 | 5.882873 | 0.001145 | 0.00173 |
| AC004148.1 | 0.985809 | 2.173658 | 1.140744 | 2.59E-25 | 6.51E-24 |
| SNORD14E | 1.202507 | 6.165343 | 2.358136 | 2.10E-27 | 8.31E-26 |
| GTF2IP20 | 1.092593 | 2.218278 | 1.021684 | 6.50E-25 | 1.53E-23 |
| SNHG3 | 1.497063 | 3.335535 | 1.155784 | 3.48E-29 | 2.10E-27 |
| TRBJ1-5 | 2.06809 | 4.378075 | 1.081998 | 6.72E-07 | 1.45E-06 |
| TRBJ1-2 | 0.80157 | 1.665427 | 1.054991 | 2.88E-06 | 5.83E-06 |
| LBP | 16.40302 | 50.25033 | 1.615172 | 0.001167 | 0.001762 |
| RNF207 | 1.204285 | 2.668213 | 1.147697 | 1.51E-23 | 2.77E-22 |
| LUC7L | 3.958555 | 8.004776 | 1.015887 | 7.44E-32 | 8.08E-30 |
| C4orf48 | 1.510582 | 4.030437 | 1.415832 | 2.21E-15 | 1.21E-14 |
| SERPINC1 | 0.159096 | 5.898436 | 5.212365 | 0.00041 | 0.000654 |
| LTB4R | 2.109061 | 4.310395 | 1.031219 | 4.97E-29 | 2.87E-27 |
| MMP12 | 0.323272 | 2.253218 | 2.801166 | 4.12E-09 | 1.13E-08 |
| IGLC2 | 149.6783 | 374.1663 | 1.321815 | 0.000585 | 0.000916 |
| KAT2A | 7.857141 | 16.08153 | 1.033329 | 2.74E-32 | 3.47E-30 |
| AL133351.4 | 0.886734 | 1.934736 | 1.125564 | 2.14E-14 | 1.04E-13 |
| ADIRF-AS1 | 0.791988 | 1.659908 | 1.067553 | 9.16E-21 | 1.02E-19 |
| APOA1 | 0.197391 | 3.715781 | 4.234535 | 5.01E-09 | 1.36E-08 |
| SEC31B | 0.641991 | 1.626512 | 1.341157 | 1.90E-27 | 7.56E-26 |
| AC008610.1 | 1.025491 | 2.872995 | 1.486241 | 1.14E-41 | 4.94E-38 |
| AL162586.1 | 0.733484 | 1.4857 | 1.018305 | 2.56E-20 | 2.67E-19 |
| NDUFA11 | 1.03725 | 2.409731 | 1.216109 | 1.77E-30 | 1.43E-28 |
| AC027458.1 | 2.10441 | 0.702353 | -1.58315 | 9.53E-18 | 7.03E-17 |
| CUBN | 48.70491 | 21.56415 | -1.17543 | 4.57E-21 | 5.38E-20 |
| BASP1 | 7.441762 | 14.89462 | 1.001075 | 5.92E-07 | 1.29E-06 |
| PAQR6 | 0.853458 | 2.205403 | 1.369651 | 2.32E-24 | 4.95E-23 |
| AC002553.1 | 0.814299 | 1.63653 | 1.007009 | 2.30E-27 | 9.01E-26 |
| AC108673.3 | 1.559685 | 3.472025 | 1.154523 | 5.72E-33 | 9.55E-31 |
| AL161669.3 | 1.259955 | 3.396889 | 1.430842 | 1.17E-22 | 1.81E-21 |
| RPL23P2 | 0.695315 | 1.435855 | 1.046172 | 4.58E-27 | 1.66E-25 |
| AL117379.1 | 0.631084 | 1.706812 | 1.4354 | 1.36E-36 | 7.72E-34 |
| MT-TC | 8.897944 | 19.71953 | 1.148081 | 0.008501 | 0.011587 |
| IL1R2 | 4.769777 | 14.16282 | 1.570115 | 0.003961 | 0.00561 |
| TTR | 0.420298 | 4.479974 | 3.414006 | 0.005114 | 0.007141 |
| NPEPL1 | 0.874452 | 2.516793 | 1.525135 | 2.28E-38 | 2.48E-35 |
| RHBG | 0.826114 | 2.427787 | 1.55523 | 1.24E-11 | 4.39E-11 |
| FXYD4 | 1.237532 | 4.264256 | 1.784828 | 0.028376 | 0.036158 |
| RHCG | 9.154196 | 33.29023 | 1.862594 | 1.95E-06 | 4.03E-06 |
| LINC01004 | 0.964572 | 2.527697 | 1.389863 | 1.38E-35 | 5.61E-33 |
| AC020907.4 | 0.625444 | 1.733597 | 1.470815 | 1.08E-27 | 4.63E-26 |
| HMGCS2 | 24.67471 | 11.85707 | -1.05729 | 1.41E-08 | 3.66E-08 |
| SNORA33 | 2.011304 | 5.60777 | 1.479296 | 2.66E-38 | 2.67E-35 |
| CLCNKA | 1.187666 | 2.502697 | 1.075354 | 9.97E-12 | 3.57E-11 |
| AC011462.4 | 1.021602 | 2.566179 | 1.328789 | 5.38E-29 | 3.08E-27 |
| DBH-AS1 | 0.717764 | 1.801921 | 1.327955 | 1.17E-19 | 1.09E-18 |
| F3 | 2.539514 | 6.120092 | 1.269001 | 0.002901 | 0.00418 |
| KMT2E-AS1 | 3.795494 | 8.043528 | 1.083541 | 5.39E-31 | 4.96E-29 |
| LINC00173 | 1.238337 | 2.710643 | 1.130231 | 1.93E-12 | 7.44E-12 |
| SRPX2 | 0.843698 | 2.216149 | 1.393256 | 1.15E-06 | 2.42E-06 |
| MTCO3P12 | 35.8415 | 93.72405 | 1.386788 | 7.77E-09 | 2.07E-08 |
| PPP1R1A | 4.623344 | 10.94537 | 1.243312 | 6.47E-05 | 0.000113 |
| MTRNR2L1 | 0.840639 | 2.013013 | 1.259799 | 4.28E-05 | 7.62E-05 |
| TMEM174 | 13.45309 | 6.378958 | -1.07655 | 6.51E-09 | 1.75E-08 |
| MMP9 | 5.773328 | 16.33701 | 1.500669 | 1.03E-05 | 1.96E-05 |
| AC010201.2 | 0.700655 | 1.423886 | 1.023057 | 4.35E-21 | 5.13E-20 |
| MTCO1P40 | 10.1939 | 28.58304 | 1.487453 | 3.53E-13 | 1.49E-12 |
| HCG27 | 0.908342 | 1.965265 | 1.113416 | 4.04E-15 | 2.13E-14 |
| SCNN1D | 0.657142 | 1.769176 | 1.428801 | 5.40E-27 | 1.94E-25 |
| PLA2G6 | 1.21502 | 2.685235 | 1.144068 | 1.46E-26 | 4.73E-25 |
| PIP | 1.74854 | 4.656048 | 1.412955 | 0.038167 | 0.047803 |
| ITPKA | 0.534873 | 2.654902 | 2.311389 | 2.21E-16 | 1.37E-15 |
| AL713899.1 | 0.848624 | 2.280827 | 1.426359 | 3.66E-19 | 3.21E-18 |
| SLC17A9 | 1.246077 | 3.237759 | 1.377602 | 3.26E-18 | 2.57E-17 |
| S100G | 0.4809 | 2.976164 | 2.629646 | 8.77E-10 | 2.59E-09 |
| CDC20 | 2.252513 | 4.571917 | 1.021264 | 8.58E-08 | 2.04E-07 |
| KL | 26.23398 | 12.70932 | -1.04555 | 1.35E-25 | 3.62E-24 |
| MTRNR2L12 | 1.912558 | 4.321492 | 1.176026 | 1.93E-07 | 4.42E-07 |
| NMU | 0.616981 | 1.606234 | 1.380384 | 1.95E-05 | 3.60E-05 |
| MLLT11 | 0.99728 | 1.99763 | 1.00222 | 7.51E-08 | 1.80E-07 |
| ZNF692 | 3.476183 | 8.220126 | 1.241656 | 1.14E-34 | 3.61E-32 |
| UBE2C | 3.208993 | 9.910226 | 1.626797 | 1.41E-16 | 9.07E-16 |
| AC093001.2 | 1.01141 | 2.055855 | 1.023371 | 1.05E-05 | 1.99E-05 |
| CLIC5 | 2.166027 | 0.959962 | -1.174 | 2.82E-16 | 1.73E-15 |
| CAPS | 1.268323 | 3.188381 | 1.329902 | 6.81E-33 | 1.12E-30 |
| CNFN | 1.254392 | 2.822359 | 1.169913 | 0.00018 | 0.000299 |
| ZAP70 | 1.269585 | 2.828522 | 1.155692 | 3.13E-19 | 2.77E-18 |
| CCNL2 | 9.245128 | 21.44491 | 1.21387 | 2.37E-29 | 1.46E-27 |
| IGHA1 | 217.958 | 441.029 | 1.016824 | 0.005252 | 0.007315 |
| RNU6-418P | 0.714462 | 1.438068 | 1.009202 | 1.43E-13 | 6.33E-13 |
| AC136475.3 | 8.131904 | 19.99264 | 1.297804 | 9.19E-12 | 3.30E-11 |
| SFN | 3.511608 | 9.01302 | 1.359879 | 0.000245 | 0.000401 |
| AC107373.2 | 1.486895 | 3.340678 | 1.167838 | 0.002572 | 0.003727 |
| UCN | 0.752348 | 1.879049 | 1.320531 | 6.12E-32 | 6.87E-30 |
| KIFC2 | 1.601007 | 3.425982 | 1.097538 | 2.47E-27 | 9.56E-26 |
| SNHG25 | 0.636354 | 1.690281 | 1.409362 | 2.42E-22 | 3.55E-21 |
| AC145207.9 | 0.528527 | 1.497447 | 1.502455 | 1.16E-30 | 9.75E-29 |
| MT1X | 6.014632 | 14.18567 | 1.237886 | 8.94E-08 | 2.12E-07 |
| PTOV1-AS2 | 0.946893 | 2.224622 | 1.232286 | 1.76E-26 | 5.62E-25 |
| HERC2P2 | 1.63872 | 3.878253 | 1.242838 | 2.31E-23 | 4.06E-22 |
| PABPN1 | 1.649943 | 3.911242 | 1.24521 | 1.59E-30 | 1.32E-28 |
| GABRE | 2.123992 | 4.41735 | 1.056403 | 6.45E-12 | 2.36E-11 |
| PRR15L | 7.066448 | 3.202833 | -1.14164 | 0.006497 | 0.008958 |
| CYP17A1 | 2.568682 | 0.711566 | -1.85196 | 0.00019 | 0.000315 |
| C8G | 0.527093 | 1.698088 | 1.687782 | 5.33E-23 | 8.78E-22 |
| AL135844.1 | 0.927709 | 2.20906 | 1.251689 | 8.46E-22 | 1.12E-20 |
| PVALB | 8.408765 | 31.65398 | 1.912421 | 0.000398 | 0.000636 |
| ZNF683 | 0.863726 | 1.751065 | 1.019588 | 1.01E-09 | 2.97E-09 |
| SOCS1 | 2.708677 | 5.703731 | 1.074318 | 4.86E-18 | 3.73E-17 |
| AP000763.2 | 9.776073 | 21.96374 | 1.167797 | 2.87E-08 | 7.19E-08 |
| AC027796.4 | 0.568517 | 1.523159 | 1.421792 | 1.02E-28 | 5.58E-27 |
| MSC | 11.13611 | 24.56141 | 1.141149 | 4.48E-11 | 1.51E-10 |
| AL627309.7 | 0.673754 | 2.012765 | 1.578885 | 1.35E-20 | 1.46E-19 |
| IGHJ5 | 1.136721 | 4.169879 | 1.875127 | 5.49E-07 | 1.20E-06 |
| AC010883.1 | 0.626707 | 1.575047 | 1.329532 | 9.70E-29 | 5.36E-27 |
| KCNN4 | 0.595183 | 1.470631 | 1.305031 | 7.41E-17 | 4.94E-16 |
| BNC2-AS1 | 1.502124 | 3.250381 | 1.113604 | 2.75E-15 | 1.49E-14 |
| PADI3 | 0.46132 | 1.856976 | 2.009115 | 1.05E-07 | 2.46E-07 |
| SNORD19C | 0.938116 | 2.066224 | 1.139159 | 2.46E-18 | 1.97E-17 |
| IGHJ3P | 1.463758 | 5.914957 | 2.014691 | 2.73E-05 | 4.96E-05 |
| MAPK15 | 2.477902 | 5.096037 | 1.040256 | 4.14E-10 | 1.26E-09 |
| SERPINF1 | 13.9457 | 28.47507 | 1.029879 | 4.48E-08 | 1.10E-07 |
| RNU6-312P | 0.734121 | 1.479086 | 1.010616 | 5.09E-13 | 2.11E-12 |
| GOLGA8B | 1.530709 | 4.037329 | 1.399201 | 2.45E-26 | 7.66E-25 |
| NPR3 | 36.76146 | 17.59371 | -1.06313 | 1.01E-19 | 9.57E-19 |
| HJURP | 0.734233 | 1.516375 | 1.046318 | 8.54E-11 | 2.79E-10 |
| MST1P2 | 0.826623 | 1.826975 | 1.144156 | 3.15E-14 | 1.50E-13 |
| AC011352.1 | 1.304087 | 2.765708 | 1.084609 | 6.85E-08 | 1.65E-07 |
| REC8 | 1.438899 | 3.007127 | 1.06342 | 2.17E-22 | 3.22E-21 |
| HSD11B1 | 0.967431 | 3.106053 | 1.682852 | 1.49E-05 | 2.78E-05 |
| PDCD1 | 1.837232 | 3.969386 | 1.111382 | 7.82E-09 | 2.08E-08 |
| TNFSF14 | 0.988816 | 2.431063 | 1.297813 | 5.97E-11 | 1.98E-10 |
| CCL21 | 8.890527 | 20.41645 | 1.199391 | 0.000176 | 0.000293 |
| SLC25A25-AS1 | 0.88465 | 1.868955 | 1.079054 | 2.45E-21 | 3.02E-20 |
| PAGE5 | 0.033341 | 2.83976 | 6.412337 | 0.000108 | 0.000184 |
| TF | 0.560323 | 2.307723 | 2.04214 | 3.35E-09 | 9.32E-09 |
| TMEM158 | 0.627294 | 2.380555 | 1.924085 | 1.46E-08 | 3.77E-08 |
| ATG16L2 | 2.439483 | 4.951583 | 1.021314 | 3.78E-22 | 5.31E-21 |
| AIM2 | 0.988667 | 2.061402 | 1.060069 | 2.07E-08 | 5.26E-08 |
| HHATL | 1.488247 | 3.686829 | 1.308766 | 7.81E-05 | 0.000135 |
| LINC01089 | 1.152261 | 2.880702 | 1.321953 | 1.11E-33 | 2.38E-31 |
| MIR503HG | 1.376063 | 2.804584 | 1.02724 | 6.57E-17 | 4.42E-16 |
| AL157931.1 | 5.175842 | 14.95226 | 1.530499 | 2.21E-07 | 5.03E-07 |
| PPDPFL | 2.925448 | 7.424989 | 1.343731 | 0.000194 | 0.000322 |
| AL353763.2 | 0.891654 | 1.914453 | 1.102377 | 2.13E-32 | 2.81E-30 |
| TNFRSF18 | 0.808601 | 1.892902 | 1.2271 | 7.11E-25 | 1.65E-23 |
| PABPC1L | 2.246838 | 7.06604 | 1.653006 | 2.68E-33 | 5.13E-31 |
| SNORD123 | 0.62283 | 1.649356 | 1.404991 | 7.80E-20 | 7.52E-19 |
| AC008735.2 | 1.784955 | 4.127945 | 1.209536 | 2.91E-22 | 4.17E-21 |
| AC068587.2 | 0.691349 | 1.696488 | 1.295065 | 4.02E-13 | 1.69E-12 |
| MIR324 | 0.692669 | 1.547032 | 1.159265 | 1.08E-15 | 6.14E-15 |
| APOA2 | 0.253594 | 3.85798 | 3.927255 | 0.017801 | 0.023299 |
| RNU2-11P | 0.852465 | 2.133807 | 1.323717 | 7.65E-22 | 1.02E-20 |
| MFAP2 | 0.684338 | 1.562715 | 1.191274 | 0.00637 | 0.008795 |
| CTXN3 | 1.843495 | 0.910082 | -1.01837 | 0.000273 | 0.000445 |
| SST | 92.70608 | 27.11498 | -1.77357 | 0.000319 | 0.000516 |
| COA6-AS1 | 1.367897 | 2.753472 | 1.009293 | 6.87E-28 | 3.03E-26 |
| NPIPP1 | 1.140189 | 2.533311 | 1.151751 | 1.92E-31 | 1.94E-29 |
| SCNN1G | 0.658199 | 3.936904 | 2.580465 | 0.00114 | 0.001724 |
| MIR429 | 0.749789 | 1.541458 | 1.03974 | 0.001007 | 0.001533 |
| FOSB | 49.29839 | 19.44396 | -1.34222 | 3.60E-10 | 1.11E-09 |
| MTND4P24 | 1.450613 | 5.05826 | 1.801978 | 3.86E-10 | 1.18E-09 |
| AL844908.1 | 0.81029 | 1.69559 | 1.065278 | 6.28E-05 | 0.00011 |
| MT3 | 6.897544 | 13.88244 | 1.009107 | 0.014899 | 0.019682 |
| PLA2G4F | 0.678613 | 1.429964 | 1.075318 | 2.60E-05 | 4.75E-05 |
| MIR25 | 2.066813 | 4.588749 | 1.150693 | 2.61E-22 | 3.78E-21 |
| IGHG4 | 14.58519 | 40.40409 | 1.469997 | 0.002334 | 0.003397 |
| CD79A | 3.583328 | 7.351025 | 1.036645 | 0.000549 | 0.000862 |
| MAMDC4 | 2.037574 | 4.286587 | 1.072977 | 7.08E-20 | 6.87E-19 |
| ORAOV1P1 | 1.709258 | 3.889573 | 1.186242 | 5.90E-19 | 5.02E-18 |
| G0S2 | 23.72267 | 48.57225 | 1.033866 | 7.42E-07 | 1.59E-06 |
| AL670729.3 | 0.843865 | 1.696662 | 1.007615 | 4.18E-15 | 2.20E-14 |
| ITIH2 | 0.210954 | 2.101425 | 3.316368 | 6.73E-11 | 2.22E-10 |
| CSAD | 2.530775 | 5.710425 | 1.174019 | 5.62E-25 | 1.33E-23 |
| IL20RB | 5.98354 | 14.98802 | 1.324739 | 8.12E-12 | 2.94E-11 |
| STAG3L5P | 1.297402 | 2.598153 | 1.001861 | 8.86E-16 | 5.08E-15 |
| AL353622.1 | 0.919253 | 1.992357 | 1.115943 | 2.01E-16 | 1.25E-15 |
| CRYBB3 | 1.00673 | 2.408801 | 1.258638 | 1.13E-09 | 3.30E-09 |
| LCAT | 3.291712 | 6.652429 | 1.015043 | 5.59E-24 | 1.11E-22 |
| SAA1 | 37.38494 | 179.2229 | 2.261225 | 1.67E-08 | 4.30E-08 |
| PI3 | 1.64512 | 7.839289 | 2.25253 | 7.93E-12 | 2.87E-11 |
| PTPRN | 0.557077 | 1.81308 | 1.702493 | 0.000297 | 0.000482 |
| TPSG1 | 1.489381 | 4.384347 | 1.557649 | 3.93E-05 | 7.03E-05 |
| TBC1D10C | 1.678881 | 3.402304 | 1.019012 | 1.04E-17 | 7.65E-17 |
| NFKBIZ | 3.019356 | 6.31391 | 1.064293 | 2.18E-14 | 1.06E-13 |
| ITGB2-AS1 | 0.892114 | 2.533914 | 1.506067 | 1.73E-23 | 3.12E-22 |
| YJEFN3 | 0.552036 | 1.534793 | 1.475211 | 4.64E-28 | 2.16E-26 |
| CDK5RAP3 | 0.584384 | 1.439672 | 1.300751 | 1.38E-26 | 4.53E-25 |
| MHENCR | 3.048229 | 7.689689 | 1.334954 | 1.63E-40 | 4.25E-37 |
| AC245884.8 | 0.732278 | 2.053492 | 1.487615 | 8.07E-34 | 1.78E-31 |
| VAMP1 | 1.543575 | 3.439242 | 1.155815 | 3.48E-25 | 8.50E-24 |
| TTLL3 | 0.859571 | 2.022294 | 1.234305 | 2.61E-26 | 8.11E-25 |
| MIR647 | 3.831916 | 8.823844 | 1.203341 | 6.97E-22 | 9.40E-21 |
| HSH2D | 0.725562 | 1.854138 | 1.353577 | 1.20E-22 | 1.86E-21 |
| TNNT1 | 0.401031 | 2.37894 | 2.568533 | 4.23E-13 | 1.77E-12 |
| AC024060.2 | 1.665775 | 3.587867 | 1.106933 | 3.59E-38 | 3.34E-35 |
| MICALL2 | 2.28016 | 4.946232 | 1.117195 | 1.87E-29 | 1.20E-27 |
| SLC38A5 | 2.044828 | 6.163088 | 1.591674 | 7.93E-10 | 2.36E-09 |
| ST8SIA6 | 3.020641 | 1.498455 | -1.01138 | 5.54E-05 | 9.74E-05 |
| SPINK13 | 2.725429 | 6.78899 | 1.316714 | 4.82E-05 | 8.54E-05 |
| SPINK1 | 3.458774 | 10.12198 | 1.54916 | 5.70E-10 | 1.72E-09 |
| AL139349.1 | 0.916578 | 2.653059 | 1.533327 | 4.91E-25 | 1.17E-23 |
| SPACA6 | 0.769695 | 1.728082 | 1.166814 | 2.29E-30 | 1.79E-28 |
| AC135050.3 | 0.86599 | 2.3459 | 1.437719 | 6.65E-31 | 5.94E-29 |
| RAC3 | 1.319845 | 2.643292 | 1.001967 | 3.77E-13 | 1.58E-12 |
| TAF5LP1 | 1.067497 | 2.280571 | 1.095162 | 2.04E-15 | 1.12E-14 |
| GALNT17 | 0.994978 | 2.507301 | 1.333399 | 0.015849 | 0.020865 |
| FAM24B | 1.177059 | 2.803439 | 1.252011 | 5.46E-26 | 1.59E-24 |
| HAGHL | 0.751251 | 1.900974 | 1.339371 | 3.75E-17 | 2.60E-16 |
| AC104695.3 | 1.385446 | 5.356077 | 1.950826 | 3.23E-25 | 7.97E-24 |
| EGR3 | 3.79216 | 1.819871 | -1.05918 | 3.07E-08 | 7.68E-08 |
| COL7A1 | 0.335959 | 1.781494 | 2.406729 | 4.71E-19 | 4.07E-18 |
| AC092535.5 | 3.003368 | 7.488713 | 1.318137 | 8.02E-16 | 4.64E-15 |
| MTATP8P1 | 2.741861 | 5.766719 | 1.072595 | 1.65E-06 | 3.42E-06 |
| SNORD104 | 4.366379 | 13.24713 | 1.601171 | 8.34E-30 | 5.72E-28 |
| ORM1 | 0.173961 | 8.794317 | 5.659736 | 0.001932 | 0.00284 |
| ACHE | 0.867924 | 2.130335 | 1.295439 | 7.48E-13 | 3.03E-12 |
| SCD5 | 13.54652 | 6.745798 | -1.00586 | 2.76E-09 | 7.74E-09 |
| AC048341.2 | 1.337754 | 4.036231 | 1.593195 | 1.92E-34 | 5.20E-32 |
| ZNF366 | 2.029667 | 1.011764 | -1.00437 | 1.60E-24 | 3.51E-23 |
| AC124854.1 | 7.908854 | 3.922725 | -1.01161 | 3.13E-14 | 1.50E-13 |
| PCK1 | 31.65058 | 15.38184 | -1.041 | 5.38E-10 | 1.62E-09 |
| NPIPB15 | 0.7474 | 2.095942 | 1.487647 | 8.35E-12 | 3.02E-11 |
| SNORD99 | 2.010809 | 8.904443 | 2.146749 | 8.21E-41 | 2.67E-37 |
| AC003092.1 | 0.708651 | 1.436319 | 1.01923 | 0.001823 | 0.002689 |
| VSTM2L | 0.584494 | 2.123142 | 1.860941 | 8.67E-08 | 2.06E-07 |
| SNORD100 | 1.135376 | 3.24186 | 1.513652 | 8.69E-27 | 2.95E-25 |
| FLRT3 | 10.35808 | 4.761518 | -1.12126 | 2.15E-19 | 1.94E-18 |
| RSRP1 | 4.811849 | 9.705466 | 1.012206 | 7.31E-25 | 1.69E-23 |
| SLC5A8 | 14.49843 | 6.78509 | -1.09546 | 1.94E-15 | 1.07E-14 |
| RUSC1-AS1 | 0.716873 | 1.436885 | 1.003156 | 1.56E-22 | 2.36E-21 |
| CD177 | 0.317502 | 2.341855 | 2.882816 | 0.003512 | 0.00501 |
| TRIM54 | 1.180361 | 3.172482 | 1.426383 | 0.005943 | 0.008235 |
| AL021707.8 | 0.998867 | 2.214884 | 1.148867 | 4.37E-22 | 6.08E-21 |
| ANKRD2 | 0.757384 | 1.590831 | 1.070684 | 0.035407 | 0.044529 |
| PCDHGA9 | 1.673303 | 0.783746 | -1.09424 | 1.83E-11 | 6.38E-11 |
| HSF4 | 14.39711 | 37.13349 | 1.366942 | 5.95E-19 | 5.05E-18 |
