# Supplementary material for: Construction and Validation of a Novel Cuproptosis-Related Seven-lncRNA Signature to Predict the Outcomes, Immunotherapeutic Responses, and Targeted Therapy in Patients with Clear Cell Renal Cell Carcinoma
Source: Dis Markers. 2023 Jan 25;2023:7219794. doi: 10.1155/2023/7219794 (PMC9893525; doi:10.1155/2023/7219794)
Supplement: Supplementary 11 — R codes used in this research. [file 7219794.f11.docx]

library(limma)

library(pheatmap)

library(reshape2)

library(ggpubr)

lncFile="timeexp.txt"

expFile="cuproptosisExp.txt"

setwd("C:\\Users\\Ohh\\Desktop\\cuprlnc2\\STEP1EXPRESSION")

rt=read.table(expFile, header=T, sep="\t", check.names=F)

rt=as.matrix(rt)

rownames(rt)=rt[,1]

exp=rt[,2:ncol(rt)]

dimnames=list(rownames(exp),colnames(exp))

data=matrix(as.numeric(as.matrix(exp)),nrow=nrow(exp),dimnames=dimnames)

data=avereps(data)

data=data[rowMeans(data)>0,]

lncRNA=read.table(lncFile, header=T, sep="\t", check.names=F, row.names=1)

data=data[colnames(lncRNA)[3:ncol(lncRNA)],]

exp=data

group=sapply(strsplit(colnames(data),"\\-"), "[", 4)

group=sapply(strsplit(group,""), "[", 1)

group=gsub("2", "1", group)

conNum=length(group[group==1])

treatNum=length(group[group==0])

sampleType=c(rep(1,conNum), rep(2,treatNum))

sigVec=c()

for(i in row.names(data)){

test=wilcox.test(data[i,] ~ sampleType)

pvalue=test$p.value

Sig=ifelse(pvalue<0.001,"***",ifelse(pvalue<0.01,"**",ifelse(pvalue<0.05,"*","")))

sigVec=c(sigVec, paste0(i, Sig))

}

row.names(data)=sigVec

exp=as.data.frame(t(exp))

exp=cbind(exp, Type=sampleType)

exp$Type=ifelse(exp$Type==1, "Normal", "Tumor")

data=melt(exp, id.vars=c("Type"))

colnames(data)=c("Type", "Gene", "Expression")

p=ggboxplot(data, x="Gene", y="Expression", fill = "Type",

ylab="Gene expression",

xlab="",

legend.title="Type",

palette = c("#608595", "#dfc223"),

width=1)

p=p+rotate_x_text(60)

p1=p+stat_compare_means(aes(group=Type),

method="wilcox.test",

symnum.args=list(cutpoints = c(0, 0.001, 0.01, 0.05, 1), symbols = c("***", "**", "*", " ")),

label = "p.signif")

pdf(file="boxplotgai.pdf", width=15, height=5)

print(p1)

dev.off()

options(stringsAsFactors=F)

library(limma)

library(ggpubr)

library(reshape2)

riskFile="tcgaRisk.txt"

scoreFile="ssgseaOut.txt"

setwd("D:\\biowolf\\Ferroptosis\\31.scoreCor\\TCGA")

data=read.table(scoreFile,sep="\t",header=T,check.names=F,row.names=1)

group=sapply(strsplit(colnames(data),"\\-"),"[",4)

group=sapply(strsplit(group,""),"[",1)

group=gsub("2","1",group)

data=data[,group==0]

colnames(data)=gsub("(.*?)\\-(.*?)\\-(.*?)\\-(.*?)\\-.*","\\1\\-\\2\\-\\3",colnames(data))

data=avereps(t(data))

risk=read.table(riskFile,header=T,sep="\t",row.names=1,check.names=F)

sameSample=intersect(row.names(data),row.names(risk))

data=data[sameSample,]

risk=risk[sameSample,]

rt=cbind(data,risk[,c("riskScore","risk")])

rt=rt[,-(ncol(rt)-1)]

immCell=c("aDCs","B_cells","CD8+_T_cells","DCs","iDCs","Macrophages",

"Mast_cells","Neutrophils","NK_cells","pDCs","T_helper_cells",

"Tfh","Th1_cells","Th2_cells","TIL","Treg")

rt1=rt[,c(immCell,"risk")]

data=melt(rt1,id.vars=c("risk"))

colnames(data)=c("Risk","Type","Score")

data$Risk=factor(data$Risk, levels=c("low","high"))

p=ggboxplot(data, x="Type", y="Score", color = "Risk",

ylab="Score",add = "none",xlab="",palette = c("blue","red") )

p=p+rotate_x_text(50)

pdf(file="immCell.boxplot.pdf",width=7,height=6)

p+stat_compare_means(aes(group=Risk),symnum.args=list(cutpoints = c(0, 0.001, 0.01, 0.05, 1), symbols = c("***", "**", "*", "ns")),label = "p.signif")

dev.off()

immFunction=c("APC_co_inhibition","APC_co_stimulation","CCR",

"Check-point","Cytolytic_activity","HLA","Inflammation-promoting",

"MHC_class_I","Parainflammation","T_cell_co-inhibition",

"T_cell_co-stimulation","Type_I_IFN_Reponse","Type_II_IFN_Reponse")

rt1=rt[,c(immFunction,"risk")]

data=melt(rt1,id.vars=c("risk"))

colnames(data)=c("Risk","Type","Score")

data$Risk=factor(data$Risk, levels=c("low","high"))

p=ggboxplot(data, x="Type", y="Score", color = "Risk",

ylab="Score",add = "none",xlab="",palette = c("blue","red") )

p=p+rotate_x_text(50)

pdf(file="immFunction.boxplot.pdf",width=7,height=6)

p+stat_compare_means(aes(group=Risk),symnum.args=list(cutpoints = c(0, 0.001, 0.01, 0.05, 1), symbols = c("***", "**", "*", "ns")),label = "p.signif")

dev.off()

library(survival)

library(survminer)

setwd("C:\\Users\\Ohh\\Desktop\\cuprlnc2\\STEP9Groupgai")

risk=read.table("risk.all.txt",header=T,sep="\t",check.names=F,row.names=1)

cli=read.table("clinical.txt",sep="\t",check.names=F,header=T,row.names=1)

sameSample=intersect(row.names(cli),row.names(risk))

risk=risk[sameSample,]

cli=cli[sameSample,]

data=cbind(futime=risk[,1],fustat=risk[,2],cli,risk=risk[,"risk"])

for(i in colnames(data[,3:(ncol(data)-1)])){

rt=data[,c("futime","fustat",i,"risk")]

rt=rt[(rt[,i]!="unknow"),]

colnames(rt)=c("futime","fustat","clinical","risk")

tab=table(rt[,"clinical"])

tab=tab[tab!=0]

for(j in names(tab)){

rt1=rt[(rt[,"clinical"]==j),]

tab1=table(rt1[,"risk"])

tab1=tab1[tab1!=0]

labels=paste0(names(tab1)," risk(n=",tab1,")")

if(length(labels)==2){

titleName=j

if((i=="age") | (i=="Age") | (i=="AGE")){

titleName=paste0("age",j)

}

diff=survdiff(Surv(futime, fustat) ~risk,data = rt1)

pValue=1-pchisq(diff$chisq,df=1)

pValue=signif(pValue,4)

pValue=format(pValue, scientific = TRUE)

fit <- survfit(Surv(futime, fustat) ~ risk, data = rt1)

surPlot=ggsurvplot(fit,

data=rt1,

surv.median.line = "hv",

conf.int=TRUE,

pval=paste0("p=",pValue),

pval.size=6,

legend.labs=labels,

legend.title=titleName,

font.legend=13,

xlab="Time(years)",

break.time.by = 1,

palette=c("#c07a92","#80afbf") )

j=gsub(">=","ge",j);j=gsub("<=","le",j);j=gsub(">","gt",j);j=gsub("<","lt",j)

pdf(file=paste0("survival.",i,"_",j,".pdf"),onefile = FALSE,

width = 5,

height =4.5)

print(surPlot)

dev.off()

}

}

}

library(limma)

corFilter=0.4

pvalueFilter=0.001

setwd("C:\\Users\\Ohh\\Desktop\\cuprlnc\\11")

rt=read.table("lncRNA.txt", header=T, sep="\t", check.names=F)

rt=as.matrix(rt)

rownames(rt)=rt[,1]

exp=rt[,2:ncol(rt)]

dimnames=list(rownames(exp),colnames(exp))

data=matrix(as.numeric(as.matrix(exp)),nrow=nrow(exp),dimnames=dimnames)

data=avereps(data)

data=data[rowMeans(data)>0.1,]

group=sapply(strsplit(colnames(data),"\\-"),"[",4)

group=sapply(strsplit(group,""), "[", 1)

group=gsub("2","1",group)

lncRNA=data[,group==0]

conNum=length(group[group==1])

treatNum=length(group[group==0])

sampleType=c(rep(1,conNum), rep(2,treatNum))

rt1=read.table("cuproptosisExp.txt", header=T, sep="\t", check.names=F)

rt1=as.matrix(rt1)

rownames(rt1)=rt1[,1]

exp1=rt1[,2:ncol(rt1)]

dimnames1=list(rownames(exp1),colnames(exp1))

cuproptosis=matrix(as.numeric(as.matrix(exp1)), nrow=nrow(exp1), dimnames=dimnames1)

cuproptosis=avereps(cuproptosis)

cuproptosis=cuproptosis[rowMeans(cuproptosis)>0.1,]

group=sapply(strsplit(colnames(cuproptosis),"\\-"),"[",4)

group=sapply(strsplit(group,""),"[",1)

group=gsub("2","1",group)

cuproptosis=cuproptosis[,group==0]

outTab=data.frame()

for(i in row.names(lncRNA)){

if(sd(lncRNA[i,])>0.1){

test=wilcox.test(data[i,] ~ sampleType)

if(test$p.value<0.05){

for(j in row.names(cuproptosis)){

x=as.numeric(lncRNA[i,])

y=as.numeric(cuproptosis[j,])

corT=cor.test(x,y)

cor=corT$estimate

pvalue=corT$p.value

if((cor>corFilter) & (pvalue<pvalueFilter)){

outTab=rbind(outTab,cbind(Cuproptosis=j,lncRNA=i,cor,pvalue,Regulation="postive"))

}

if((cor< -corFilter) & (pvalue<pvalueFilter)){

outTab=rbind(outTab,cbind(Cuproptosis=j,lncRNA=i,cor,pvalue,Regulation="negative"))

}

}

}

}

}

write.table(file="corResult.txt",outTab,sep="\t",quote=F,row.names=F)

cuproptosisLncRNA=unique(as.vector(outTab[,"lncRNA"]))

cuproptosisLncRNAexp=data[cuproptosisLncRNA,]

cuproptosisLncRNAexp=rbind(ID=colnames(cuproptosisLncRNAexp), cuproptosisLncRNAexp)

write.table(cuproptosisLncRNAexp,file="cuproptosisLncExp.txt",sep="\t",quote=F,col.names=F)

library(survival)

library(caret)

library(glmnet)

library(survminer)

library(timeROC)

coxPfilter=0.05

setwd("C:\\biowolf\\cuproptosis\\14.model")

rt=read.table("expTime.txt", header=T, sep="\t", check.names=F, row.names=1)

rt$futime[rt$futime<=0]=1

rt$futime=rt$futime/365

rt[,3:ncol(rt)]=log2(rt[,3:ncol(rt)]+1)

bioForest=function(coxFile=null,forestFile=null,forestCol=null){

rt <- read.table(coxFile,header=T,sep="\t",row.names=1,check.names=F)

gene <- rownames(rt)

hr <- sprintf("%.3f",rt$"HR")

hrLow <- sprintf("%.3f",rt$"HR.95L")

hrHigh <- sprintf("%.3f",rt$"HR.95H")

Hazard.ratio <- paste0(hr,"(",hrLow,"-",hrHigh,")")

pVal <- ifelse(rt$pvalue<0.001, "<0.001", sprintf("%.3f", rt$pvalue))

pdf(file=forestFile, width=7, height=6)

n <- nrow(rt)

nRow <- n+1

ylim <- c(1,nRow)

layout(matrix(c(1,2),nc=2),width=c(3,2.5))

xlim = c(0,3)

par(mar=c(4,2.5,2,1))

plot(1,xlim=xlim,ylim=ylim,type="n",axes=F,xlab="",ylab="")

text.cex=0.8

text(0,n:1,gene,adj=0,cex=text.cex)

text(1.5-0.5*0.2,n:1,pVal,adj=1,cex=text.cex);text(1.5-0.5*0.2,n+1,'pvalue',cex=text.cex,adj=1)

text(3,n:1,Hazard.ratio,adj=1,cex=text.cex);text(3,n+1,'Hazard ratio',cex=text.cex,adj=1,)

par(mar=c(4,1,2,1),mgp=c(2,0.5,0))

LOGindex = 10

hrLow = log(as.numeric(hrLow),LOGindex)

hrHigh = log(as.numeric(hrHigh),LOGindex)

hr = log(as.numeric(hr),LOGindex)

xlim = c(floor(min(hrLow,hrHigh)),ceiling(max(hrLow,hrHigh)))

plot(1,xlim=xlim,ylim=ylim,type="n",axes=F,ylab="",xaxs="i",xlab="Hazard ratio")

arrows(as.numeric(hrLow),n:1,as.numeric(hrHigh),n:1,angle=90,code=3,length=0.05,col="darkblue",lwd=2.5)

abline(v=log(1,LOGindex),col="black",lty=2,lwd=2)

boxcolor = ifelse(as.numeric(hr) > log(1,LOGindex), forestCol[1],forestCol[2])

points(as.numeric(hr), n:1, pch = 15, col = boxcolor, cex=1.3)

a1 = axis(1,labels=F,tick=F)

axis(1,a1,10^a1)

dev.off()

}

n=1

for(i in 1:n){

inTrain<-createDataPartition(y=rt[,2], p=0.5, list=F)

train<-rt[inTrain,]

test<-rt[-inTrain,]

trainOut=cbind(id=row.names(train),train)

testOut=cbind(id=row.names(test),test)

outUniTab=data.frame()

sigGenes=c("futime","fustat")

for(i in colnames(train[,3:ncol(train)])){

cox <- coxph(Surv(futime, fustat) ~ train[,i], data = train)

coxSummary = summary(cox)

coxP=coxSummary$coefficients[,"Pr(>|z|)"]

if(coxP<coxPfilter){

sigGenes=c(sigGenes,i)

outUniTab=rbind(outUniTab,

cbind(id=i,

HR=coxSummary$conf.int[,"exp(coef)"],

HR.95L=coxSummary$conf.int[,"lower .95"],

HR.95H=coxSummary$conf.int[,"upper .95"],

pvalue=coxSummary$coefficients[,"Pr(>|z|)"])

)

}

}

uniSigExp=train[,sigGenes]

uniSigExpOut=cbind(id=row.names(uniSigExp),uniSigExp)

if(ncol(uniSigExp)<6){next}

x=as.matrix(uniSigExp[,c(3:ncol(uniSigExp))])

y=data.matrix(Surv(uniSigExp$futime,uniSigExp$fustat))

fit <- glmnet(x, y, family = "cox", maxit = 1000)

cvfit <- cv.glmnet(x, y, family="cox", maxit = 1000)

coef <- coef(fit, s = cvfit$lambda.min)

index <- which(coef != 0)

actCoef <- coef[index]

lassoGene=row.names(coef)[index]

lassoSigExp=uniSigExp[,c("futime", "fustat", lassoGene)]

lassoSigExpOut=cbind(id=row.names(lassoSigExp), lassoSigExp)

geneCoef=cbind(Gene=lassoGene, Coef=actCoef)

if(nrow(geneCoef)<2){next}

multiCox <- coxph(Surv(futime, fustat) ~ ., data = lassoSigExp)

multiCox=step(multiCox, direction = "both")

multiCoxSum=summary(multiCox)

outMultiTab=data.frame()

outMultiTab=cbind(

coef=multiCoxSum$coefficients[,"coef"],

HR=multiCoxSum$conf.int[,"exp(coef)"],

HR.95L=multiCoxSum$conf.int[,"lower .95"],

HR.95H=multiCoxSum$conf.int[,"upper .95"],

pvalue=multiCoxSum$coefficients[,"Pr(>|z|)"])

outMultiTab=cbind(id=row.names(outMultiTab),outMultiTab)

outMultiTab=outMultiTab[,1:2]

riskScore=predict(multiCox,type="risk",newdata=train)

coxGene=rownames(multiCoxSum$coefficients)

coxGene=gsub("`","",coxGene)

outCol=c("futime","fustat",coxGene)

medianTrainRisk=median(riskScore)

risk=as.vector(ifelse(riskScore>medianTrainRisk,"high","low"))

trainRiskOut=cbind(id=rownames(cbind(train[,outCol],riskScore,risk)),cbind(train[,outCol],riskScore,risk))

riskScoreTest=predict(multiCox,type="risk",newdata=test)

riskTest=as.vector(ifelse(riskScoreTest>medianTrainRisk,"high","low"))

testRiskOut=cbind(id=rownames(cbind(test[,outCol],riskScoreTest,riskTest)),cbind(test[,outCol],riskScore=riskScoreTest,risk=riskTest))

diff=survdiff(Surv(futime, fustat) ~risk,data = train)

pValue=1-pchisq(diff$chisq, df=1)

diffTest=survdiff(Surv(futime, fustat) ~riskTest,data = test)

pValueTest=1-pchisq(diffTest$chisq, df=1)

predictTime=1

roc=timeROC(T=train$futime, delta=train$fustat,

marker=riskScore, cause=1,

times=c(predictTime), ROC=TRUE)

rocTest=timeROC(T=test$futime, delta=test$fustat,

marker=riskScoreTest, cause=1,

times=c(predictTime), ROC=TRUE)

if((pValue<0.01) & (roc$AUC[2]>0.68) & (pValueTest<0.02) & (rocTest$AUC[2]>0.65)){

write.table(trainOut,file="data.train.txt",sep="\t",quote=F,row.names=F)

write.table(testOut,file="data.test.txt",sep="\t",quote=F,row.names=F)

write.table(outUniTab,file="uni.trainCox.txt",sep="\t",row.names=F,quote=F)

write.table(uniSigExpOut,file="uni.SigExp.txt",sep="\t",row.names=F,quote=F)

bioForest(coxFile="uni.trainCox.txt",forestFile="uni.foreast.pdf",forestCol=c("red","green"))

write.table(lassoSigExpOut,file="lasso.SigExp.txt",sep="\t",row.names=F,quote=F)

pdf("lasso.lambda.pdf")

plot(fit, xvar = "lambda", label = TRUE)

dev.off()

pdf("lasso.cvfit.pdf")

plot(cvfit)

abline(v=log(c(cvfit$lambda.min,cvfit$lambda.1se)), lty="dashed")

dev.off()

write.table(outMultiTab,file="multiCox.txt",sep="\t",row.names=F,quote=F)

write.table(trainRiskOut,file="risk.train.txt",sep="\t",quote=F,row.names=F)

write.table(testRiskOut,file="risk.test.txt",sep="\t",quote=F,row.names=F)

allRiskOut=rbind(trainRiskOut, testRiskOut)

write.table(allRiskOut,file="risk.all.txt",sep="\t",quote=F,row.names=F)

break

}

}

library(limma)

expFile="symbol.txt"

riskFile="risk.all.txt"

logFCfilter=1

fdrFilter=0.05

setwd("C:\\Users\\Ohh\\Desktop\\cuprlnc2\\STEP11GOKEGG")

rt=read.table(expFile, header=T, sep="\t", check.names=F)

rt=as.matrix(rt)

rownames(rt)=rt[,1]

exp=rt[,2:ncol(rt)]

dimnames=list(rownames(exp), colnames(exp))

data=matrix(as.numeric(as.matrix(exp)), nrow=nrow(exp), dimnames=dimnames)

data=avereps(data)

group=sapply(strsplit(colnames(data),"\\-"), "[", 4)

group=sapply(strsplit(group,""), "[", 1)

group=gsub("2", "1", group)

data=data[,group==0]

data=t(data)

rownames(data)=gsub("(.*?)\\-(.*?)\\-(.*?)\\-(.*?)\\-.*", "\\1\\-\\2\\-\\3", rownames(data))

data=avereps(data)

data=t(data)

risk=read.table(riskFile, header=T, sep="\t", check.names=F, row.names=1)

sameSample=intersect(colnames(data), row.names(risk))

data=data[,sameSample]

risk=risk[sameSample,]

riskLow=risk[risk$risk=="low",]

riskHigh=risk[risk$risk=="high",]

dataLow=data[,row.names(riskLow)]

dataHigh=data[,row.names(riskHigh)]

data=cbind(dataLow,dataHigh)

data=data[rowMeans(data)>1,]

conNum=ncol(dataLow)

treatNum=ncol(dataHigh)

Type=c(rep(1,conNum), rep(2,treatNum))

outTab=data.frame()

for(i in row.names(data)){

rt=data.frame(expression=data[i,], Type=Type)

wilcoxTest=wilcox.test(expression ~ Type, data=rt)

pvalue=wilcoxTest$p.value

conGeneMeans=mean(data[i,1:conNum])

treatGeneMeans=mean(data[i,(conNum+1):ncol(data)])

logFC=log2(treatGeneMeans)-log2(conGeneMeans)

conMed=median(data[i,1:conNum])

treatMed=median(data[i,(conNum+1):ncol(data)])

diffMed=treatMed-conMed

if( ((logFC>0) & (diffMed>0)) | ((logFC<0) & (diffMed<0)) ){

outTab=rbind(outTab,cbind(gene=i,lowMean=conGeneMeans,highMean=treatGeneMeans,logFC=logFC,pValue=pvalue))

}

}

pValue=outTab[,"pValue"]

fdr=p.adjust(as.numeric(as.vector(pValue)), method="fdr")

outTab=cbind(outTab, fdr=fdr)

outDiff=outTab[( abs(as.numeric(as.vector(outTab$logFC)))>logFCfilter & as.numeric(as.vector(outTab$fdr))<fdrFilter),]

write.table(outDiff, file="riskDiff.txt", sep="\t", row.names=F, quote=F)

library(limma)

library(ggpubr)

library(pRRophetic)

library(ggplot2)

set.seed(12345)

pFilter=0.001

expFile="symbol.txt"

riskFile="risk.all.txt"

setwd("C:\\Users\\Ohh\\Desktop\\cuprlnc\\STEP14pRRophetic")

data(cgp2016ExprRma)

data(PANCANCER_IC_Tue_Aug_9_15_28_57_2016)

allDrugs=unique(drugData2016$Drug.name)

rt = read.table(expFile, header=T, sep="\t", check.names=F)

rt=as.matrix(rt)

rownames(rt)=rt[,1]

exp=rt[,2:ncol(rt)]

dimnames=list(rownames(exp),colnames(exp))

data=matrix(as.numeric(as.matrix(exp)),nrow=nrow(exp),dimnames=dimnames)

data=avereps(data)

data=data[rowMeans(data)>0.5,]

group=sapply(strsplit(colnames(data),"\\-"), "[", 4)

group=sapply(strsplit(group,""), "[", 1)

group=gsub("2","1",group)

data=data[,group==0]

data=t(data)

rownames(data)=gsub("(.*?)\\-(.*?)\\-(.*?)\\-(.*)", "\\1\\-\\2\\-\\3", rownames(data))

data=avereps(data)

data=t(data)

riskRT=read.table(riskFile, header=T, sep="\t", check.names=F, row.names=1)

riskRT$riskScore[riskRT$riskScore>quantile(riskRT$riskScore,0.99)]=quantile(riskRT$riskScore,0.99)

for(drug in allDrugs){

possibleError=tryCatch(

{senstivity=pRRopheticPredict(data, drug, selection=1, dataset = "cgp2016")},

error=function(e) e)

if(inherits(possibleError, "error")){next}

senstivity=senstivity[senstivity!="NaN"]

senstivity[senstivity>quantile(senstivity,0.99)]=quantile(senstivity,0.99)

sameSample=intersect(row.names(riskRT), names(senstivity))

risk=riskRT[sameSample, c("riskScore","risk"),drop=F]

senstivity=senstivity[sameSample]

rt=cbind(risk, senstivity)

rt$risk=factor(rt$risk, levels=c("low", "high"))

type=levels(factor(rt[,"risk"]))

comp=combn(type, 2)

my_comparisons=list()

for(i in 1:ncol(comp)){my_comparisons[[i]]<-comp[,i]}

test=wilcox.test(senstivity~risk, data=rt)

diffPvalue=test$p.value

x=as.numeric(rt[,"riskScore"])

y=as.numeric(rt[,"senstivity"])

corT=cor.test(x, y, method="spearman")

corPvalue=corT$p.value

if((diffPvalue<pFilter) & (corPvalue<pFilter)){

boxplot=ggboxplot(rt, x="risk", y="senstivity", fill="risk",

xlab="Risk",

ylab=paste0(drug, " senstivity (IC50)"),

legend.title="Risk",

palette=c("#00b0ff","#ff9100")

)+

stat_compare_means(comparisons=my_comparisons)

pdf(file=paste0("durgSenstivity.", drug, ".pdf"), width=5, height=4.5)

print(boxplot)

dev.off()

df1=as.data.frame(cbind(x,y))

p1=ggplot(df1, aes(x, y)) +

xlab("Risk score") + ylab(paste0(drug, " senstivity (IC50)"))+

geom_point(colour = "#ff9100", size = 1) + geom_smooth(method="lm",color="#212121",formula = y ~ x) + theme_grey()+

stat_cor(method = 'spearman', aes(x =x, y =y))

pdf(file=paste0("Cor.", drug, ".pdf"), width=5, height=4.6)

print(p1)

dev.off()

}

}

library(survival)

library(survminer)

inputFile="expTime.txt"

gene="SMARCA5-AS1"

setwd("C:\\Users\\Ohh\\Desktop\\cuprlnc")

rt=read.table(inputFile,header=T,sep="\t",check.names=F)

rt$futime=rt$futime/365

a=ifelse(rt[,gene]<=median(rt[,gene]),"Low","High")

diff=survdiff(Surv(futime, fustat) ~a,data = rt)

pValue=1-pchisq(diff$chisq,df=1)

fit=survfit(Surv(futime, fustat) ~ a, data = rt)

pValue=signif(pValue,4)

pValue=format(pValue, scientific = TRUE)

titleName=gene

surPlot=ggsurvplot(fit,

data=rt,

surv.median.line = "hv",

conf.int=TRUE,

pval=paste0("p=",pValue),

pval.size=6,

#ncensor.plot = TRUE,

legend.labs=c("high","low"),

legend.title=titleName,

xlab="Time(years)",

break.time.by = 1,

risk.table.title="",

palette=c("#c07a92", "#80afbf"),

risk.table.height=.25)

pdf(file=paste0("sur.",gene,".pdf"), width = 6.5, height = 5.5,onefile = FALSE)

print(surPlot)

dev.off()

library(clusterProfiler)

library(org.Hs.eg.db)

library(enrichplot)

library(ggplot2)

library(circlize)

library(RColorBrewer)

library(dplyr)

library("ggpubr")

library(ComplexHeatmap)

pvalueFilter=0.05

qvalueFilter=0.05

colorSel="qvalue"

if(qvalueFilter>0.05){

colorSel="pvalue"

}

setwd("C:\\Users\\Ohh\\Desktop\\cuprlnc2\\STEP11GOKEGG")

rt=read.table("riskDiff.txt", header=T, sep="\t", check.names=F)

genes=unique(as.vector(rt[,1]))

entrezIDs=mget(genes, org.Hs.egSYMBOL2EG, ifnotfound=NA)

entrezIDs=as.character(entrezIDs)

gene=entrezIDs[entrezIDs!="NA"]

#gene=gsub("c\\(\"(\\d+)\".*", "\\1", gene)

kk=enrichGO(gene=gene, OrgDb=org.Hs.eg.db, pvalueCutoff=1, qvalueCutoff=1, ont="all", readable=T)

GO=as.data.frame(kk)

GO=GO[(GO$pvalue<pvalueFilter & GO$qvalue<qvalueFilter),]

write.table(GO, file="GO.txt", sep="\t", quote=F, row.names = F)

showNum=10

if(nrow(GO)<30){

showNum=nrow(GO)

}

pdf(file="barplot.pdf", width=8, height=7)

bar=barplot(kk, drop=TRUE, showCategory=showNum, label_format=30, split="ONTOLOGY", color=colorSel) + facet_grid(ONTOLOGY~., scale='free')

print(bar)

dev.off()

pdf(file="bubble.pdf", width=8, height=7)

bub=dotplot(kk, showCategory=showNum, orderBy="GeneRatio", label_format=30, split="ONTOLOGY", color=colorSel) + facet_grid(ONTOLOGY~., scale='free')

print(bub)

dev.off()

data=GO %>% group_by(ONTOLOGY) %>% slice_head(n=10)

pdf(file="barplot.color.pdf", width=8, height=6.5)

ggbarplot(data, x="Description", y="Count", fill = "ONTOLOGY", color = "white",

xlab="Term",

orientation = "horiz",

palette = "aaas",

legend = "right",

sort.val = "asc",

sort.by.groups=TRUE)+

scale_y_continuous(expand=c(0, 0)) + scale_x_discrete(expand=c(0,0))

dev.off()

ontology.col=c("#00AFBB", "#E7B800", "#90EE90")

data=GO[order(GO$p.adjust),]

datasig=data[data$p.adjust<0.05,,drop=F]

BP = datasig[datasig$ONTOLOGY=="BP",,drop=F]

CC = datasig[datasig$ONTOLOGY=="CC",,drop=F]

MF = datasig[datasig$ONTOLOGY=="MF",,drop=F]

BP = head(BP,6)

CC = head(CC,6)

MF = head(MF,6)

data = rbind(BP,CC,MF)

main.col = ontology.col[as.numeric(as.factor(data$ONTOLOGY))]

BgGene = as.numeric(sapply(strsplit(data$BgRatio,"/"),'[',1))

Gene = as.numeric(sapply(strsplit(data$GeneRatio,'/'),'[',1))

ratio = Gene/BgGene

logpvalue = -log(data$pvalue,10)

logpvalue.col = brewer.pal(n = 8, name = "Reds")

f = colorRamp2(breaks = c(0,2,4,6,8,10,15,20), colors = logpvalue.col)

BgGene.col = f(logpvalue)

df = data.frame(GO=data$ID,start=1,end=max(BgGene))

rownames(df) = df$GO

bed2 = data.frame(GO=data$ID,start=1,end=BgGene,BgGene=BgGene,BgGene.col=BgGene.col)

bed3 = data.frame(GO=data$ID,start=1,end=Gene,BgGene=Gene)

bed4 = data.frame(GO=data$ID,start=1,end=max(BgGene),ratio=ratio,col=main.col)

bed4$ratio = bed4$ratio/max(bed4$ratio)*9.5

pdf("GO.circlize.pdf",width=10,height=10)

par(omi=c(0.1,0.1,0.1,1.5))

circos.par(track.margin=c(0.01,0.01))

circos.genomicInitialize(df,plotType="none")

circos.trackPlotRegion(ylim = c(0, 1), panel.fun = function(x, y) {

sector.index = get.cell.meta.data("sector.index")

xlim = get.cell.meta.data("xlim")

ylim = get.cell.meta.data("ylim")

circos.text(mean(xlim), mean(ylim), sector.index, cex = 0.8, facing = "bending.inside", niceFacing = TRUE)

}, track.height = 0.08, bg.border = NA,bg.col = main.col)

for(si in get.all.sector.index()) {

circos.axis(h = "top", labels.cex = 0.6, sector.index = si,track.index = 1,

major.at=seq(0,max(BgGene),by=100),labels.facing = "clockwise")

}

f = colorRamp2(breaks = c(-1, 0, 1), colors = c("green", "black", "red"))

circos.genomicTrack(bed2, ylim = c(0, 1),track.height = 0.1,bg.border="white",

panel.fun = function(region, value, ...) {

i = getI(...)

circos.genomicRect(region, value, ytop = 0, ybottom = 1, col = value[,2],

border = NA, ...)

circos.genomicText(region, value, y = 0.4, labels = value[,1], adj=0,cex=0.8,...)

})

circos.genomicTrack(bed3, ylim = c(0, 1),track.height = 0.1,bg.border="white",

panel.fun = function(region, value, ...) {

i = getI(...)

circos.genomicRect(region, value, ytop = 0, ybottom = 1, col = '#BA55D3',

border = NA, ...)

circos.genomicText(region, value, y = 0.4, labels = value[,1], cex=0.9,adj=0,...)

})

circos.genomicTrack(bed4, ylim = c(0, 10),track.height = 0.35,bg.border="white",bg.col="grey90",

panel.fun = function(region, value, ...) {

cell.xlim = get.cell.meta.data("cell.xlim")

cell.ylim = get.cell.meta.data("cell.ylim")

for(j in 1:9) {

y = cell.ylim[1] + (cell.ylim[2]-cell.ylim[1])/10*j

circos.lines(cell.xlim, c(y, y), col = "#FFFFFF", lwd = 0.3)

}

circos.genomicRect(region, value, ytop = 0, ybottom = value[,1], col = value[,2],

border = NA, ...)

#circos.genomicText(region, value, y = 0.3, labels = value[,1], ...)

})

circos.clear()

middle.legend = Legend(

labels = c('Number of Genes','Number of Select','Rich Factor(0-1)'),

type="points",pch=c(15,15,17),legend_gp = gpar(col=c('pink','#BA55D3',ontology.col[1])),

title="",nrow=3,size= unit(3, "mm")

)

circle_size = unit(1, "snpc")

draw(middle.legend,x=circle_size*0.42)

main.legend = Legend(

labels = c("Biological Process","Cellular Component", "Molecular Function"), type="points",pch=15,

legend_gp = gpar(col=ontology.col), title_position = "topcenter",

title = "ONTOLOGY", nrow = 3,size = unit(3, "mm"),grid_height = unit(5, "mm"),

grid_width = unit(5, "mm")

)

logp.legend = Legend(

labels=c('(0,2]','(2,4]','(4,6]','(6,8]','(8,10]','(10,15]','(15,20]','>=20'),

type="points",pch=16,legend_gp=gpar(col=logpvalue.col),title="-log10(Pvalue)",

title_position = "topcenter",grid_height = unit(5, "mm"),grid_width = unit(5, "mm"),

size = unit(3, "mm")

)

lgd = packLegend(main.legend,logp.legend)

circle_size = unit(1, "snpc")

print(circle_size)

draw(lgd, x = circle_size*0.85, y=circle_size*0.55,just = "left")

dev.off()

library(survival)

setwd("C:\\Users\\Ohh\\Desktop\\cuprlnc\\STEP8Roc cox")

bioForest=function(coxFile=null, forestFile=null, forestCol=null){

rt <- read.table(coxFile, header=T, sep="\t", check.names=F, row.names=1)

gene <- rownames(rt)

hr <- sprintf("%.3f",rt$"HR")

hrLow <- sprintf("%.3f",rt$"HR.95L")

hrHigh <- sprintf("%.3f",rt$"HR.95H")

Hazard.ratio <- paste0(hr,"(",hrLow,"-",hrHigh,")")

pVal <- ifelse(rt$pvalue<0.001, "<0.001", sprintf("%.3f", rt$pvalue))

pdf(file=forestFile, width=6.6, height=4.5)

n <- nrow(rt)

nRow <- n+1

ylim <- c(1,nRow)

layout(matrix(c(1,2),nc=2),width=c(3,2.5))

xlim = c(0,3)

par(mar=c(4,2.5,2,1))

plot(1,xlim=xlim,ylim=ylim,type="n",axes=F,xlab="",ylab="")

text.cex=0.8

text(0,n:1,gene,adj=0,cex=text.cex)

text(1.5-0.5*0.2,n:1,pVal,adj=1,cex=text.cex);text(1.5-0.5*0.2,n+1,'pvalue',cex=text.cex,font=2,adj=1)

text(3.1,n:1,Hazard.ratio,adj=1,cex=text.cex);text(3.1,n+1,'Hazard ratio',cex=text.cex,font=2,adj=1)

par(mar=c(4,1,2,1),mgp=c(2,0.5,0))

xlim = c(0,max(as.numeric(hrLow),as.numeric(hrHigh)))

plot(1,xlim=xlim,ylim=ylim,type="n",axes=F,ylab="",xaxs="i",xlab="Hazard ratio")

arrows(as.numeric(hrLow),n:1,as.numeric(hrHigh),n:1,angle=90,code=3,length=0.05,col="darkblue",lwd=2.5)

abline(v=1,col="black",lty=2,lwd=2)

boxcolor = ifelse(as.numeric(hr) > 1, forestCol, forestCol)

points(as.numeric(hr), n:1, pch = 15, col = boxcolor, cex=1.5)

axis(1)

dev.off()

}

indep=function(riskFile=null, cliFile=null, project=null){

risk=read.table(riskFile, header=T, sep="\t", check.names=F, row.names=1)

cli=read.table(cliFile, header=T, sep="\t", check.names=F, row.names=1)

sameSample=intersect(row.names(cli),row.names(risk))

risk=risk[sameSample,]

cli=cli[sameSample,]

rt=cbind(futime=risk[,1], fustat=risk[,2], cli, riskScore=risk[,(ncol(risk)-1)])

uniCoxFile=paste0(project,".uniCox.txt")

uniCoxPdf=paste0(project,".uniCox.pdf")

uniTab=data.frame()

for(i in colnames(rt[,3:ncol(rt)])){

cox <- coxph(Surv(futime, fustat) ~ rt[,i], data = rt)

coxSummary = summary(cox)

uniTab=rbind(uniTab,

cbind(id=i,

HR=coxSummary$conf.int[,"exp(coef)"],

HR.95L=coxSummary$conf.int[,"lower .95"],

HR.95H=coxSummary$conf.int[,"upper .95"],

pvalue=coxSummary$coefficients[,"Pr(>|z|)"])

)

}

write.table(uniTab,file=uniCoxFile,sep="\t",row.names=F,quote=F)

bioForest(coxFile=uniCoxFile, forestFile=uniCoxPdf, forestCol="green")

multiCoxFile=paste0(project,".multiCox.txt")

multiCoxPdf=paste0(project,".multiCox.pdf")

uniTab=uniTab[as.numeric(uniTab[,"pvalue"])<1,]

rt1=rt[,c("futime","fustat",as.vector(uniTab[,"id"]))]

multiCox=coxph(Surv(futime, fustat) ~ ., data = rt1)

multiCoxSum=summary(multiCox)

multiTab=data.frame()

multiTab=cbind(

HR=multiCoxSum$conf.int[,"exp(coef)"],

HR.95L=multiCoxSum$conf.int[,"lower .95"],

HR.95H=multiCoxSum$conf.int[,"upper .95"],

pvalue=multiCoxSum$coefficients[,"Pr(>|z|)"])

multiTab=cbind(id=row.names(multiTab),multiTab)

write.table(multiTab, file=multiCoxFile, sep="\t", row.names=F, quote=F)

bioForest(coxFile=multiCoxFile, forestFile=multiCoxPdf, forestCol="red")

}

indep(riskFile="risk.train.txt", cliFile="clinical.txt", project="all")

library(clusterProfiler)

library(org.Hs.eg.db)

library(enrichplot)

library(ggplot2)

library(circlize)

library(RColorBrewer)

library(dplyr)

library(ComplexHeatmap)

pvalueFilter=0.05

qvalueFilter=0.05

colorSel="qvalue"

if(qvalueFilter>0.05){

colorSel="pvalue"

}

setwd("C:\\Users\\Ohh\\Desktop\\cuprlnc2\\STEP11GOKEGGgai")

rt=read.table("riskDiff.txt", header=T, sep="\t", check.names=F)

genes=unique(as.vector(rt[,1]))

entrezIDs=mget(genes, org.Hs.egSYMBOL2EG, ifnotfound=NA)

entrezIDs=as.character(entrezIDs)

rt=data.frame(genes, entrezID=entrezIDs)

gene=entrezIDs[entrezIDs!="NA"]

#gene=gsub("c\\(\"(\\d+)\".*", "\\1", gene)

kk <- enrichKEGG(gene=gene, organism="hsa", pvalueCutoff=1, qvalueCutoff=1)

KEGG=as.data.frame(kk)

KEGG$geneID=as.character(sapply(KEGG$geneID,function(x)paste(rt$genes[match(strsplit(x,"/")[[1]],as.character(rt$entrezID))],collapse="/")))

KEGG=KEGG[(KEGG$pvalue<pvalueFilter & KEGG$qvalue<qvalueFilter),]

write.table(KEGG, file="KEGG.txt", sep="\t", quote=F, row.names = F)

showNum=30

if(nrow(KEGG)<showNum){

showNum=nrow(KEGG)

}

pdf(file="barplot.pdf", width=9, height=7)

barplot(kk, drop=TRUE, showCategory=showNum, label_format=130, color=colorSel)

dev.off()

pdf(file="bubble.pdf", width = 9, height = 7)

dotplot(kk, showCategory=showNum, orderBy="GeneRatio", label_format=130, color=colorSel)

dev.off()

Pathway.col=c("#90EE90", "#E7B800", "#00AFBB")

showNum=18

data=KEGG[order(KEGG$p.adjust),]

if(nrow(KEGG)>showNum){

data=data[1:showNum,]

}

data$Pathway="KEGG"

main.col = Pathway.col[as.numeric(as.factor(data$Pathway))]

BgGene = as.numeric(sapply(strsplit(data$BgRatio,"/"),'[',1))

Gene = as.numeric(sapply(strsplit(data$GeneRatio,'/'),'[',1))

ratio = Gene/BgGene

logpvalue = -log(data$pvalue,10)

logpvalue.col = brewer.pal(n = 8, name = "Reds")

f = colorRamp2(breaks = c(0,2,4,6,8,10,15,20), colors = logpvalue.col)

BgGene.col = f(logpvalue)

df = data.frame(KEGG=data$ID,start=1,end=max(BgGene))

rownames(df) = df$KEGG

bed2 = data.frame(KEGG=data$ID,start=1,end=BgGene,BgGene=BgGene,BgGene.col=BgGene.col)

bed3 = data.frame(KEGG=data$ID,start=1,end=Gene,BgGene=Gene)

bed4 = data.frame(KEGG=data$ID,start=1,end=max(BgGene),ratio=ratio,col=main.col)

bed4$ratio = bed4$ratio/max(bed4$ratio)*9.5

pdf(file="KEGG.circlize.pdf",width=10,height=10)

par(omi=c(0.1,0.1,0.1,1.5))

circos.par(track.margin=c(0.01,0.01))

circos.genomicInitialize(df,plotType="none")

circos.trackPlotRegion(ylim = c(0, 1), panel.fun = function(x, y) {

sector.index = get.cell.meta.data("sector.index")

xlim = get.cell.meta.data("xlim")

ylim = get.cell.meta.data("ylim")

circos.text(mean(xlim), mean(ylim), sector.index, cex = 0.8, facing = "bending.inside", niceFacing = TRUE)

}, track.height = 0.08, bg.border = NA,bg.col = main.col)

for(si in get.all.sector.index()) {

circos.axis(h = "top", labels.cex = 0.6, sector.index = si,track.index = 1,

major.at=seq(0,max(BgGene),by=100),labels.facing = "clockwise")

}

f = colorRamp2(breaks = c(-1, 0, 1), colors = c("green", "black", "red"))

circos.genomicTrack(bed2, ylim = c(0, 1),track.height = 0.1,bg.border="white",

panel.fun = function(region, value, ...) {

i = getI(...)

circos.genomicRect(region, value, ytop = 0, ybottom = 1, col = value[,2],

border = NA, ...)

circos.genomicText(region, value, y = 0.4, labels = value[,1], adj=0,cex=0.8,...)

})

circos.genomicTrack(bed3, ylim = c(0, 1),track.height = 0.1,bg.border="white",

panel.fun = function(region, value, ...) {

i = getI(...)

circos.genomicRect(region, value, ytop = 0, ybottom = 1, col = '#BA55D3',

border = NA, ...)

circos.genomicText(region, value, y = 0.4, labels = value[,1], cex=0.9,adj=0,...)

})

circos.genomicTrack(bed4, ylim = c(0, 10),track.height = 0.35,bg.border="white",bg.col="grey90",

panel.fun = function(region, value, ...) {

cell.xlim = get.cell.meta.data("cell.xlim")

cell.ylim = get.cell.meta.data("cell.ylim")

for(j in 1:9) {

y = cell.ylim[1] + (cell.ylim[2]-cell.ylim[1])/10*j

circos.lines(cell.xlim, c(y, y), col = "#FFFFFF", lwd = 0.3)

}

circos.genomicRect(region, value, ytop = 0, ybottom = value[,1], col = value[,2],

border = NA, ...)

#circos.genomicText(region, value, y = 0.3, labels = value[,1], ...)

})

circos.clear()

middle.legend = Legend(

labels = c('Number of Genes','Number of Select','Rich Factor(0-1)'),

type="points",pch=c(15,15,17),legend_gp = gpar(col=c('pink','#BA55D3',Pathway.col[1])),

title="",nrow=3,size= unit(3, "mm")

)

circle_size = unit(1, "snpc")

draw(middle.legend,x=circle_size*0.42)

main.legend = Legend(

labels = c("KEGG"), type="points",pch=15,

legend_gp = gpar(col=Pathway.col), title_position = "topcenter",

title = "Pathway", nrow = 3,size = unit(3, "mm"),grid_height = unit(5, "mm"),

grid_width = unit(5, "mm")

)

logp.legend = Legend(

labels=c('(0,2]','(2,4]','(4,6]','(6,8]','(8,10]','(10,15]','(15,20]','>=20'),

type="points",pch=16,legend_gp=gpar(col=logpvalue.col),title="-log10(Pvalue)",

title_position = "topcenter",grid_height = unit(5, "mm"),grid_width = unit(5, "mm"),

size = unit(3, "mm")

)

lgd = packLegend(main.legend,logp.legend)

circle_size = unit(1, "snpc")

print(circle_size)

draw(lgd, x = circle_size*0.85, y=circle_size*0.55,just = "left")

dev.off()

library(glmnet)

library(survival)

setwd("C:\\Users\\Ohh\\Desktop\\cuprlnc2\\STEP1EXPRESSION")

tcga<-read.table("cuproptosisExp.txt",header = T,sep = "\t",check.names = F)

tcga=as.matrix(tcga)

rownames(tcga)=tcga[,1]

GeneExp=tcga[,2:ncol(tcga)]

TCGA=matrix(as.numeric(as.matrix(GeneExp)),nrow=nrow(GeneExp),dimnames=list(rownames(GeneExp),colnames(GeneExp)))

st<- which(substr(colnames(TCGA),14,15) == '01')

tumor=TCGA[,st]

tumor=as.data.frame(tumor)

nc=substr(colnames(tumor),1,12)

colnames(tumor)=nc

tumor=cbind(id=row.names(tcga),tumor)

#head(tumor)

tumor=t(tumor)

write.table(tumor,"tumor.txt",sep = "\t",quote = F,col.names = F)

mRNA=read.table("tumor.txt",header = T,sep = "\t",check.names =F)

clidata=read.table("PFStime.txt",header = T,sep = "\t",check.names =F)

cliexp=merge(clidata,mRNA,by="id")

write.table(cliexp,"PFStimeexp.txt",sep = "\t",quote = F,row.names = F)

library(survival)

library(survminer)

library(timeROC)

riskFile="risk.train.txt"

cliFile="clinical.txt"

setwd("C:\\Users\\Ohh\\Desktop\\cuprlnc2\\STEP8Roc cox")

risk=read.table(riskFile, header=T, sep="\t", check.names=F, row.names=1)

risk=risk[,c("futime", "fustat", "riskScore")]

cli=read.table(cliFile, header=T, sep="\t", check.names=F, row.names=1)

samSample=intersect(row.names(risk), row.names(cli))

risk1=risk[samSample,,drop=F]

cli=cli[samSample,,drop=F]

rt=cbind(risk1, cli)

bioCol=c("#6e9ece","black","#dfc286","#e6928f","#4e9595","#dfc286","black")

#if(ncol(rt)>6){

# bioCol=rainbow(ncol(rt))}

ROC_rt=timeROC(T=risk$futime,delta=risk$fustat,

marker=risk$riskScore,cause=1,

weighting='aalen',

times=c(1,3,5),ROC=TRUE)

pdf(file="ROC.pdf", width=5, height=5)

plot(ROC_rt,time=1,col=bioCol[1],title=FALSE,lwd=2.5)

plot(ROC_rt,time=3,col=bioCol[2],add=TRUE,title=FALSE,lwd=2.5)

plot(ROC_rt,time=5,col=bioCol[3],add=TRUE,title=FALSE,lwd=2.5)

legend('bottomright',

c(paste0('AUC at 1 years: ',sprintf("%.03f",ROC_rt$AUC[1])),

paste0('AUC at 3 years: ',sprintf("%.03f",ROC_rt$AUC[2])),

paste0('AUC at 5 years: ',sprintf("%.03f",ROC_rt$AUC[3]))),

col=bioCol[1:3], lwd=2.5, bty = 'n')

dev.off()

predictTime=5

aucText=c()

pdf(file="train.cliROC.pdf", width=5, height=5)

i=3

ROC_rt=timeROC(T=risk$futime,

delta=risk$fustat,

marker=risk$riskScore, cause=1,

weighting='aalen',

times=c(predictTime),ROC=TRUE)

plot(ROC_rt, time=predictTime, col=bioCol[i-2], title=FALSE, lwd=2.5)

aucText=c(paste0("Risk", ", AUC=", sprintf("%.3f",ROC_rt$AUC[2])))

abline(0,1)

for(i in 4:ncol(rt)){

ROC_rt=timeROC(T=rt$futime,

delta=rt$fustat,

marker=rt[,i], cause=1,

weighting='aalen',

times=c(predictTime),ROC=TRUE)

plot(ROC_rt, time=predictTime, col=bioCol[i-2], title=FALSE, lwd=2.5, add=TRUE)

aucText=c(aucText, paste0(colnames(rt)[i],", AUC=",sprintf("%.3f",ROC_rt$AUC[2])))

}

legend("bottomright", aucText,lwd=2.5,bty="n",col=bioCol[1:(ncol(rt)-1)])

dev.off()

library(survival)

library(regplot)

library(rms)

riskFile="risk.all.txt"

cliFile="clinical.txt"

setwd("C:\\biowolf\\cuproptosis\\23.Nomo")

risk=read.table(riskFile, header=T, sep="\t", check.names=F, row.names=1)

cli=read.table(cliFile, header=T, sep="\t", check.names=F, row.names=1)

cli=cli[apply(cli,1,function(x)any(is.na(match('unknow',x)))),,drop=F]

cli$Age=as.numeric(cli$Age)

samSample=intersect(row.names(risk), row.names(cli))

risk1=risk[samSample,,drop=F]

cli=cli[samSample,,drop=F]

rt=cbind(risk1[,c("futime", "fustat", "risk")], cli)

res.cox=coxph(Surv(futime, fustat) ~ . , data = rt)

nom1=regplot(res.cox,

plots = c("density", "boxes"),

clickable=F,

title="",

points=TRUE,

droplines=TRUE,

observation=rt[20,],

rank="sd",

failtime = c(1,3,5),

prfail = F)

nomoRisk=predict(res.cox, data=rt, type="risk")

rt=cbind(risk1, Nomogram=nomoRisk)

outTab=rbind(ID=colnames(rt), rt)

write.table(outTab, file="nomoRisk.txt", sep="\t", col.names=F, quote=F)

pdf(file="calibration.pdf", width=5, height=5)

f <- cph(Surv(futime, fustat) ~ Nomogram, x=T, y=T, surv=T, data=rt, time.inc=1)

cal <- calibrate(f, cmethod="KM", method="boot", u=1, m=(nrow(rt)/3), B=1000)

plot(cal, xlim=c(0,1), ylim=c(0,1),

xlab="Nomogram-predicted OS (%)", ylab="Observed OS (%)", lwd=1.5, col="green", sub=F)

f <- cph(Surv(futime, fustat) ~ Nomogram, x=T, y=T, surv=T, data=rt, time.inc=3)

cal <- calibrate(f, cmethod="KM", method="boot", u=3, m=(nrow(rt)/3), B=1000)

plot(cal, xlim=c(0,1), ylim=c(0,1), xlab="", ylab="", lwd=1.5, col="blue", sub=F, add=T)

f <- cph(Surv(futime, fustat) ~ Nomogram, x=T, y=T, surv=T, data=rt, time.inc=5)

cal <- calibrate(f, cmethod="KM", method="boot", u=5, m=(nrow(rt)/3), B=1000)

plot(cal, xlim=c(0,1), ylim=c(0,1), xlab="", ylab="", lwd=1.5, col="red", sub=F, add=T)

legend('bottomright', c('1-year', '3-year', '5-year'),

col=c("green","blue","red"), lwd=1.5, bty = 'n')

dev.off()

#install.packages("survival")

#install.packages("survminer")

library(survival)

library(survminer)

inputFile="timeexp.txt"

gene=""

setwd("C:\\Users\\Ohh\\Desktop\\cuprlnc2\\STEP1EXPRESSION")

rt=read.table(inputFile,header=T,sep="\t",check.names=F)

rt$futime=rt$futime/365

a=ifelse(rt[,gene]<=median(rt[,gene]),"Low","High")

diff=survdiff(Surv(futime, fustat) ~a,data = rt)

pValue=1-pchisq(diff$chisq,df=1)

pValue=signif(pValue,4)

pValue=format(pValue, scientific = TRUE)

fit=survfit(Surv(futime, fustat) ~ a, data = rt)

titleName=gene

surPlot=ggsurvplot(fit,

data=rt,

surv.median.line = "hv",

conf.int=TRUE,

pval=paste0("p=",pValue),

pval.size=6,

#ncensor.plot = TRUE,

legend.labs=c("high","low"),

legend.title=titleName,

xlab="Time(years)",

break.time.by = 1,

risk.table.title="",

palette=c("#dfc223", "#608595"),

risk.table.height=.25)

pdf(file=paste0("sur.",gene,".pdf"), width = 6.5, height = 5.5,onefile = FALSE)

print(surPlot)

dev.off()

library(survival)

library(survminer)

inputFile="PFStimeexp.txt"

gene="ATP7B"

setwd("C:\\Users\\Ohh\\Desktop\\cuprlnc2\\STEP1EXPRESSION")

rt=read.table(inputFile,header=T,sep="\t",check.names=F)

rt$PFI.time=rt$PFI.time/365

a=ifelse(rt[,gene]<=median(rt[,gene]),"Low","High")

diff=survdiff(Surv(PFI.time, PFI) ~a,data = rt)

pValue=1-pchisq(diff$chisq,df=1)

fit=survfit(Surv(PFI.time, PFI) ~ a, data = rt)

pValue=1-pchisq(diff$chisq,df=1)

pValue=signif(pValue,4)

pValue=format(pValue, scientific = TRUE)

titleName=gene

surPlot=ggsurvplot(fit,

data=rt,

surv.median.line = "hv",

conf.int=TRUE,

pval=paste0("p=",pValue),

pval.size=6,

#ncensor.plot = TRUE,

legend.labs=c("high","low"),

legend.title=titleName,

xlab="Time(years)",

ylab="Progression free survival",

break.time.by = 1,

risk.table.title="",

palette=c("#c27f9f", "#087e98"),

risk.table.height=.25)

pdf(file=paste0("PFS.",gene,".pdf"), width = 6.5, height = 5.5,onefile = FALSE)

print(surPlot)

dev.off()

#install.packages("pheatmap")

library(pheatmap)

setwd("C:\\Users\\Ohh\\Desktop\\raw data\\Result 3")

bioRiskPlot=function(inputFile=null, project=null){

rt=read.table(inputFile, header=T, sep="\t", check.names=F, row.names=1)

rt=rt[order(rt$riskScore),]

riskClass=rt[,"risk"]

lowLength=length(riskClass[riskClass=="low"])

highLength=length(riskClass[riskClass=="high"])

lowMax=max(rt$riskScore[riskClass=="low"])

line=rt[,"riskScore"]

line[line>10]=10

pdf(file=paste0(project, ".riskScore.pdf"), width=7, height=4)

plot(line, type="p", pch=20,

xlab="Patients (increasing risk socre)",

ylab="Risk score",

col=c(rep("#608595",lowLength),rep("#dfc286",highLength)) )

abline(h=lowMax,v=lowLength,lty=2)

legend("topleft", c("High risk","Low Risk"),bty="n",pch=19,col=c("#dfc286","#608595"),cex=1.2)

dev.off()

color=as.vector(rt$fustat)

color[color==1]="#dfc286"

color[color==0]="#608595"

pdf(file=paste0(project, ".survStat.pdf"), width=7, height=4)

plot(rt$futime, pch=19,

xlab="Patients (increasing risk socre)",

ylab="Survival time (years)",

col=color)

legend("topleft", c("Dead","Alive"),bty="n",pch=19,col=c("#dfc286","#608595"),cex=1.2)

abline(v=lowLength,lty=2)

dev.off()

ann_colors=list()

bioCol=c("#608595", "#dfc286")

names(bioCol)=c("low", "high")

ann_colors[["Risk"]]=bioCol

rt1=rt[c(3:(ncol(rt)-2))]

rt1=t(rt1)

annotation=data.frame(Risk=rt[,ncol(rt)])

rownames(annotation)=rownames(rt)

pdf(file=paste0(project, ".heatmap.pdf"), width=7, height=4)

pheatmap(rt1,

annotation=annotation,

annotation_colors = ann_colors,

cluster_cols = FALSE,

cluster_rows = FALSE,

show_colnames = F,

scale="row",

color = colorRampPalette(c(rep("#608595",3.5), "white", rep("#dfc286",3.5)))(50),

fontsize_col=3,

fontsize=7,

fontsize_row=8)

dev.off()

}

bioRiskPlot(inputFile="risk.train.txt", project="train")

bioRiskPlot(inputFile="risk.test.txt", project="test")

bioRiskPlot(inputFile="risk.all.txt", project="all")

library(GSVA)

library(limma)

library(GSEABase)

inputFile="symbol.txt"

gmtFile="immune.gmt"

setwd("D:\\biowolf\\Ferroptosis\\30.ssGSEA\\TCGA")

rt=read.table(inputFile,sep="\t",header=T,check.names=F)

rt=as.matrix(rt)

rownames(rt)=rt[,1]

exp=rt[,2:ncol(rt)]

dimnames=list(rownames(exp),colnames(exp))

mat=matrix(as.numeric(as.matrix(exp)),nrow=nrow(exp),dimnames=dimnames)

mat=avereps(mat)

mat=mat[rowMeans(mat)>0,]

geneSet=getGmt(gmtFile, geneIdType=SymbolIdentifier())

ssgseaScore=gsva(mat, geneSet, method='ssgsea', kcdf='Gaussian', abs.ranking=TRUE)

normalize=function(x){

return((x-min(x))/(max(x)-min(x)))}

ssgseaOut=normalize(ssgseaScore)

ssgseaOut=rbind(id=colnames(ssgseaOut),ssgseaOut)

write.table(ssgseaOut,file="ssgseaOut.txt",sep="\t",quote=F,col.names=F)

library(survival)

library(survminer)

setwd("C:\\Users\\Ohh\\Desktop\\cuprlnc2\\STEP6mxsurvival")

bioSurvival=function(inputFile=null, outFile=null){

rt=read.table(inputFile, header=T, sep="\t")

diff=survdiff(Surv(futime, fustat) ~risk,data = rt)

pValue=1-pchisq(diff$chisq,df=1)

pValue=signif(pValue,4)

pValue=format(pValue, scientific = TRUE)

fit <- survfit(Surv(futime, fustat) ~ risk, data = rt)

surPlot=ggsurvplot(fit,

data=rt,

surv.median.line = "hv",

conf.int=T,

pval=paste0("p=",pValue),

pval.size=6,

legend.title="Risk",

legend.labs=c("High risk", "Low risk"),

xlab="Time(years)",

ylab="Overall survival",

break.time.by = 1,

palette=c("#dfc286", "#608595"),

risk.table=F,

risk.table.title="",

risk.table.col = "strata",

risk.table.height=.25)

pdf(file=outFile, width = 6.5, height =5.5, onefile = FALSE)

print(surPlot)

dev.off()

}

bioSurvival(inputFile="risk.train.txt", outFile="surv.train.pdf")

bioSurvival(inputFile="risk.test.txt", outFile="surv.test.pdf")

bioSurvival(inputFile="risk.all.txt", outFile="surv.all.pdf")
