# Supplementary material for: Construction and Validation of a Novel Cuproptosis-Related Seven-lncRNA Signature to Predict the Outcomes, Immunotherapeutic Responses, and Targeted Therapy in Patients with Clear Cell Renal Cell Carcinoma
Source: Dis Markers. 2023 Jan 25;2023:7219794. doi: 10.1155/2023/7219794 (PMC9893525; doi:10.1155/2023/7219794)
Supplement: Supplementary 7 — Figure S6: the ceRNA network of the five cuproptosis-related lncRNAs (orange) and their target miRNAs (light blue) and mRNAs (light green). [file 7219794.f7.pdf]

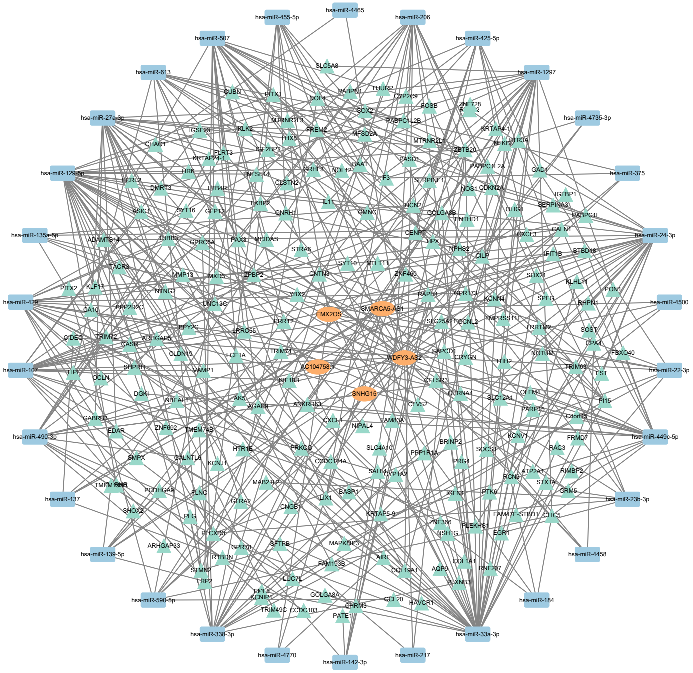

Fig. S6 The ceRNA network of the five cuproptosis-related lncRNAs (orange) and their target miRNAs (light blue) and mRNAs (light green).
